# Selective JAK3 Inhibitors with a Covalent Reversible Binding Mode Targeting a New Induced Fit Binding Pocket

**DOI:** 10.1016/j.chembiol.2016.10.008

**Published:** 2016-11-17

**Authors:** Michael Forster, Apirat Chaikuad, Silke M. Bauer, Julia Holstein, Matthew B. Robers, Cesear R. Corona, Matthias Gehringer, Ellen Pfaffenrot, Kamran Ghoreschi, Stefan Knapp, Stefan A. Laufer

**Affiliations:** 1 Department of Pharmaceutical/Medicinal Chemistry, Eberhard-Karls-University Tuebingen, Auf der Morgenstelle 8, 72076 Tuebingen, Germany; 2 Nuffield Department of Clinical Medicine, Structural Genomics Consortium and Target Discovery Institute, University of Oxford, Old Road Campus Research Building, Roosevelt Drive, Oxford OX3 7DQ, UK; 3 Department of Dermatology, University Medical Center, Eberhard-Karls-University Tuebingen, Liebermeisterstraße 25, 72076 Tuebingen, Germany; 4 Promega Corporation, 2800 Woods Hollow Road, Madison, WI 53711, USA

**Keywords:** JAK3, chemical probe, covalent reversible inhibitor, Janus kinases, kinome selectivity

## Abstract

Janus kinases (JAKs) are a family of cytoplasmatic tyrosine kinases that are attractive targets for the development of anti-inflammatory drugs given their roles in cytokine signaling. One question regarding JAKs and their inhibitors that remains under intensive debate is whether JAK inhibitors should be isoform selective. Since JAK3 functions are restricted to immune cells, an isoform-selective inhibitor for JAK3 could be especially valuable to achieve clinically more useful and precise effects. However, the high degree of structural conservation makes isoform-selective targeting a challenging task. Here, we present picomolar inhibitors with unprecedented kinome-wide selectivity for JAK3. Selectivity was achieved by concurrent covalent reversible targeting of a JAK3-specific cysteine residue and a ligand-induced binding pocket. We confirmed that in vitro activity and selectivity translate well into the cellular environment and suggest that our inhibitors are powerful tools to elucidate JAK3-specific functions.

## Highlights

• Identification and characterization of novel covalent reversible JAK3 inhibitors
• Picomolar affinities along with both high isoform and kinome selectivity is achieved
• Covalent-reversible interaction and a new induced binding pocket confirmed by X-ray structures
• High potency and selectivity are successfully proven in cellular models

## Graphical Abstract

**Figure:**
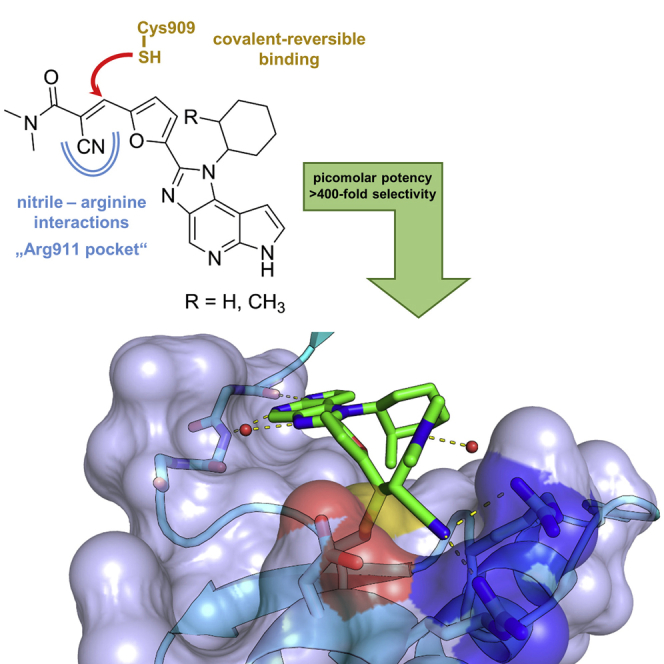

## Teaser

JAK1/3 signaling plays a major role in immunological and inflammatory processes, but the reciprocal dependency of this receptor-sharing kinase pair still remains unclear. Forster et al. report highly selective covalent reversible JAK3 inhibitors as promising tools to elucidate this issue.

## Introduction

**Figure 1:**
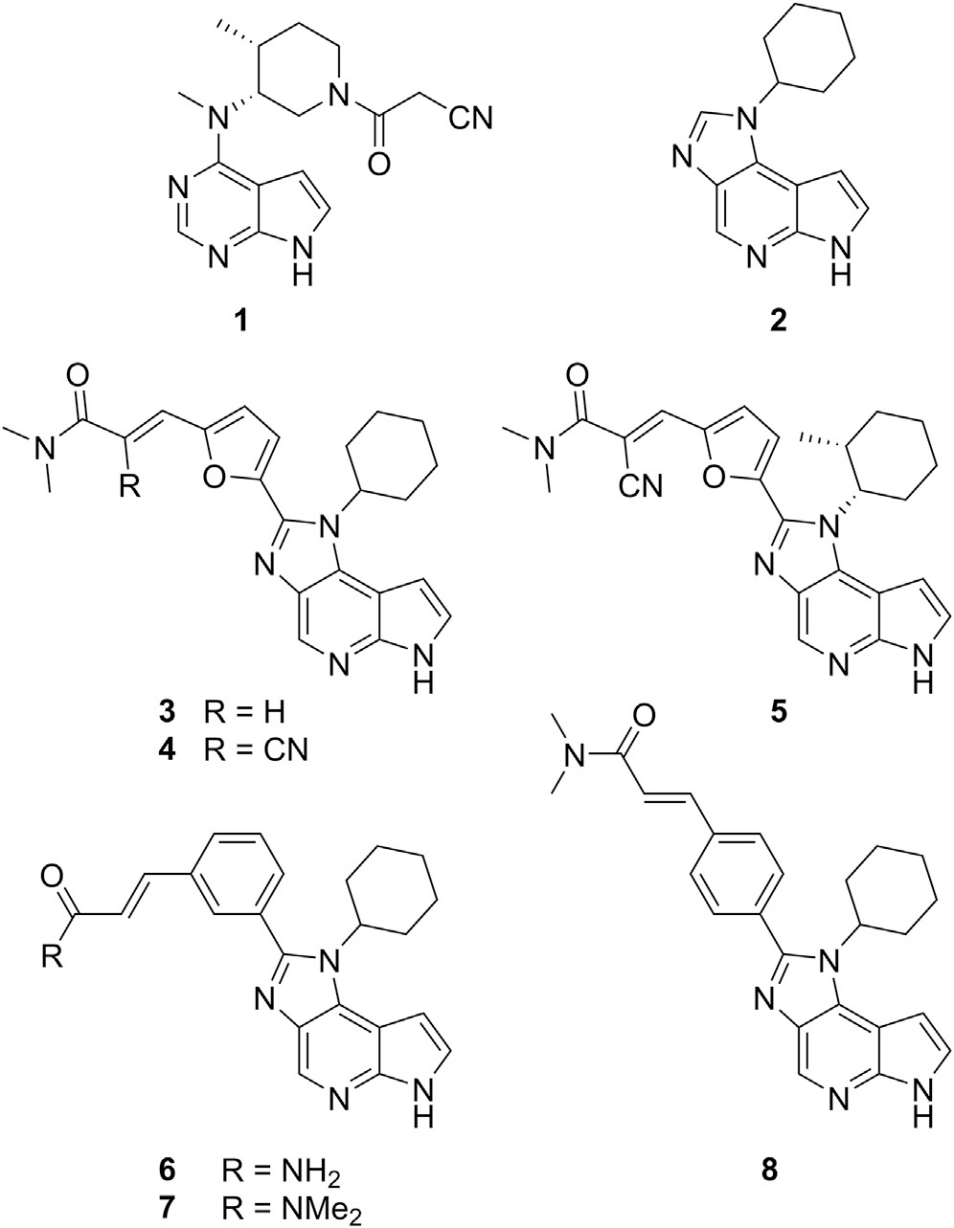
Structures of Tofacitinib **1**, Lead Compound **2**, and Novel JAK3 Inhibitors **3**–**8** See also Tables S1, S2, S3, and S6.

While the other isoforms of the Janus kinase family (JAK1, JAK2, and TYK2) have a broad spectrum of functions in different tissues, JAK3 plays a specific role in the development of immune-competent cells (Ghoreschi et al., 2009). The key function of JAK3 in the immune system is further supported by the fact that loss-of-function mutations of JAK3 cause severe combined immunodeficiency syndrome (SCID). Therefore, JAK3-selective inhibitors are considered as promising candidates for immunosuppressive and anti-inflammatory therapies (Pesu et al., 2005). However, the sufficiency of specific JAK3 inhibition for efficient immunosuppression is heavily debated. The cause of the controversy rests on the invariable co-localization of JAK3 and JAK1 on common γ chain (γ_c_) cytokine receptor dimers, suggesting that dual JAK1 and JAK3 inhibition is required for efficient suppression of cytokine signaling (Haan et al., 2011, Thorarensen et al., 2014). To resolve this enigma, highly JAK3-selective chemical probes are required. To date, only a few compounds with appropriate isoform selectivity for JAK3 have been reported. The current gold standard for investigating JAK-dependent signaling is the selective pan-JAK inhibitor tofacitinib (**1**, Figure 1) (Knapp et al., 2013). Although initially claimed as JAK3 specific (Flanagan et al., 2010), further studies demonstrated the poor selectivity of **1** within the JAK family (Thoma et al., 2014). Due to the immense structural conservation among the JAKs, isoform selectivity remains a challenging task. Thoma et al. (2011) developed reversible maleinimide-derived inhibitors with reasonable JAK3 selectivity (>120 fold), which have been utilized with limited success to probe the JAK1/3 dependency issue (Haan et al., 2011). Very recently, Goedken et al. (2015) and Tan et al. (2015) reported irreversible JAK3 inhibitors showing good isoform selectivity but also potent off-target activity in the remaining kinome. Merck patented several irreversible acrylamide-based inhibitors (Ahearn et al., 2013), and recently Smith et al. (2016) utilized an inhibitor from this structural class for comprehensive investigation of the time dependency of JAK1/3 signaling in T cells. Although high JAK3 isoform selectivity was demonstrated, kinome-wide selectivity was not particularly investigated. Furthermore, the collective consequences of JAK3 inhibition in an exclusively irreversible covalent manner are still unclarified and may not be favorable for all purposes. An alternative to classical Michael acceptors are covalent reversible cyano-acrylamide-based inhibitors. This principle was successfully applied by London et al. (2014) to obtain JAK3 inhibitors with reasonable in vitro activity and isoform selectivity, but without a proven cellular activity and kinome-wide selectivity. The present study reports a novel class of covalent reversible JAK3 inhibitors providing both high isoform and kinome selectivity as well as potent cellular activity and selectivity.

## Results and Discussion

### Identification of Covalent Reversible JAK3 Inhibitors **4** and **5**

In our quest for a highly selective JAK3 probe, we aimed to exploit a non-catalytic cysteine (C909), which is not present in the other JAK family members. C909 is situated in the solvent-exposed front part of the ATP binding site, where the other isoforms possess a serine residue (Chrencik et al., 2010). The nucleophilic nature of the cysteine thiol group can be utilized to covalently trap inhibitors bearing an electrophilic group (Singh et al., 2010). Besides JAK3, only ten human kinases feature a cysteine at an equivalent position (Liu et al., 2013).

Based on modeling studies, we identified compound **2** (Figure 1) with a half maximal inhibitory concentration (IC_50_) of 63 nM as a reasonable starting point for the development of covalent JAK3 inhibitors targeting C909. We substituted the imidazole C2 atom with suitable linker moieties bearing an electrophilic warhead. Initial efforts focused on *para*- and *meta*-substituted phenyl linkers and simple acrylamides as Michael acceptors. This strategy provided limited success since all compounds (**6–8**, Figure 1) in this series exhibited decreased inhibitory activity compared with **2** (Table S1). Switching the linker from phenyl to a 2,5-disubstituted furyl moiety furnished more promising results. Compound **3** (Figure 1) bearing a classical acrylamide Michael acceptor demonstrated a slightly higher inhibitory activity (IC_50_ = 51 nM) than template **2**. However, the increase in activity was not substantial enough to assume covalent binding. Accordingly, the initial design strategy was revised in two ways. First, the reactivity of the Michael acceptor was tuned by introducing an additional nitrile group at the αC atom. Cyano-acrylamides were postulated as covalent reversible Michael acceptors, since the covalent bond formation can be reversed under physiological conditions (Serafimova et al., 2012). As a second feature, an additional methyl group was introduced at position 2 of the cyclohexyl moiety to mimic the chiral side chain of **1** more appropriately. Both modifications were well tolerated and yielded highly potent compounds **4** and **5** (Figure 1) with IC_50_ values of 9 nM and 17 nM, respectively, which are close to the lower detection limit of our ELISA.

### Compounds **4** and **5** Demonstrate High JAK Isoform and Kinome Selectivity

**Figure 2:**
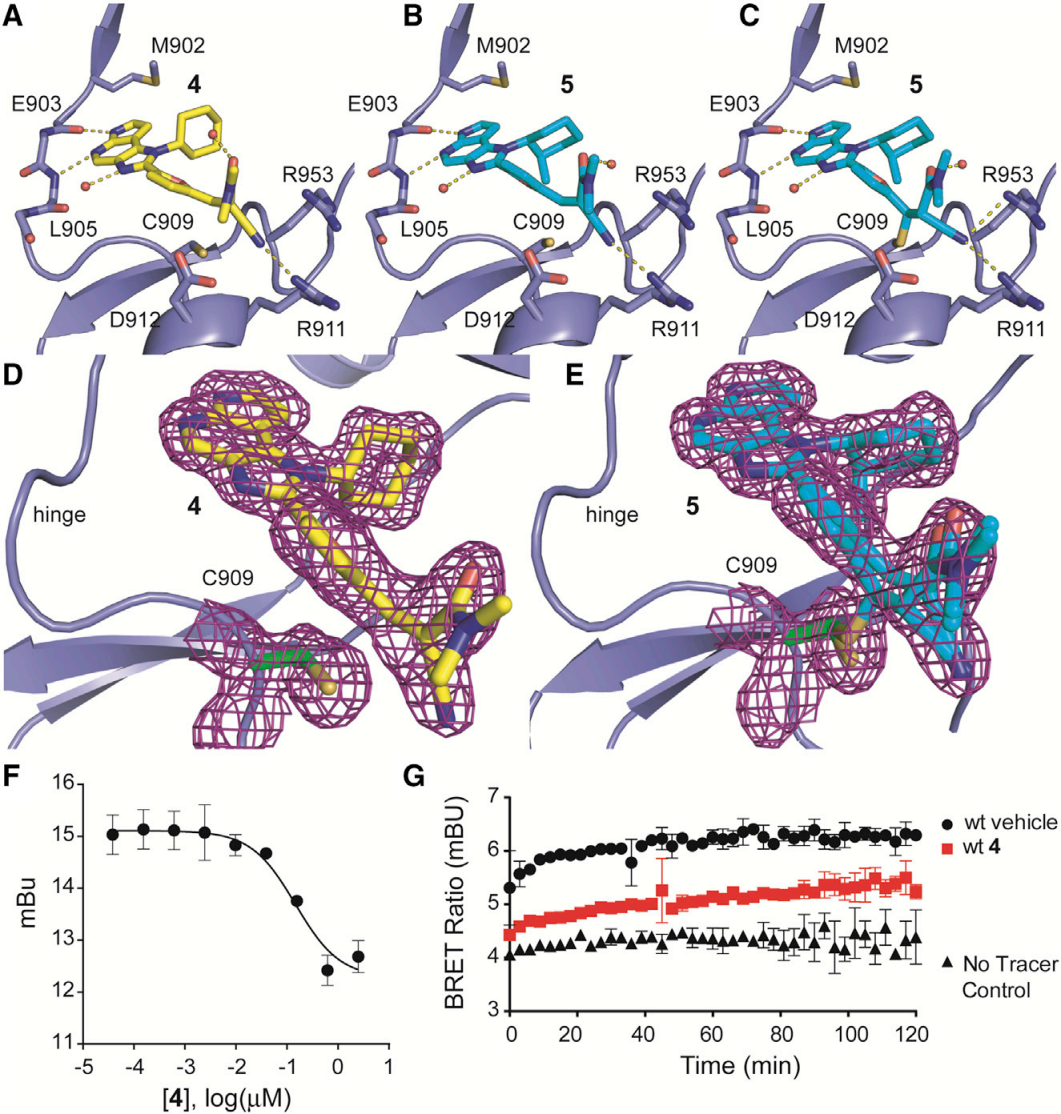
Co-crystal Structures of JAK3 and Compounds **4** and **5** (A) **4** non-covalently bound to JAK3 (PDB: 5LWM). (B) **5** non-covalently bound to JAK3 (PDB: 5LWN). (C) **5** covalently bound to JAK3 (PDB: 5LWN). (D) 2F_o_ − F_c_ omitted electron density map of **4**-JAK3. (E) 2F_o_ − F_c_ omitted electron density map of **5**-JAK3. (F) Dose-dependent BRET experiment showing displacement of the fluorescent tracer in NanoLuc tagged JAK3 in HeLa cells. (G) Residence time experiment using BRET. HeLa cells expressing NanoLuc JAK3 were equilibrated with 1 μM of **4**, washed out, and treated with high concentrations of tracer. Displacement of **4** was monitored by BRET. BRET levels of full occupancy control were reached after approx. 1 hr. Data shown are means ± SD of quadruplicates. See also Figures S1, S2, and Table S4.

This 4- to 7-fold increase in JAK3 inhibitory activity compared with **2** prompted us to determine the JAK isoform selectivity for compounds **1**, **3**, **4**, and **5** in a commercial assay (Kinase HotSpot, Reaction Biology Corp.). As previously observed (Gehringer et al., 2014), this assay format is more sensitive and therefore provides comparatively lower IC_50_ values than the previously applied ELISA (Table S1). The selectivity profiles of **1** and the classical amide-derived Michael acceptor **3** are significantly different from cyano-acrylamides **4** and **5**. While **3** and **1** were reasonably potent (IC_50_ = 22 nM and 292 pM, respectively) but unselective within the JAK family, both **4** and **5** exhibited JAK3 IC_50_ values in the picomolar range (127 pM and 154 pM, respectively), even lower than the reference compound **1**, and demonstrated 400-, 2,700- and 3,600-fold or 400-, 1,700-, and 5,800-fold selectivity over JAK1, JAK2, and TYK2, respectively (Table S1). We screened **4** and **5** against a panel of 410 kinases (Kinase 410-Profiler, ProQinase) at concentrations of 100 nM and 500 nM. Both compounds had no relevant effect on the activity of any tested kinases except JAK3 at a concentration of 100 nM. At 500 nM, compound **4** moderately inhibited 11 other kinases besides JAK3 with residual activities below 50%, while compound **5** revealed only one off-target (Table S2). The somewhat better selectivity profile of **5** can be attributed to the additional exocyclic methyl group, which is supposed to be also the key driver for the exceptional kinome selectivity of **1** (Chrencik et al., 2010). It is noteworthy that both compounds show no significant activity against the other ten kinases carrying a cysteine at the equivalent position. To verify these results in a cellular setting, we used a bioluminescence resonance energy transfer (BRET) assays in HeLa cells expressing the NanoLuc fused tyrosine kinases BTK, BLK, and TEC, which all harbor a cysteine at the same position. We detected no measurable interaction of **4** with TEC or BTK, and only BLK interacted weakly at micromolar concentrations (Figures S1D–S1F). Thus, **4** and **5** represent two excellent tool compounds that compare favorably with other JAK3 inhibitors published in the current peer-reviewed literature (Table S6). We also determined binding kinetics of **4** on all JAK isoforms (Proteros Reporter Displacement Assay, Proteros Biostructures). While **4** rapidly diffuses from JAK1, JAK2, and TYK2 with residence times below 1.4 min (lower detection limit), a prolonged residence time of 50 min on JAK3 was observed (Table S3). We used BRET to reveal the binding characteristics of **4** in living cells. In dose-response experiments, we observed efficient displacement of the fluorescent tracer at around 100 nM (Figure 2F), demonstrating on-target activity and good cellular activity of **4**. In washout experiments we also determined the dissociation behavior of **4** and observed recovery of the BRET ratio after about 1 hr in agreement with the binding kinetic experiments described above (Figure 2G). This combination of inhibitory and kinetic JAK3 selectivity reinforced our assumption of covalent reversible binding of **4** and **5**.

### JAK3 Co-crystal Structures of **4** and **5** Confirm Covalent Reversible Binding and Reveal a New Binding Pocket

**Figure 3:**
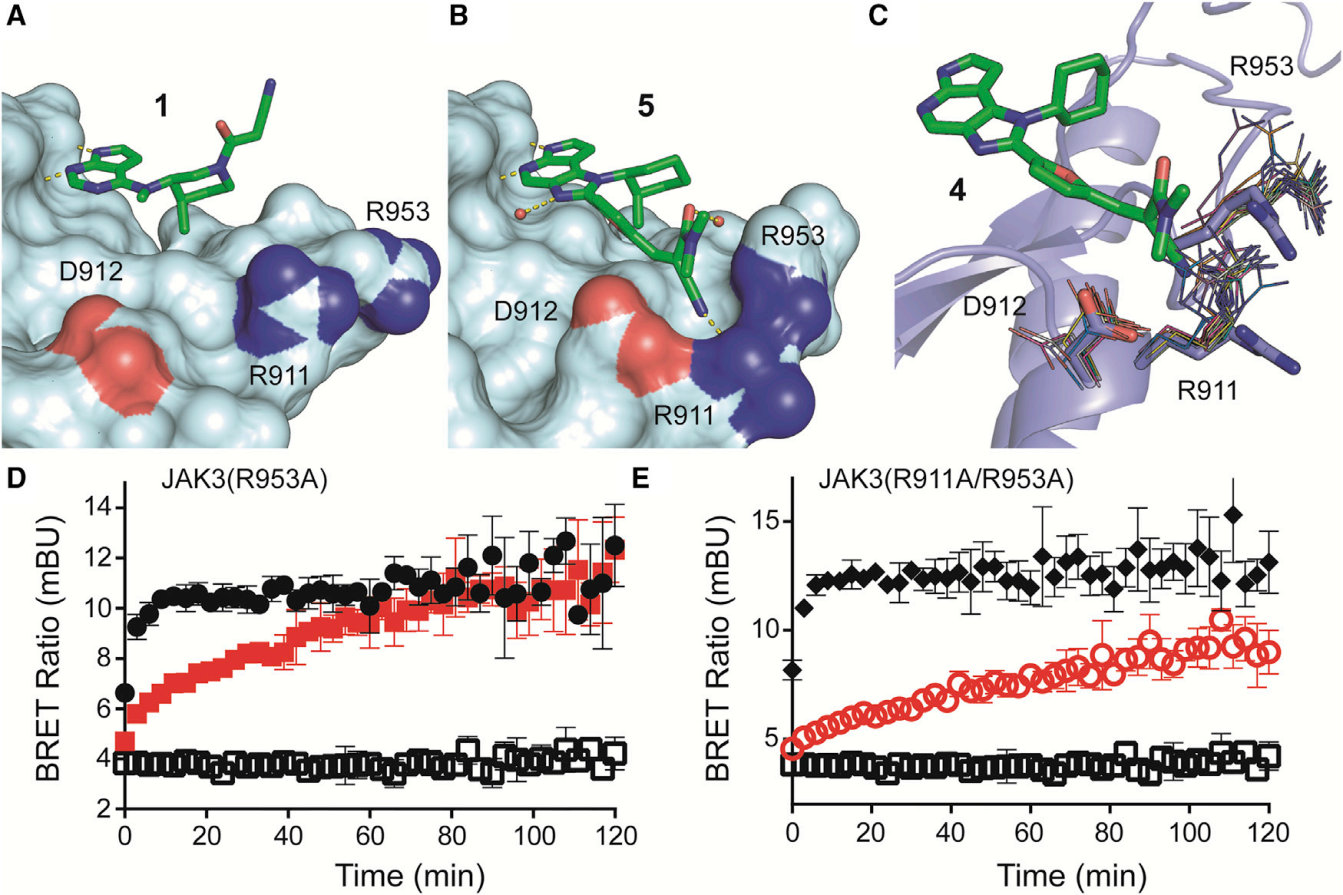
Induced Binding Pocket Around R911 (A) Protein surface of **1** bound to JAK3 (PDB: 3LXK). (B) Protein surface of **5** bound to JAK3 (PDB: 5LWN). N-terminal lobes are omitted for clarity and heteroatoms of the residues of R911, D912, and R953 are colored. Comparison of (A) and (B) shows the rearrangement of these amino acid side chains upon formation of the arginine pocket. (C) Alignment of 16 JAK3 crystal structures with **4**-JAK3. Residues of R911, D912, and R953 are shown as sticks (**4**-JAK3, PDB: 5LWM) or as lines (other structures, PDB: 3LXK, 4QT1, 4QPS, 4RIO, 4ZEP, 4I6Q, 3ZC6, 4HVD, 4HVG, 4HVH, 4HVI, 3PJC, 3LXL, 1YVJ, 4V0G, 4Z16). The deviating conformation of these side chains is unique to our structure, while it is relatively conserved among the other JAK3 structures. (D and E) BRET experiments measuring residence time of **4** in HeLa cells expressing the NanoLuc mutant JAK3 R953A (D) or the NanoLuc double mutant JAK3 R911A/R953A (E). BRET traces of vehicle-treated cells are shown as filled black spheres (D) and diamonds (E), traces of inhibitor-treated cells are shown in red, and no tracer control is shown as empty squares. Washout experiments show that both mutants retain slow binding kinetics of **4**. Data shown are mean ± SD of quadruplicates. See also Figure S1, Tables S4, and S5.

High resolution crystal structures of **4** and **5** in complex with JAK3 were determined (Figure 2). Both compounds showed the expected orientation of the hinge binding motif featuring the typical bidentate hydrogen bonding pattern. While the complex of JAK3 with **4** only revealed the non-covalent binding mode, the structure in complex with **5** displayed the coexistence of the non-covalently and the covalently bound inhibitor **5** (Figures 2A–2C). The presence of both binding modes underlines the highly reversible character of the covalent interaction since the crystal structure represents an equilibrium state between covalently and non-covalently bound **5**. The coexistence of both binding modes in the crystal structure with **5** was demonstrated by difference electron density maps after refinement (Figure S2), considering only the reversible, the irreversible, or both binding modes, and confirmed by electrospray ionization mass spectrometry (ESI-MS) (Figure S2). Although three JAK3 crystal structures with irreversible inhibitors have recently been published (PDB: 4QPS, 4Z16, 4V0G), the structural model presented here is the first one depicting the interaction of a covalent reversible inhibitor with JAK3 as highlighted by electron density maps that clearly distinguish between covalent and non-covalent binding modes (Figures 2D and 2E). To the best of our knowledge, the simultaneous presence of both binding modes has not been observed for any cyanoacrylamide-derived inhibitor before. Therefore, our structure further validates the concept of covalent reversible enzyme inhibition with Michael acceptors. Furthermore, we observed a yet unprecedented binding pocket formed by R911, D912, and R953. This unique feature is induced by interactions of the nitrile moieties of **4** and **5** with R911, thereby generating a distinct cavity in the protein surface (Figure 3B). Structural comparison with other JAK3 structures reveals that the side chain of R911 is dramatically reoriented and forms a hydrogen bond with the inhibitors nitrile and nitrogen. The guanidine moiety of R953 is flipped approximately 180° toward the nitrile group of **4** and thereby forms the ceiling of this arginine pocket (Figures 3A and 3B). Alignment of the other 16 available JAK3 crystal structures revealed that the orientation of the aforementioned arginine residues is conserved in those structures, confirming the induction of the arginine pocket to be a unique feature of our inhibitor class (Figure 3C). To assess the contribution of this cavity to the outstanding selectivity of **4** and **5**, we aligned the amino acid sequence of the arginine pocket in JAK3 with the corresponding regions of kinases carrying a reactive cysteine at an equivalent position (Table S5). While the R953 (JAK3 numbering) is mainly conserved, JAK3-D912 is replaced by an asparagine, glutamate, or lysine in five of the ten other kinases. Interestingly, an arginine residue at position 911 is unique to JAK3. In most of the other kinases, this position is occupied by a bulkier and less flexible leucine residue incapable of forming polar interactions. In contrast, the amino acids forming the arginine pocket are mainly conserved in the JAK family (Table S5) except for JAK1, possessing a lysine residue instead of R911 and a glutamate instead of D912. Based on these observations, we assume that the interaction of JAK3 R911 with the nitrile substituent of compounds **4** and **5** constitutes a second key feature for inhibitor selectivity and potency. BRET experiments using the JAK3 mutant R953A, as well as the double mutant R911A/R953A, showed similar affinity of **4** (Figures S1A–S1C), suggesting that the induction of the arginine cavity did not significantly affect inhibitor potency. Moreover, the inhibitor dissociating rates of **4** in the two JAK mutants R953A and R911A/R953A were comparable with the ones observed in the wild-type protein (Figures 3D and 3E). However, the induced pocket as well as the hydrogen bonds formed with **4** and **5** in combination with the covalent cysteine targeting represent a unique dual selectivity filter of these inhibitors outside the JAK family (Muller et al., 2015).

### Compounds **4** and **5** Selectively Inhibit JAK3 Signaling in Human CD4^+^ T Cells

**Figure 4:**
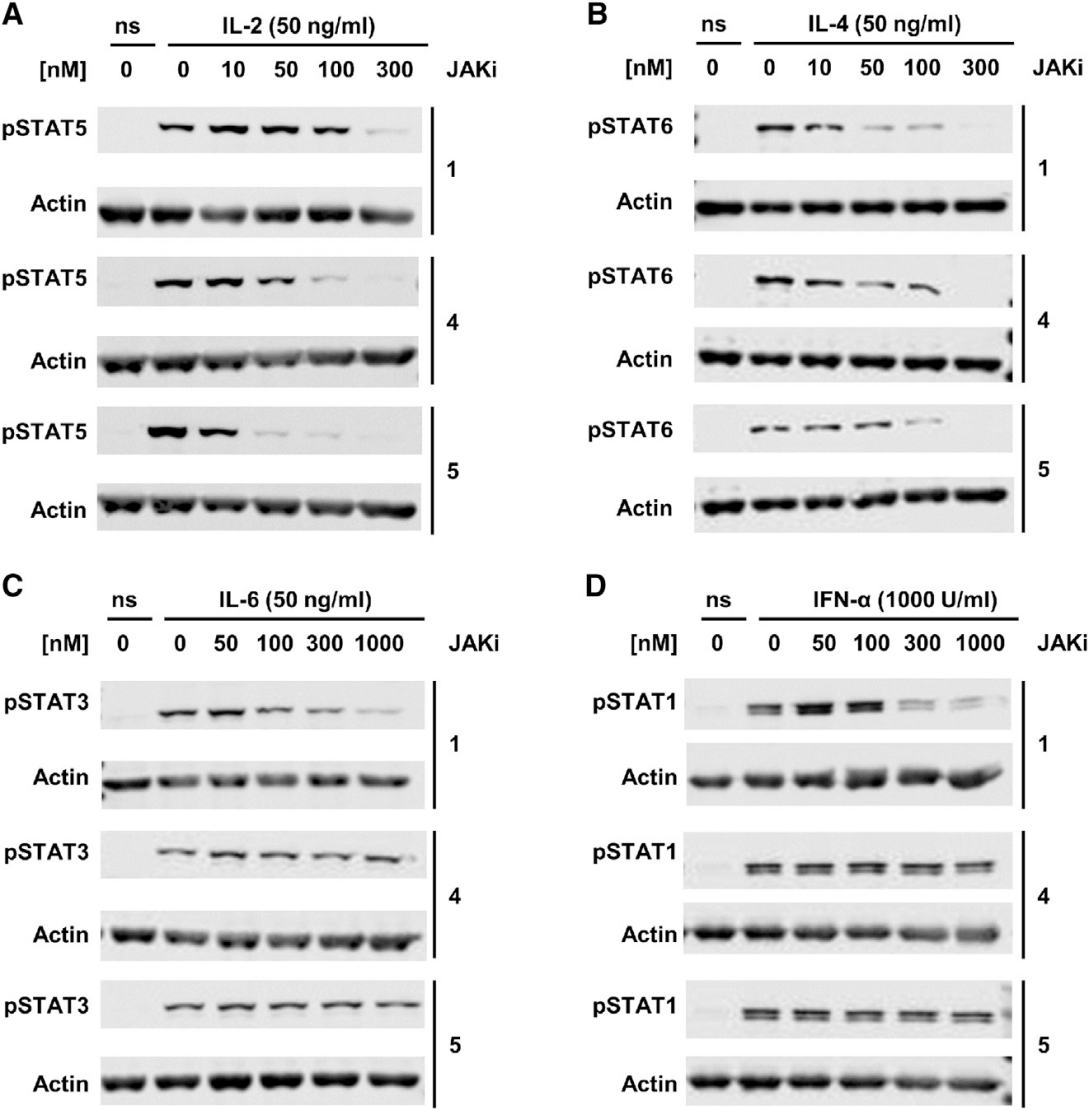
Selective Inhibition of JAK3-Mediated Cytokine Signaling by Compounds **4** and **5** Human CD4^+^ T cells were pre-incubated for 1 hr with the indicated concentrations of the JAK inhibitors (JAKi) **1**, **4** or **5** and stimulated for 30 min with IL-2 (activates JAK3/JAK1) (A), IL-4 (activates JAK3/JAK1) (B), IL-6 (activates JAK2/JAK1/TYK2) (C) or IFN-α (activates JAK1/TYK2) (D). Phosphorylation of STAT5 (A), STAT6 (B), STAT3 (C), or STAT1 (D) was determined by phospho-specific Abs and immunoblotting (ns, no cytokine stimulation). Levels of actin were determined to show equal loading. See also Table S1.

To assess the functional selectivity of **4** and **5**, we used functional human CD4^+^ T cells that were stimulated with cytokines activating the JAK/STAT pathway. First, we stimulated the T cells possessing the γ_c_ receptor using interleukin (IL)-2. Consecutive activation of JAK3 and JAK1 and subsequent STAT5 phosphorylation allowed comparison of **4** and **5** with the clinically established pan-JAK inhibitor **1**. Compounds **4** and **5** abrogated γ_c_ cytokine signaling even at 50 nM or 100 nM, respectively, while a higher concentration (300 nM) of **1** was necessary to completely block STAT5 phosphorylation (Figure 4A). A similar result was observed when stimulating T cells with IL-4, where all three compounds completely block the JAK1/JAK3-mediated STAT6 phosphorylation at 300 nM (Figure 4B). As reported earlier (Ghoreschi et al., 2011), STAT3 phosphorylation via JAK1, JAK2, and TYK2, triggered by stimulation with IL-6, is inhibited by **1** in a dose-dependent manner at concentrations ≥300 nM. In sharp contrast, **4** and **5** did not affect STAT3 activation at doses up to 1,000 nM (Figure 4C), confirming the selectivity toward JAK3 in functional cells. The selectivity difference between our compounds and **1** was even more pronounced when T cells were stimulated with interferon (IFN)-α. While **1** clearly inhibits IFN-α-mediated JAK1/TYK2 signaling at concentrations >100 nM, compounds **4** and **5** did not influence pSTAT1 levels at concentrations up to 1,000 nM (Figure 4D). These results demonstrate that the JAK3 selectivity of **4** and **5** observed in enzyme assays are maintained in a cellular context.

## Significance

**In this study, a new class of covalent reversible JAK3 inhibitors was developed. Crystallographic data confirmed a reversible covalent binding mode as well as a previously unseen binding cavity induced by a nitrile-arginine interaction. The combination of both structural features paved the way for a new generation of JAK3-selective inhibitors. With compounds 4 and 5, we provide JAK3 inhibitors with picomolar affinities and outstanding selectivity within the JAK family and against the whole kinome. Moreover, it was demonstrated that activity and selectivity translate well in a cellular environment. These compounds are thus suitable to serve as chemical probes to elucidate the effect of selective JAK3 inhibition. They might be used for studying the JAK1/3 interplay with particular regard to their respective roles in cytokine signaling and the resultant clinically relevant immunosuppressive effects.**

## Experimental Procedures

### Chemical Synthesis

Synthetic schemes and detailed synthetic procedures are described in the Supplemental Experimental Procedures.

### JAK3 ELISA

For initial JAK3 IC_50_ determination, different concentrations of inhibitors were incubated in substrate-coated 96-well plates with ATP and recombinant JAK3 kinase domain (amino acids 781–1124). The degree of phosphorylation was determined by detection via monoclonal anti-pTyr-HRP-conjugated antibodies, followed by a color reaction. See the Supplemental Experimental Procedures for details and Bauer et al. (2014).

### Protein Expression, Purification, Crystallization, and Structure Determination

Recombinant JAK3 kinase domain with a tobacco etch virus (TEV)-cleavable His tag was expressed in Sf9 cells. The cells were lysed and protein was initially purified by Ni-affinity chromatography. Protein was incubated overnight with the inhibitors and TEV protease. The cleaved protein was further purified by reverse Ni-affinity and size exclusion chromatography. JAK3-inhibitor complexes were crystallized using sitting-drop vapor diffusion. The obtained crystals were cryoprotected and diffraction data were collected at Diamond Light Source. More details on the procedures and crystallographic data refinement are described in the Supplemental Experimental Procedures.

### BRET Experiments

Dose-response experiments were conducted in HeLa cells expressing NanoLuc JAK3 or the studied mutants using Promega tracer 5. Binding kinetic experiments in living cells after compound washout were conducted using the same constructs and tracers as described in Robers et al. (2015). A detailed description is provided in the Supplemental Experimental Procedures.

### CD4^+^ T Cell Cytokine Stimulation Assays

T cells were purified from peripheral blood mononuclear cells from human donors. Equal numbers of cells were incubated for 1 hr with JAK inhibitors or DMSO control and stimulated with cytokines for 30 min. The cells were lysed, and the proteins were separated via PAGE and transferred to a polyvinylidene fluoride membrane. The proteins of interest were blotted with specific antibodies and visualized with an infrared imaging system. A detailed description can be found in the Supplemental Experimental Procedures.

## Author Contributions

S.A.L. and S.K. initiated and supervised this study. M.F. and M.G. conceived the chemical experiments and M.F. carried them out. A.C. conceived the protein X-ray experiments and carried them out. S.M.B. conceived the experiments for the initial biological evaluation and carried them out. M.B.R. and C.R.C. conceived and carried out the BRET experiments. J.H. and K.G. conceived the CD4^+^ T cell experiments and J.H. carried them out. M.F., A.C., M.G., S.M.B., and J.H. carried out the data analysis. M.F., M.G., S.M.B., E.P., A.C., K.G., and S.K. wrote the paper.

## Accession Numbers

The accession number for compound **4** co-crystallized with JAK3 is PDB: 5LWM. The accession number for compound **5** co-crystallized with JAK3 is PDB: 5LWN.

## Supplemental Information

Document S1. Supplemental Experimental Procedures, Figures S1 and S2, Tables S1, S3–S6

Table S2. Kinome Selectivity of **4** and **5**, Related to Figure 1

Document S2. Article plus Supplemental Information
