# Supplementary material for: Selective JAK3 Inhibitors with a Covalent Reversible Binding Mode Targeting a New Induced Fit Binding Pocket
Source: Cell Chem Biol. 2016 Nov 17;23(11):1335–40. doi: 10.1016/j.chembiol.2016.10.008 (PMC5119931; doi:10.1016/j.chembiol.2016.10.008)
Supplement: Document S1. Supplemental Experimental Procedures, Figures S1 and S2, Tables S1, S3–S6 [file mmc1.pdf]

Cell Chemical Biology, Volume *23*

Supplemental Information

Selective JAK3 Inhibitors with a Covalent Reversible Binding Mode Targeting a New Induced Fit Binding Pocket

Michael Forster, Apirat Chaikuad, Silke M. Bauer, Julia Holstein, Matthew B. Robers, Cesear R. Corona, Matthias Gehringer, Ellen Pfaffenrot, Kamran Ghoreschi, Stefan Knapp, and Stefan A. Laufer

Supplemental Data Items

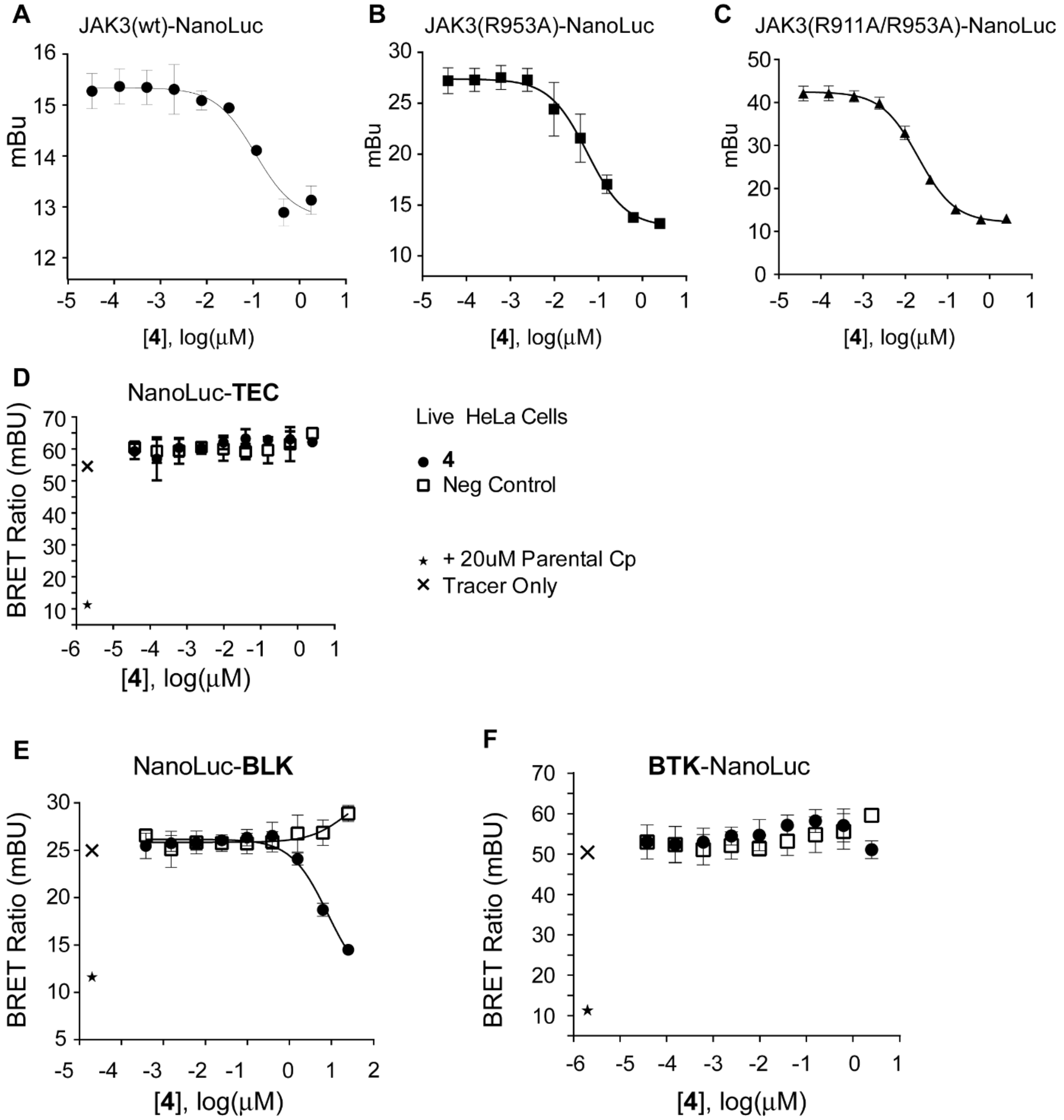

Figure S1, related to Figure 2 and 3. Additional cellular assays data for compound 4 (**A**) nBRET assay data measured on wild type JAK3. (**B**) nBRET assay data measured on the JAK3 mutant R953A. (**C**) nBRET data measured on the JAK3 double mutant R953A/R911A. (**D**) nBRET assay data measured on the tyrosine kinase TEC (TEC receptor tyrosine kinase). (**E**) nBRET assay data measured on the tyrosine kinase BLK (B lymphocyte kinase). (**F**) nBRET assay data measured on the tyrosine kinase BTK (Brutons tyrosine kinase). The figures **D-E** show dose response data with compound **4**, the negative control (DMSO) as well as displacement by the tracer parental compound (*) and the tracer alone (x). All experiments show average data of four experiments as well as the standard deviation for each data point.

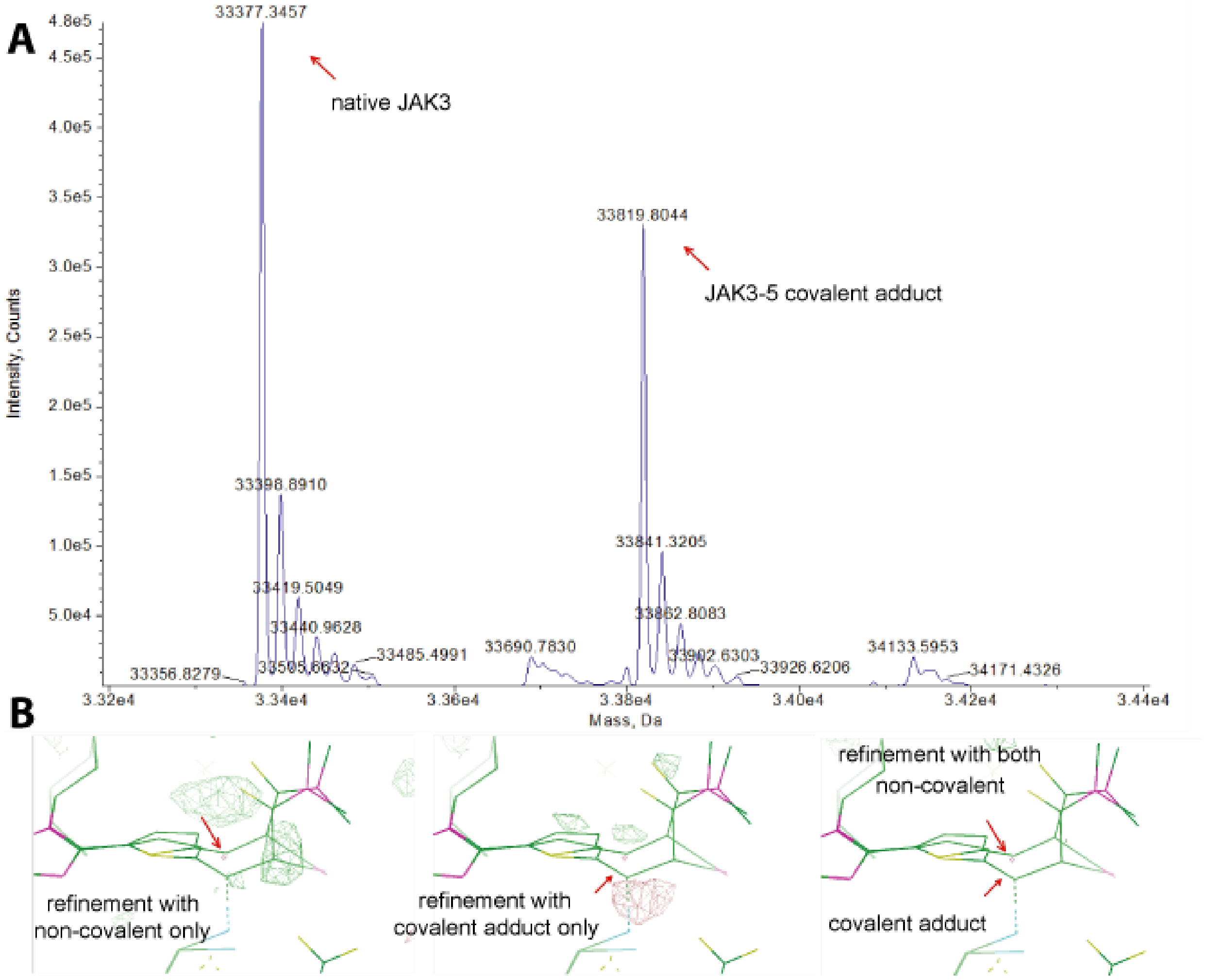

Figure S2, related to Figure 2. Additional data on the binding mode of 5 to JAK3. (**A**) ESI-TOF-MS spectra of JAK3 treated overnight with compound **5** at three-fold excess at 4 ºC. The covalent binding mode of the inhibitor is evident by the mass shift of the protein. (**B**) FoFc difference density maps showing either positive (green) (additional density not accounted for by the model) as well as negative (red) electron density (surplus density created by incorrectly placed atoms of the model) investigating the covalent as well as non-covalent binding modes of compound **5**.

Table S1, related to Figure 1 and 4. Initial JAK3 inhibition ELISAs and JAK isoform selectivity assays at the isolated enzymes.

Compd.	JAK3 IC_{50} [nM][a]	IC_{50} [nM][b]			
		JAK1	JAK2	JAK3	TYK2
**1**	3.5 ± 0.6	0.496	2.2	0.292	8.9
**2**	63 ± 9	-	-	-	-
**3**	51 ± 6	19	113	22	78
**4**	9 ± 1	52	346	0.127	459
**5**	17 ± 1	64	270	0.154	898
**6**	470 ± 36	-	-	-	-
**7**	548 ± 39	-	-	-	-
**8**	226 ± 31	-	-	-	-

[a]IC_{50} values are calculated from the results of a ELISA (Bauer et al., 2014). Data represent the average ± SEM (n = 3) for **2-8** and average ± SD (n = 18) for **1**. [b]IC_{50} values were calculated from the results of a radiometric assay. Data were obtained as 5-dose singlicate IC_{50} value with 10-fold serial dilution starting at 1 µM. [ATP] = 10 µM. Tofacitinib citrate (**1**) was purchased from Shanghai BOC Chemical Co., Ltd.

Table S2, related to Figure 1. Kinome selectivity of 4 and 5, see additional excel file.

Table S3, related to Figure 1. Kinetic profiling of Compound 4

kinase	K_D [µM]	k_{on} [1/s 1/M][a]	k_{off} [1/s][a]	residence time [min][b]
**JAK1**	6.97E-02	n.a.	n.a.	< 1.4
**JAK2**	2.42E-01	n.a.	n.a.	< 1.4
**JAK3**	1.34E-03	2.47E+05	3.30E-04	50
**TYK2**	3.60E-01	n.a.	n.a.	< 1.4

[a]n.a. = below detection limit; [b]< 1.4 = lower detection limit; Data was obtained from a commercial displacement assay. Displacement of a reporter probe was measured time dependent for different concentrations of the tested compound. Detailed methodology are described in the Supplemental Experimental Procedures.

Table S4, related to Figures 2 and 3. Xray data collection and refinement

	**4**-JAK3	**5**-JAK3
**Data collection**		
Space group	*P* 2_1	*P* 2_1
Cell dimensions		
a, *b*, *c* (Å)	42.0, 62.5, 51.3	42.0, 62.3, 51.1
α, *β*, *γ* (°)	90.0, 92.9, 90.0	90.0, 92.6, 90.0
Resolution (Å)	39.60-1.55 (1.63-1.55)*	62.57-1.60 (1.69-1.60)
R_{merge}	0.073 (0.722)	0.082 (0.680)
$I / \sigma I$	11.3 (2.7)	9.9 (2.2)
Completeness (%)	99.7 (99.4)	100.0 (100.0)
Redundancy	5.9 (5.6)	6.0 (6.0)
**Refinement**		
PDB-ID:	5LWM	5LWN
Resolution (Å)	39.60-1.55 (1.63-1.55)*	62.57-1.60 (1.69-1.60)
No. reflections	38,357 (5,553)	35,006 (5,106)
R_{work} / R_{free}	0.163/ 0.204	0.166/ 0.209
No. atoms		
Protein	2,333	2,352
Compounds (4 or 5)	32	66
Water and solvents	247	228
B factors (Å^2)		
Protein	27	30
Compounds (4 or 5)	25	24
Water and solvents	37	38
r.m.s. deviations		
Bond lengths (Å)	1.6	1.6
Bond angles (°)	0.016	0.016

*Values in parentheses are for highest-resolution shell.

Table S5, related to Figure 3. Amino acid setup involved in formation of the arginine-pocket

Kinases with equivalent cysteine to Cys909 in JAK3

Kinase	UNIPROT Entry #	begin ATP binding site	reactive cysteine	Arginine-pocket residues		
**JAK3**	P52333	Leu828	Cys909	Arg911	Asp912	Arg953
**MAP2K7** (MKK7)	O14733	Met126	Cys202	Glu204	Lys205	Pro246
**TEC**[a]	P42680	Leu376	Cys449	Leu451	Asp452	Arg493
**TXK**[a]	P42681	Ile277	Cys350	Leu352	Asn353	Arg494
**ITK**	Q08881	Ile369	Cys402	Ser444	Asp445	Arg486
**BTK**	Q06187	Leu407	Cys481	Leu483	Asn484	Arg925
**BMX**	P51813	Leu423	Cys496	Leu498	Asn499	Arg540
**BLK**[a]	P51451	Leu246	Cys319	Leu321	Asp322	Arg362
**HER2** (ERBB2)	P04626	Leu726	Cys805	Leu807	Asp808	Arg849
**EGFR**	P00533	Leu718	Cys797	Leu799	Asp800	Arg841
**HER4** (ERBB4)	Q15303	Leu724	Cys803	Leu805	Glu806	Arg847
**JAK-Family**						
**JAK1**	P23458	Leu881	Ser963	Lys965	Glu966	Arg1007
**JAK2**	O60674	Leu855	Ser936	Arg938	Asp939	Arg980
**JAK3**	P52333	Leu828	Cys909	Arg911	Asp912	Arg953
**TYK2**	P29597	Leu903	Ser985	Arg987	Asp988	Arg1027

The reactive cysteine is usually located around 70 positions in C-terminal direction from the begin of the ATP-binding site. Arg911 and Asp912 positions follow 2 and 3 amino acids later. The position of Arg953 is usually situated around 40 positions later.

[a]for these kinases no PDB structure was available (february 2016)

Table S6, related to Figure 1. Comparison of 4 and 5 with other literature known JAK inhibitors

entry	Compound	JAK3 IC_{50} [nM]	Selectivity over JAK1/JAK2	Selectivity over TYK2	Kinom Selectivity
**1**	**Compound 4 and 5** (Forster *et al.*)	0.127	410 / 2724 fold<br>412 / 1747 fold<br>respectively	3614 fold<br>5820 fold<br>respectively	**410 kinases tested**<br>**@ 100 nM**<br>**no** off targets<br>**@ 500 nM**<br>11 or 1 off target<br>for **4** or **5**, respectively
**2**	**NIBR3049**<br>Novartis<br>**(Thoma et al., 2011)**	1.5	105 / 94 fold	1400 fold	**40 kinases tested**<br>PKC and GSK3β<br>IC_{50} < 1 µM
**3**	**WYE-151650**<br>Wyeth<br>**(Thoma et al., 2014)**	0.9	36 / 14 fold	35 fold	>100-fold selectivity against a panel of 27 non-JAK kinases (data not shown)
**4**	**CP-690,550**<br>Pfizer<br>**(Davis et al., 2011)**	8	1.5 / 0.8 fold	22 fold	**442 kinases tested**<br>Kd's (< 1 µM)<br>12 nM DCAMKL3<br>460 nM LCK<br>550 nM LTK<br>170 nM PKN1<br>470 nM ROCK1<br>420 nM ROCK2<br>600 nM RSK3<br>549 nM RSK4<br>240 nM SNARK<br>120 nM TNK1
**5**	**Compound 3q**<br>Roche<br>**(Soth et al., 2013)**	0.3	12 /3 fold	n.d.	**48 kinases tested**<br>% inhibition @ 1 µM<br>AMPK 67%<br>AurA 78%<br>FGFR1 63%<br>MARK1 67%
**6**	**Cmpd 2**<br>AbbVie<br>**(Goedken et al., 2015)**	7	1600 / 700 fold	> 7000 fold	**n.d.**
**7**	**Cmpd 3**<br>AbbVie<br>**(Goedken et al., 2015)**	3	2000 / 500 fold	> 7000 fold	**78 kinases tested**<br>**trFRET binding assay**<br>**8 kinases IC_{50} < 10 µM**<br>BTK 1.25 µM<br>FGFR1 3.1 µM<br>GRK5 9.4 µM<br>JAK3 0.002 µM<br>LTK 5.6 µM<br>MAP4K2 7.4 µM<br>RET 7.8 µM<br>STK33 7.1 µM

**8**	**Cmpd 4**<br>**(Goedken et al., 2015)**<br>(patented by Cytopia)	20	> 2500 / 2400 fold	300 fold	**78 kinases tested**<br>**trFRET binding assay**<br>**19 kinases IC_{50} < 1-10 µM**<br>**7 kinases IC_{50} < 1 µM**<br>BTK 0.64 µM<br>FLT1 0.55 µM<br>FYN 0.66 µM<br>JAK3 0.3 µM<br>KDR 0.55 µM<br>SRC 0.98 µM<br>STK16 1.0 µM
**9**	**Cmpd 9**<br>Gray Laboratory<br>**(Tan et al., 2015)**	4.8	190 / 220 fold	> 2000 fold	**456 kinases tested**<br>@ 1µM residual activity against:<br>FLT3 0.6 %<br>TTK 1.4 %<br>BLK 1.6 %<br>TXK 3.4 %<br>FAK 4 %<br>CAMKK2 4.2 %<br>GAK 5.4 %<br>LTK 5.9 %<br>ERBB4 6 %<br>SGK 6.8 %<br>ARK5 9.6 %<br>PDGFRB 9.6 %<br>TNK 9.9 %
**10**	**Cmpd 45**<br>Gray Laboratory<br>**(Tan et al., 2015)**	< 0.5	> 70 / > 100 fold	> 800 fold	**456 kinases tested**<br>@ 100 nM residual activity against:<br>AURKA 2.7 %<br>FLT3 10 %
**11**	**JAK3i**<br>**(Smith et al., 2016)**<br>(patented by Merck)	0.43	3500 / 20 000 fold	> 20 000 fold	**Three Cys909-equivalent-kinases tested**<br>1300 fold over EGFR<br>600 fold over ITK<br>50 fold over BTK
**12**	**Compound 31**<br>**(London et al., 2014)**	49	> 200 fold	> 200 fold	**Nine Cys909-equivalent kinases tested:**<br>BLK 0.022 µM<br>BMX 2.41 µM<br>BTK 1.64 µM<br>EGFR > 10 µM<br>ERB-B2 2.05 µM<br>ERB-B4 0.044 µM<br>ITK 0.221 µM<br>TEC 6.93 µM<br>TXK 1.67 µM

Supplemental Experimental Procedures

JAK3 ELISA

Initial JAK3 IC_{50} determination of the synthesized inhibitors was performed as described previously by (Bauer et al., 2014). In this ELISA-based kinase activity assay, a 96-well assay plate is coated with an artificial polypeptide serving as kinase substrate that contains tyrosine residues being phosphorylated by JAK3 in presence of ATP. For the kinase reaction, the recombinant active human JAK3 protein fragment (aa 781 to 1124) is incubated with different concentrations of the inhibitor candidate and an appropriate amount of ATP (twice the Km value). As a consequence, the polypeptidic kinase substrate is phosphorylated as a function of inhibitory potency of the test compound. Phosphorylated tyrosine residues are detected via a monoclonal phopho-tyrosine HRP- conjugated antibody and quantification of phosphorylation degree is perfomed by comparing the phosphorylation achieved in absence (positive control) and presence of inhibitor candidate.

$CD4^+$ T Cell Experiments

$CD4^+$ T cells were purified from peripheral blood mononuclear cells from human donors by magnetic cell separation technology (Miltenyi Biotec), activated with plate-bound anti-CD3 (clone UCHT1) and anti-CD28 (clone CD28.2) antibodies (each 5 µg/ml, Biolegend) in X-VIVO 15 medium (Lonza) for 3 days and expanded with rhIL-2 for another 6 days. After expansion T cells were washed and rested in fresh medium overnight. Equal numbers of T cells were incubated with the indicated concentrations of the JAK inhibitors **1**, **4** or **5** in DMSO or DMSO alone (control) for 1 hour and then stimulated with either rhIL-2 (50 ng/ml; Proleukin, Novartis), rhIL-6 (50 ng/ml; Peprotech), rhIL-4 (50 ng/ml, Peprotech) or IFN-α (1000 U/ml, Roche) for 30 min. After stimulation, cells were lysed in Triton X-100 lysis buffer containing protease and phosphatase inhibitors. Equal amounts of total protein were separated by PAGE, transferred to PVDF membrane, and blotted with Abs recognizing actin (Merck Millipore), specific phospho-STAT antibodies (anti-phospho-Stat5 Tyr694, anti-phospho-Stat3 Tyr705, both Cell Signaling) and IRDye-labelled secondary antibodies (680RD, 800CW, both LI-COR Biosciences) for detection. Specific bands were visualized using an Odyssey infrared imaging system (LI-COR Biosciences).

Protein production, crystallization and structure determination.

Recombinant triple-mutated JAK3 (aa 812-1103, D949A/C1040S/C1048S) containing an N-terminal TEV-cleavable His-tag was expressed in Sf9 cells. The cells were harvested after 48-

52 hours after postinfection with 10 ml/L virus, and were resuspended in 50 mM Tris, pH 8.0, 500 mM NaCl, 5% glycerol, 1 mM TCEP and 5 mM imidazole. Cells were lysed by sonication, and the protein was initially purified by Ni-affinity chromatography. The eluted protein was incubated overnight at 4 ˚C with the compounds at 3-fold molar excess in a presence of TEV protease and an addition of 1 mM TCEP. The cleaved protein was subjected to a reverse Ni-affinity purification, and was purified further by size exclusion chromatography in a buffer containing 20 mM Tris, pH 8.0, 250 mM NaCl, 10 mM DTT and 10% glycerol. The protein was concentrated to 15 mg/ml, and N-phenylurea was added to 0.26%. Mass spectrometry analyses showed that there was a mixture of unmodified JAK3 and covalent adduct of JAK3-inhibitor complex, and this could likely be due to high concentration of reducing agent throughout the purification. JAK3-inhibitor complex at 11-13 mg/ml was crystallized using sitting drop vapour diffusion at 4 ˚C and the condition containing 18-25% PEG 3350, 0.1-0.2 M $MgCl_2$ and 0.1 M MES, pH 5.5-6.1. Crystals were cryoprotected in the reservoir solution supplemented with 22% ethylene glycol. Diffraction data collected at Diamond Light Source were processed with MOSFLM (Powell et al., 2013) and subsequently scaled with Scala (Evans, 2006). Structures were determined using Phaser (McCoy et al., 2007) and the published coordinates of JAK3 (Goedken et al., 2015). Model building alternated with structure refinement was performed in COOT (Emsley et al., 2010) and Refmac (Murshudov et al., 2011), respectively. The final models were verified for their geometric correctness with MOLPROBITY (Chen et al., 2010). The data collection and refinement statistics are summarized in Supplementary Table S4.

Proteros Reporter Displacement Assay

The assay is based on the displacement of a reporter probe by a test compound. Close proximity of the probe and the targeted kinase result in emission of an optical signal. Binding of a competitive test compound to the kinase (displacement of the reporter probe) results in a diminished assay signal. The displacement of the reporter probe is measured over time for eighteen different compound concentrations in two-fold dilution steps starting from 9.8 µM. The measured signal for the lowest compound concentration represents full probe binding while the signal of highest compound concentration represents full probe displacement. For K_d determination the calculated percentages of probe displacement are plotted against compound concentration for the last time point, at which the system reached equilibrium. An IC_{50}-like value, corresponding to 50% probe displacement, is determined by standard fitting

algorithms. According to the Cheng-Prusoff-equation the K_d value is calculated as $K_d = 0.5 \times IC50$.

To determine the kinetic constants (k_{on}, k_{off} and residence time τ) reporter displacement was plotted against time for each concentration and fitted to a mono-exponential decay equation (probe binding = B + A x exp(-k_{obs} x t)). The exponential coefficients represent the apparent association rate k_{obs}. These association rates were plotted against the compound concentration in a secondary plot. Following the equation $k_{obs} = k_{off} + k_{on} \times [cmpd]$ the kon values were determined as the slope of the curve after linear fitting. Off rates are then calculated as $k_{off} = K_d \times k_{on}$ and residence time as $\tau = 1/k_{off}$. For these secondary plots only data was used from compound concentrations at which the apparent association rate k_{obs} could clearly be determined. Examplpes and more details on this method is provided in the reference by (Neumann et al., 2011).

Cell transfection, treatments, and BRET measurements in living cells

The BRET methodology relies on the emission of an optical signal dependent of the spatial proximity of the luciferase-conjugated target protein and a fluorescent-labelled tracer molecule. The displacement of the tracer by a competitive inhibitor therefore diminishes the apparent BRET signal (Machleidt et al., 2015).

Full-length JAK3 and BTK ORFs were subloned into pFC-32K Nluc-CMV Neo (Promega) to generate a C-terminal placement of NanoLuc and BLK and TEC kinase ORFs were subcloned into pFN-31K Nluc-CMV Neo (Promega) to generate an N-terminal placement of NanoLuc. To lower intracellular expression levels of the reporter fusion, each NanoLuc/kinase fusion construct was diluted into Transfection Carrier DNA (Promega) at a mass ratio of 1:10 (mass/mass), prior to forming FuGENE HD complexes according to the manufacturer's protocol (Promega). DNA:FuGENE complexes were formed at a ratio of 1:3 (µg DNA/µL FuGENE). 1 part of the transfection complexes was then mixed with 20 parts (v/v) of HeLa cells (ATCC) suspended at a density of 2×10^5 /mL in DMEM (Gibco) + 10% FBS (GE Healthcare), seeded into T75 flasks and allowed to express for 20 h. Cells were then trypsinized, resuspended in Opti-MEM without phenol red (Life Technologies) and reseeded into white 96-well plates (Corning 4600) at a density of 2×10^4 cells per well. For target engagement, NanoBRET Kinase Tracer-05 (Promega) was added to the cells at 1 µM prior to test compound addition. Serially-diluted test compounds were then added to the cells and allowed to equilibrate for 2 hr at 37°C / 5% CO_2 prior to BRET measurements. For real-time analysis of compound dissociation, the transfected cells were first treated in 15 mL

conical tubes (Corning) with a near-saturating dose (1 □M) of compound for 2 hr in Opti-MEM (Life Technologies). Cells were centrifuged and washed to remove unbound compound prior to immediate addition of NanoBRET Kinase Tracer-05 at 1 □M. To measure BRET in live cells, Intracellular TE Nano-Glo Substrate/Inhibitor (Promega) was added according to the manufacturer's protocol, and filtered luminescence was measured at a single timepoint or in real-time via repeat measurements on a GloMax Discover luminometer equipped with 450 nm BP filter (donor) and 610 nm LP filter (acceptor), using 0.5 s integration time. Milli-BRET units (mBU) are the BRET values x 1000. Competitive displacement data were then graphed with GraphPad Prism software using a 3-parameter curve fit with the following equation:

$$Y = \text{Bottom} + (\text{Top} - \text{Bottom})/(1 + 10^{\wedge}((X - \text{LogIC}_{50})))$$

Further details on tracer based BRET assays included examples of tracer structures are provided in (Robers et al., 2015).

Chemical Synthesis

General: Reagents, starting materials and solvents were of commercial quality and were used without further purification unless otherwise stated. TLC analysis was carried out on Merck 60 F_{254} silica gel plates and visualized under UV light at 254 nm and 365 nm. Preparative column chromatography was carried out on Grace Davison Davisil LC60A 20-45 micron or Merck Geduran Si60 63-200 micron silica using a Interchim PuriFlash 430 automated flash chromatography system. The purity of final compounds was determined via RP-HPLC on a Hewlett Packard 1090 Series II LC with a Phenomenex Luna C8 column (150 x 4.6 mm, 5 µm) and detection was performed by a UV DAD at 254 nm and 230 nm wavelength. Elution was carried out with the following gradient: 0.01 M KH_2PO_4, pH 2.30 (solvent A), MeOH (solvent B), 40 % B to 85 % B in 8 min, 85 % B for 5 min, 85 % to 40 % B in 1 min, 40 % B for 2 min, stop time 16 min, flow 1.5 ml/min. NMR spectra were recorded on a Bruker Avance 200 or Bruker Avance 400 NMR spectrometer. Chemical shifts are reported in ppm relative to TMS and the spectra were calibrated against the residual proton peak of the used deuterated solvent. Standard mass spectra were obtained either as ESI-MS (pos. and/or neg. mode) from a Advion DCMS interface, (settings as follows: ESI voltage 3,50 kV, capillary voltage 187 V, source voltage 44 V, capillary temperature 250 °C, desolvation gas temperature 250 °C, gas flow 5 l/min) or as FAB-MS (pos. and/or neg. mode) measured by the mass spectrometry department, Institute of Organic Chemistry, Eberhard-Karls-University Tuebingen. HRMS for final compounds was performed on an Sciex TripleTof 5600+ mass spectrometer with an Duospray source, coupled to a 1290 UHPLC from Agilent equipped with an PAL-HTS Autosampler from CTC. Preliminary chromatographic separation was performed on a Phenomenex Kinetex C18 2.8µ 100x3mm 100Å coreshell technology column using the following gradient: H_2O with 0.1 % formic acid (solvent A), Acetonitrile with 0.1% formic acid (solvent B) 0-1min 5 % B, 1-10min 5 to 100 % B, 5-15 100 % B. Flow 0.5 ml/min, column temperature 20°C. Mass analysis was run in ESI+ Mode, settings were as follows: curtain gas 30 psi, nebulizer gas 40 psi, drying gas 50 psi, source temperature 400 °C, ion source floating voltage +5500 V. Analysis was run as information dependent acquisition, i.e. every cycle consisted of a TOF survey scan from 100 to 2000 m/z and subsequent fragmentation scans were triggered automatically by acquisition software. To ensure MSMS spectra generation of synthesized compounds, IDA was supplied with a inclusion list containing the respective $[M+H]^+$ m/z ratios. Collision energy was set to 27 V.

Synthetic Procedures

Preparation of Starting Materials and Reagents

Hydroxymethyl benzaldehydes

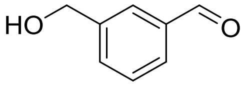

9a

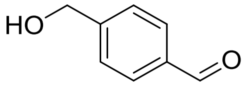

9b

*3-(hydroxymethyl)benzaldehyde (**9a**).* Isophthalic aldehyde (1.0 g, 7.46 mmol) was suspendend in 5 ml THF and 10 ml EtOH and the suspension was cooled with an ice/water bath. $NaBH_4$ granules (100 mg, 2.64 mmol) were added in several portions (dissolves slowly) and the reaction was monitored by TLC (PE/EA 1:1). About 15 min after complete addition starting material was consumed and the reaction was quenched by addition of 5 ml HCl_{aq} (10 %). The mixture was diluted with water and extracted with EA (3x20 ml). The combined extracts were washed two times with brine, dried over Na_2SO_4 and evaporated to yield 925 mg (91 %) of **9a** as yellowish oil. ^{1}H NMR (200 MHz, $CDCl_3$) δ 9.90 (s, 1H), 7.79 (s, 1H), 7.71 (d, J = 7.5 Hz, 1H), 7.57 (d, J = 7.5 Hz, 1H), 7.44 (t, J = 7.5 Hz, 1H), 4.69 (s, 2H), 3.30 (s, 1H). ^{13}C NMR (50 MHz, $CDCl_3$) δ 192.7, 142.2, 136.4, 133.0, 129.2, 129.0, 127.8, 64.1

*4-(hydroxymethyl)benzaldehyde (**9b**)* was prepared from terephthalic aldehyde following the same procedure as for **9a**, but afforded flash purification (PE/EA 20-80 %). Yield 653 mg (64 %) of **9b** as white solid. ^{1}H NMR (200 MHz, $CDCl_3$) δ 9.85 (s, 1H), 7.80 (d, J = 7.9 Hz, 2H), 7.47 (d, J = 7.8 Hz, 2H), 4.83 (s, 2H), 2.39 (s, 1H). ^{13}C NMR (50 MHz, $CDCl_3$) δ 191.9, 147.8, 135.6, 130.0, 127.0, 64.7

Horner-Wadsworth-Emmons-Reagents

HWE-reagents were obtained from the alkyl chlorides under Arbuzov conditions following a protocol of *(Zhang et al., 2009).*

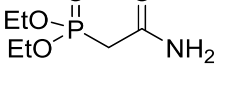

10a

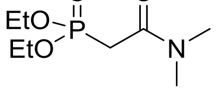

10b

*diethyl (2-amino-2-oxoethyl)phosphonate (**10a**).* Chloroacetamide (2.81 g, 30 mmol) and 15 ml triethyl phosphite were combined in a 25 ml round bottomed flask und heated to 150 °C oil bath temperature overnight. The excess triethyl phosphite was removed under reduced pressure and the residue was poured in 100 ml cold Et_2O. The suspension was stirred for

5 min, then the precipitate was collected by filtration, washed with Et_2O and dried in vacuo. 3.67 g (63 %) of the product **10a** was obtained as pale yellowish crystalline solid. ^1H NMR (200 MHz, DMSO) δ 7.38 (br s, 1H), 7.03 (br s, 1H), 4.02 (dq, *J* = 14.1, 7.1 Hz, 4H), 2.80 (d, *J* = 21.4 Hz, 2H), 1.22 (t, *J* = 7.1 Hz, 6H) ^{13}C NMR (50 MHz, DMSO) δ 166.0 (d, J = 5.5 Hz), 61.6 (d, *J* = 6.1 Hz), 34.5 (d, *J* = 131.3 Hz), 16.2 (d, *J* = 6.1 Hz)

2-chloro-N,N-dimethylacetamide was prepared via Schotten-Baumann procedure reported from Kem K *et al.(Kem et al., 1981)* A biphasic mixture of 50 ml DCM and 50 ml $NaOH_{aq}$ (20 %wt) was cooled to -10 °C internal temperature. Dimethylamine-HCl (4.89 g, 60 mmol) was added in one portion, followed by the dropwise addition of chloroacetyl chloride (5.65 g, 50 mmol) to the vigorously stirred mixture in a rate that the temperature kept below 0 °C. After complete addition, stirring was continued for 20 min. Then the phases were separated and the aqueous phase was extracted with DCM (3x20 ml). The combined organic phases were washed with HCl (10 %) and brine. After drying over Na_2SO_4 and evaporation of the solvent 4,52 g (72 %) of the crude dimethylacetamide was obtained as brownish oil. The product was used in the next step without further purification. ^1H NMR (200 MHz, $CDCl_3$) δ 4.05 (s, 2H), 3.06 (s, 3H), 2.94 (s, 3H). ^{13}C NMR (50 MHz, CDCl3) δ 166.5, 41.2, 37.6, 36.0

diethyl (2-(dimethylamino)-2-oxoethyl)phosphonate ***(10b)*** The crude 2-chloro-*N,N*-dimethylacetamide (4.39 g, 36 mmol) from the previous step and 7.2 ml triethyl phosphite were combined in a 25 ml round bottomed flask und heated to 150 °C oil bath temperature overnight. The mixture was subjected to a short-path vacuum distillation and the first 0.5-1.0 ml were discarded to remove excess triethyl phosphite. The main fraction was collected from 125-135 °C at 0.25 mbar yielding 7.38 g (92 %) of **10b** as colorless oil. ^1H NMR (200 MHz, $CDCl_3$) δ 4.12 (dq, *J* = 14.4, 7.0 Hz, 4H), 3.12 – 2.88 (m, 8H), 1.28 (td, *J* = 7.0, 0.5 Hz, 6H). ^{13}C NMR (50 MHz, $CDCl_3$) δ 164.9 (d, *J* = 5.4 Hz), 62.6 (d, *J* = 6.5 Hz), 38.6, 35.8, 33.4 (d, *J* = 133.5 Hz), 16.4 (d, *J* = 6.4 Hz).

Preparation of 1,6-dihydroimidazo[4,5-d]pyrrolo[2,3-b]pyridine intermediates and 2

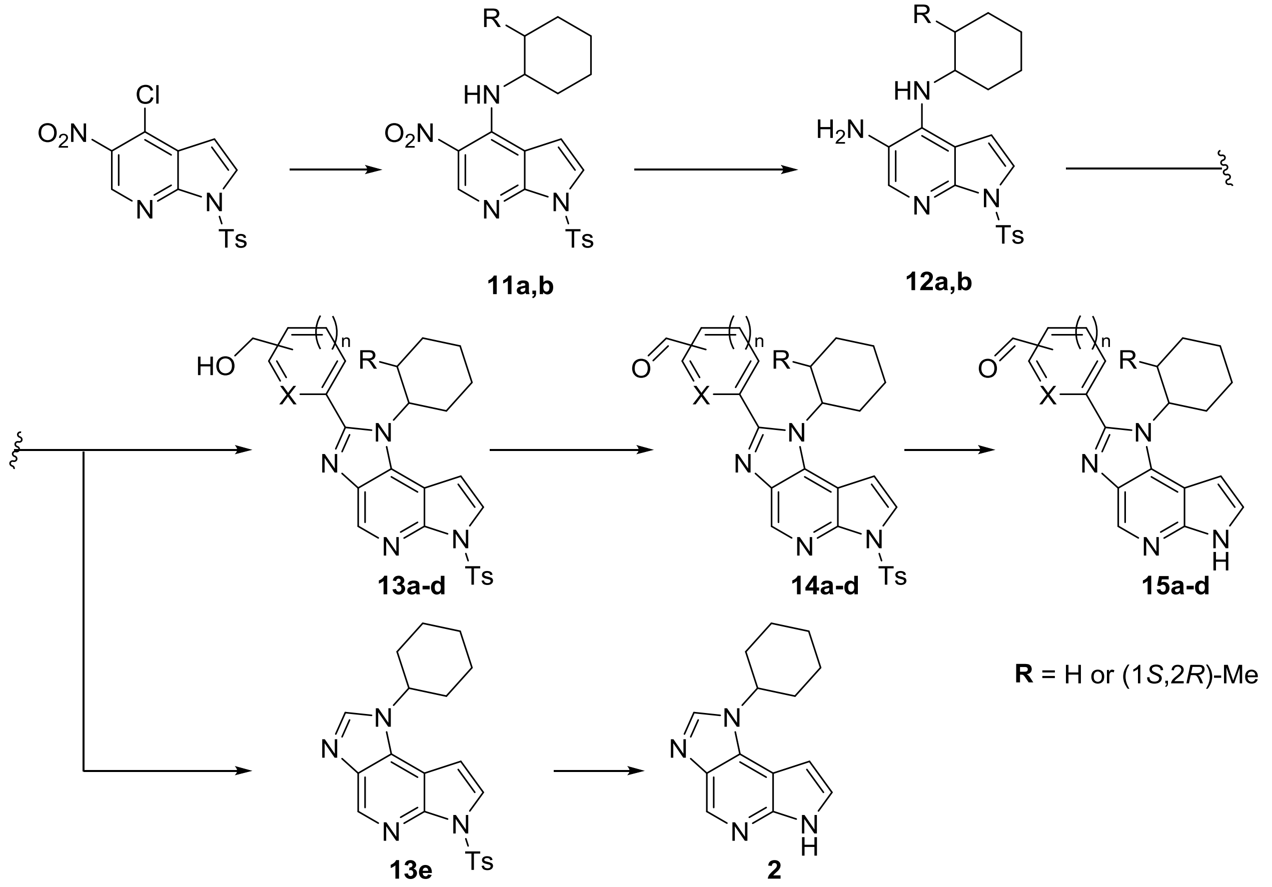

4-chloro-5-nitro-1-tosyl-1*H*-pyrrolo[2,3-*b*]pyridine and (1*S*,2*R*)-2-methylcyclohexan-1-amine hydrochloride were synthesized according to known procedures (Gehringer et al., 2014; Knupp and Frahm, 1984).

Nucleophilic aromatic substitution of cyclohexyl-side chains

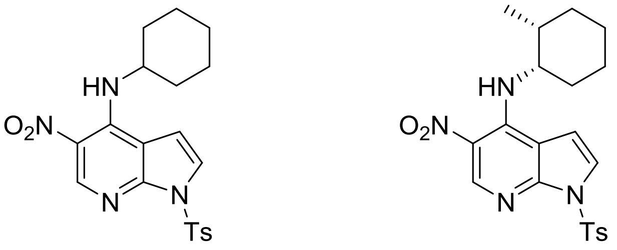

11a **11b**

*N-cyclohexyl-5-nitro-1-tosyl-1H-pyrrolo[2,3-b]pyridin-4-amine (**11a**).* To a stirred suspension of 4-chloro-5-nitro-1-tosyl-1*H*-pyrrolo[2,3-*b*]pyridine(Gehringer et al., 2014) (1407 mg, 4 mmol) in *i*PrOH (16 ml) was added cyclohexylamine (1190 mg, 12 mmol). The mixture was heated to reflux for 90 min and was then poured on sat. NH_4Cl / ice. The precipitate was filtered off, washed with water and dried in vacou, yielding 1537 mg (93 %) of **11a** as yellow solid which was carried on to the next step without further purification. ^1H NMR (200 MHz, $CDCl_3$) δ 9.20 – 8.95 (m, 2H), 8.05 (d, J = 8.4 Hz, 2H), 7.57 (d, J = 4.1 Hz, 1H), 7.29 (d, J =

8.4 Hz, 2H), 6.69 (d, J = 4.1 Hz, 1H), 3.94 (m, 1H), 2.38 (s, 3H), 2.19 – 1.99 (m, 2H), 1.93 – 1.58 (m, 3H), 1.57 – 1.31 (m, 5H) ^{13}C NMR (50 MHz, $CDCl_3$) δ 146.41, 145.9, 144.9, 134.9, 129.8, 128.6, 126.6, 123.6, 107.6, 106.7, 52.9, 33.5, 25.4, 24.4, 21.8 DC-MS (ESI) m/z: 415.4 $[M+H]^+$ HPLC t_{ret} = 9.439 min

*N-((1S,2R)-2-methylcyclohexyl)-5-nitro-1-tosyl-1H-pyrrolo[2,3-b]pyridin-4-amine (**11b**).* Following the same procedure as with **11a** but using (1*S*,2*R*)-2-methylcyclohexan-1-amine hydrochloride(Knupp and Frahm, 1984) (1.1 equiv) instead of cyclohexylamine and DIPEA as additional base (2.3 equiv). Same precipitation workup afforded **11b** as yellow solid. Yield: 83 % at 3 mmol scale. ^{1}H NMR (200 MHz, $CDCl_3$) δ 9.31 (br d, J = 8.3 Hz, 1H), 9.10 (s, 1H), 8.06 (d, J = 8.1 Hz, 2H), 7.57 (d, J = 4.1 Hz, 1H), 7.30 (d, J = 8.1 Hz, 2H), 6.72 (d, J = 4.1 Hz, 1H), 4.26 – 4.08 (m, 1H), 2.39 (s, 3H), 2.09 – 1.83 (m, 2H), 1.78 – 1.59 (m, 3H), 1.57 – 1.32 (m, 4H), 0.96 (d, J = 6.9 Hz, 3H). ^{13}C NMR (50 MHz, $CDCl_3$) δ 148.9, 146.5, 145.9, 145.6, 134.9, 129.8, 128.6, 126.8, 123.5, 107.7, 106.8, 54.9, 34.4, 30.2, 29.5, 23.7, 21.8, 21.6, 17.1 DC-MS (ESI) m/z: 451.2 $[M+Na]^+$ HPLC t_{ret} = 9.442 min

Reduction to vicinal aryl diamines

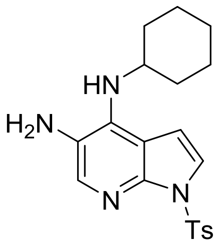

12a

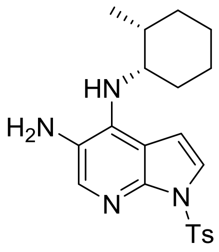

12b

*N^4-cyclohexyl-1-tosyl-1H-pyrrolo[2,3-b]pyridine-4,5-diamine (**12a**).* The crude **11a** (1500 mg, 3.6 mmol) was dissolved in MeOH / EtOAc (1:4, ca. 80 ml) and the solution was transferred to a glass-lined hydrogenation reactor. Raney Nickel (ca. 0,3 g as suspension in water, washed with MeOH) was added and hydrogen was bubbled through the magnetically stirred mixture. The reactor was purged with hydrogen, sealed and the pressure was adjusted to 0,5 MPa. The reactor was placed in a water bath at 45 °C and stirring was continued overnight. At this point TLC indicated full conversion. The catalyst was filtered off over celite and was washed several times with MeOH and EtOAc. The filtrate was evaporated to yield 1380 mg (99 %) of **12a** as greyish to purple foam. ^{1}H NMR (200 MHz, $CDCl_3$) δ 7.99 (d, J = 8.1 Hz, 2H), 7.82 (s, 1H), 7.44 (d, J = 4.1 Hz, 1H), 7.21 (d, J = 8.1 Hz, 2H), 6.53 (d, J = 4.1 Hz, 1H), 4.75 (br s, 1H), 3.77 – 3.57 (m, 1H), 2.96 (br s, 2H), 2.33 (s, 3H), 2.11 – 1.95 (m, 2H), 1.86 – 1.57 (m, 3H), 1.45 – 1.10 (m, 5H) ^{13}C NMR (50 MHz, $CDCl_3$) δ 145.8, 144.8,

141.0, 137.1, 135.7, 129.6, 128.0, 123.1, 122.3, 108.0, 105.0, 52.5, 34.4, 25.7, 2.9, 21.7 DC-MS (ESI) m/z: 385.4 $[M+H]^+$ HPLC t_{ret} = 5.895 min

*N^4-((1S,2R)-2-methylcyclohexyl)-1-tosyl-1H-pyrrolo[2,3-b]pyridine-4,5-diamine **(12b)*** was obtained from crude **11b** following the same procedure as with **12a**. Yield: 99 % at 3 mmol scale, obtained as purple foam. ^{1}H NMR (400 MHz, $CDCl_3$) δ 8.00 (d, *J* = 8.2 Hz, 2H), 7.83 (s, 1H), 7.43 (d, *J* = 4.1 Hz, 1H), 7.21 (d, *J* = 8.2 Hz, 2H), 6.55 (d, *J* = 4.2 Hz, 1H), 5.05 (bs, 1H), 3.99 – 3.86 (m, 1H), 2.92 (bs, 2H), 2.33 (s, 3H), 2.03 – 1.94 (m, 1H), 1.76 – 1.67 (m, 1H), 1.64 – 1.49 (m, 4H), 1.47 – 1.31 (m, 3H), 0.91 (d, *J* = 7.0 Hz, 3H). ^{13}C NMR (100 MHz, $CDCl_3$) δ 146.2, 144.7, 141.8, 137.9, 135.9, 129.5, 128.1, 123.0, 122.3, 108.2, 105.0, 54.0, 34.0, 30.1, 30.0, 23.0, 22.6, 21.7, 15.8 DC-MS (ESI) m/z: 399.2 $[M+H]^+$ HPLC t_{ret} = 5.986 min

These aryl diamines seem to be sensitive to air oxygen, since the product solution darkens rapidly upon air contact during filtration and the yields of subsequent reactions decrease if the diamine is stored over longer time periods at ambient temperature.

Imidazole ring closure reactions

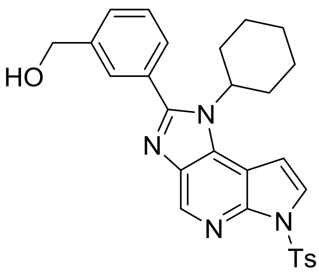

13a

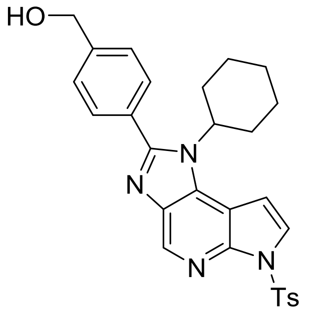

13b

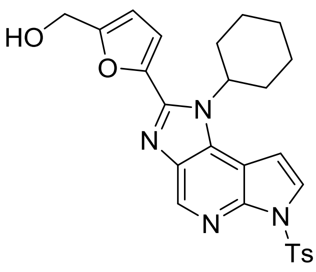

13c

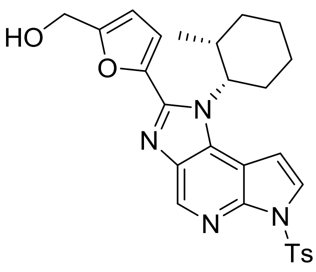

13d

Aryl substituted imidazole closure reactions were conducted according to a procedure reported by *(Beaulieu et al., 2003).*

*(5-(1-cyclohexyl-6-tosyl-1,6-dihydroimidazo[4,5-d]pyrrolo[2,3-b]pyridin-2-yl)furan-2-yl)methanol (**13c**).* A solution of 5-hydroxymethyl-2-furaldehyde (151 mg, 1.2 mmol) and **12a** (384 mg, 1.0 mmol) in DMF (10 ml) and water (350 µl) was stirred for about 10 min at ambient temperature before $KHSO_5$ (triple salt) (430 mg, 0.7 mmol) was added as solid in one portion. After stirring one hour at ambient temperature, TLC indicated total consumption of starting material. The mixture was poured on half-saturated $NaHCO_3$ solution, the precipitate was filtered off and was taken up in DCM. The organic phase was washed with brine once, dried over Na_2SO_4 and evaporated under reduced pressure. The residue was purified by flash

chromatography using gradient elution (petrol ether / (EtOAc+5% MeOH) 50 – 100%) to yield 253 mg (52 %) of **13c** as brownish foam. ^{1}H NMR (200 MHz, $CDCl_3$) δ 8.89 (s, 1H), 8.10 (d, J = 8.4 Hz, 2H), 7.83 (d, J = 4.1 Hz, 1H), 7.25 (d, J = 8.4 Hz, 2H), 6.92 (d, J = 4.1 Hz, 1H), 6.89 (d, J = 3.4 Hz, 1H), 6.44 (d, J = 3.4 Hz, 1H), 4.85 – 4.61 (m, 3H), 2.33 (s, 3H), 2.28 – 2.08 (m, 2H), 1.95 – 1.72 (m, 5H), 1.45 – 1.27 (m, 3H) ^{13}C NMR (50 MHz, $CDCl_3$) δ 156.9, 145.3, 144.4, 143.6, 142.9, 138.3, 136.9, 135.4, 133.2, 129.7, 128.4, 124.8, 114.5, 109.8, 107.8, 104.7, 57.4, 57.3, 30.8, 25.8, 24.9, 21.7 DC-MS (ESI) m/z: 491.3 $[M+H]^+$ HPLC t_{ret} = 8.112 min

*(3-(1-cyclohexyl-6-tosyl-1,6-dihydroimidazo[4,5-d]pyrrolo[2,3-b]pyridin-2-yl)phenyl)methanol (**13a**)* was obtained from 264 mg **12a** and 112 mg **9a** following the same procedure as described for **13c** with a reaction time of one hour at ambient temperature. Crude product afforded flash purification using gradient elution (petrol ether / (EtOAc+5% MeOH) 30 – 100%) to yield 247 mg (72 %) of **13a** as brownish foam. ^{1}H NMR (200 MHz, $CDCl_3$) δ 8.93 (s, 1H), 8.11 (d, J = 8.3 Hz, 2H), 7.85 (d, J = 4.1 Hz, 1H), 7.62 (s, 1H), 7.53 – 7.39 (m, 3H), 7.26 (d, J = 8.3 Hz, 3H), 6.97 (d, J = 4.1 Hz, 1H), 4.75 (s, 2H), 4.56 – 4.30 (m, 1H), 2.95 (bs, 1H), 2.40 – 2.12 (m, 5H), 2.04 – 1.72 (m, 5H), 1.46 – 1.29 (m, 3H). ^{13}C NMR (50 MHz, $CDCl_3$) δ 153.9, 145.3, 142.9, 142.5, 138.2, 136.6, 135.4, 132.9, 130.3, 129.7, 128.9, 128.7, 128.4, 128.3, 128.1, 124.9, 107.8, 105.0, 64.5, 56.9, 31.1, 25.6, 24.9, 21.8 FAB-MS m/z: 501.2 $[M+H]^+$ HPLC t_{ret} = 8.333 min

*(4-(1-cyclohexyl-6-tosyl-1,6-dihydroimidazo[4,5-d]pyrrolo[2,3-b]pyridin-2-yl)phenyl)methanol (**13b**)* was obtained from 384 mg **12a** and 164 mg **9b** following the above described procedure for **13c** with a reaction time of two hours at ambient temperature. Crude product afforded flash purification using gradient elution (petrol ether / (EtOAc+5% MeOH) 50 – 100%) to yield 343 mg (69 %) of **13b** as brownish foam. ^{1}H NMR (200 MHz, $CDCl_3$) δ 8.94 (s, 1H), 8.10 (d, J = 8.4 Hz, 2H), 7.83 (d, J = 4.1 Hz, 1H), 7.44 (q, J = 8.5 Hz, 4H), 7.25 (d, J = 8.4 Hz, 2H), 6.97 (d, J = 4.1 Hz, 1H), 4.77 (s, 2H), 4.40 (tt, J = 12.1, 3.8 Hz, 1H), 3.76 (br s, 1H), 2.40 – 2.14 (m, 5H), 2.00 – 1.71 (m, 5H), 1.47 – 1.28 (m, 3H). ^{13}C NMR (50 MHz, $CDCl_3$) δ 154.0, 145.3, 144.0, 142.9, 138.3, 137.0, 135.4, 133.0, 129.7, 129.5, 129.1, 128.4, 126.9, 124.7, 107.8, 105.1, 64.2, 56.8, 31.1, 25.6, 24.9, 21.7 DC-MS (ESI) m/z: 522.9 $[M+Na]^+$ HPLC t_{ret} = 8.077 min

*(5-(1-((1S,2R)-2-methylcyclohexyl)-6-tosyl-1,6-dihydroimidazo[4,5-d]pyrrolo[2,3-b]pyridin-2-yl)furan-2-yl)methanol (**13d**)* was obtained from 400 mg **12b** and 152 mg 5-hydroxymethyl-2-furaldehyde following the above described procedure for **13c**, but reaction was run

overnight at ambient temperature. Crude product afforded flash purification using gradient elution (hexane / (EtOAc+10% *i*PrOH) 50 – 100%) to yield 162 mg (32 %) of **13d** as brownish foam. ^{1}H NMR (400 MHz, $CDCl_3$) δ 8.88 (s, 1H), 8.10 (d, *J* = 8.1 Hz, 2H), 7.79 (d, *J* = 3.8 Hz, 1H), 7.25 (d, *J* = 8.1 Hz, 2H), 6.87 – 6.75 (m, 2H), 6.40 (d, *J* = 2.9 Hz, 1H), 4.81 – 4.72 (m, 1H), 4.68 (s, 2H), 2.53 – 2.42 (m, 1H), 2.33 (s, 3H), 2.14 (s, 1H), 1.86 (d, *J* = 12.3 Hz, 1H), 1.70 (t, *J* = 18.6 Hz, 3H), 1.58 – 1.27 (m, 4H), 0.87 (d, *J* = 7.0 Hz, 3H). ^{13}C NMR (100 MHz, $CDCl_3$) δ 156.5, 145.3, 144.7, 143.7, 143.2, 138.4, 136.2, 135.5, 134.5, 129.7, 128.4, 124.8, 115.1, 109.3, 107.8, 103.6, 62.5, 57.4, 34.3, 32.0, 27.0, 25.4, 21.7, 19.5, 13.0 DC-MS (ESI) m/z: 505.3 $[M+H]^+$ HPLC t_{ret} = 7.955 min

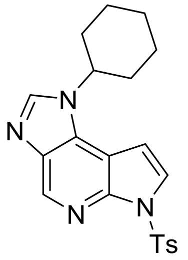

13e

1-cyclohexyl-6-tosyl-1,6-dihydroimidazo[4,5-d]pyrrolo[2,3-b]pyridine (13e). **12a** (38 mg, 0.1 mmol), triethyl orthoformate (45 mg, 0.3 mmol) and TsOH x H_2O (2 mg, 0.01 mmol) were dissolved in 1 ml toluene and heated to 70 °C heating block temperature for two hours. TLC indicated full consumption of starting material and the mixture was poured on sat. $NaHCO_{3(aq)}$. The biphasic mixture was extracted with EtOAc (5x10 ml) and the combined extracts were dried over Na_2SO_4 and evaporated to dryness. The residue was subjected to flash purification using gradient elution (petrol ether / (EtOAc+5% MeOH) 50 – 100%) to yield 29 mg (75 %) of **13e** as brownish solid. ^{1}H NMR (400 MHz, $CDCl_3$) δ 8.89 (s, 1H), 8.07 (d, *J* = 8.2 Hz, 2H), 8.02 (s, 1H), 7.77 (d, *J* = 3.9 Hz, 1H), 7.22 (d, *J* = 8.2 Hz, 2H), 6.74 (d, *J* = 3.9 Hz, 1H), 4.44 – 4.30 (m, 1H), 2.31 (s, 3H), 2.29 – 2.18 (m, 2H), 2.06 – 1.93 (m, 2H), 1.87 – 1.67 (m, 3H), 1.61 – 1.45 (m, 2H), 1.39 – 1.22 (m, 1H). ^{13}C NMR (100 MHz, $CDCl_3$) δ 145.2, 142.9, 139.9, 138.5, 137.2, 135.5, 132.5, 129.6, 128.3, 125.2, 107.5, 101.7, 57.1, 33.5, 25.7, 25.3, 21.7 DC-MS (ESI) m/z: 417.2 $[M+Na]^+$ HPLC t_{ret} = 7.427 min

Preparation of tosylated aryl aldehydes (14a-d)

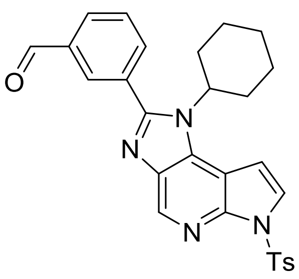
14a

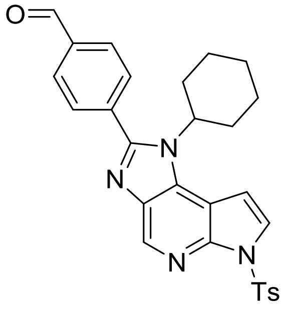
14b

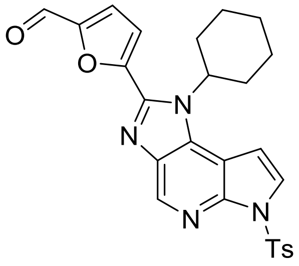
14c

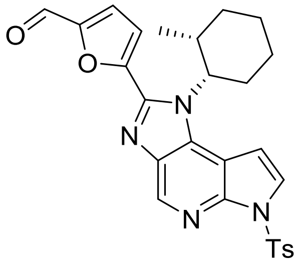
14d

Oxidation of benzylic alcohols **13a-d** was performed using Dess-Martin-Periodinane, which was prepared from 2-iodo-benzoic acid following the procedures of (Frigerio et al., 1999) and (Ireland and Liu, 1993).

*5-(1-cyclohexyl-6-tosyl-1,6-dihydroimidazo[4,5-d]pyrrolo[2,3-b]pyridin-2-yl)furan-2-carbaldehyde (**14c**).* To an ice-cooled solution of **13c** (250 mg, 0.51 mmol) in dry DCM (11 ml) was added Dess-Martin-Periodinane (259 mg, 0.61 mmol) as solid in one portion. The stirred reaction mixture was allowed to reach ambient temperature slowly and after one hour TLC indicated full conversion. The reaction was quenched with sat. $NaHCO_3$ and the biphasic mixture was extracted with DCM (5 x 20 ml). The combined organic extracts were dried over Na_2SO_4, evaporated and the residue purified by flash chromatography (gradient elution DCM / MeOH 1-5%) to yield 200 mg (80 %) of **14c** as brownish foam. ^{1}H NMR (200 MHz, $CDCl_3$) δ 9.77 (s, 1H), 8.93 (s, 1H), 8.12 (d, J = 8.5 Hz, 2H), 7.86 (d, J = 4.1 Hz, 1H), 7.41 (d, J = 3.7 Hz, 1H), 7.32 – 7.22 (m, 3H), 6.98 (d, J = 4.1 Hz, 1H), 5.10 – 4.88 (m, 1H), 2.43 – 2.20 (m, 5H), 2.11 – 1.84 (m, 5H), 1.63 – 1.43 (m, 3H) ^{13}C NMR (50 MHz, $CDCl_3$) δ 177.4, 153.2, 149.7, 145.4, 143.2, 142.6, 139.0, 137.4, 135.4, 133.8, 129.8, 128.4, 125.0, 122.0, 115.4, 107.8, 104.6, 57.9, 31.0, 26.0, 25.0, 21.8 DC-MS (ESI) m/z: 511.3 $[M+Na]^+$ HPLC t_{ret} = 8.520 min

*3-(1-cyclohexyl-6-tosyl-1,6-dihydroimidazo[4,5-d]pyrrolo[2,3-b]pyridin-2-yl)benzaldehyde (**14a**)* was obtained from 240 mg **13a** following the above described procedure for **14c** with a reaction time of one hour. Crude product afforded flash purification using gradient elution (petrol ether / (EtOAc+5% MeOH) 30 – 80%) to yield 220 mg (92 %) of **14a** as brownish foam. ^{1}H NMR (200 MHz, $CDCl_3$) δ 8.93 (s, 1H), 8.11 (d, J = 8.3 Hz, 2H), 7.85 (d, J = 4.1 Hz, 1H), 7.62 (s, 1H), 7.53 – 7.39 (m, 3H), 7.26 (d, J = 8.3 Hz, 3H), 6.97 (d, J = 4.1 Hz, 1H), 4.75 (s, 2H), 4.56 – 4.30 (m, 1H), 2.95 (bs, 1H), 2.40 – 2.12 (m, 5H), 2.04 – 1.72 (m, 5H), 1.46 – 1.29 (m, 3H). ^{13}C NMR (50 MHz, $CDCl_3$) δ 153.9, 145.3, 142.9, 142.5, 138.2, 136.6,

135.4, 132.9, 130.3, 129.7, 128.9, 128.7, 128.4, 128.3, 128.1, 124.9, 107.8, 105.0, 64.5, 56.9, 31.1, 25.6, 24.9, 21.8 FAB-MS m/z: 499.2 $[M+H]^+$ HPLC t_{ret} = 8.751 min

*4-(1-cyclohexyl-6-tosyl-1,6-dihydroimidazo[4,5-d]pyrrolo[2,3-b]pyridin-2-yl)benzaldehyde (**14b**)* was obtained from 148 mg **13b** following the above described procedure for **14c** with a reaction time of three hours. Crude product afforded flash purification using gradient elution (petrol ether / (EtOAc+5% MeOH) 30 – 80%) to yield 118 mg (80 %) of **14b** as brownish foam. ^{1}H NMR (200 MHz, $CDCl_3$) δ 10.10 (s, 1H), 8.94 (s, 1H), 8.17 – 7.95 (m, 4H), 7.84 (d, *J* = 4.0 Hz, 1H), 7.79 (d, *J* = 7.8 Hz, 2H), 7.24 (d, *J* = 7.8 Hz, 2H), 6.97 (d, *J* = 4.0 Hz, 1H), 4.53 – 4.28 (m, 1H), 2.41 – 2.17 (m, 5H), 2.05 – 1.75 (m, 5H), 1.55 – 1.28 (m, 3H). ^{13}C NMR (50 MHz, $CDCl_3$) δ 191.6, 152.5, 145.3, 142.9, 138.8, 137.3, 137.2 136.4, 135.3, 133.2, 130.3, 130.0, 129.7, 128.3, 124.9, 107.8, 104.9, 57.1, 31.1, 25.6, 24.8, 21.7 DC-MS (ESI) m/z: 520.9 $[M+H]^+$ HPLC t_{ret} = 8.347 min

*5-(1-((1S,2R)-2-methylcyclohexyl)-6-tosyl-1,6-dihydroimidazo[4,5-d]pyrrolo[2,3-b]pyridin-2-yl)furan-2-carbaldehyde (**14d**)* was obtained from 56 mg **13d** following the above described procedure for **14c** with a reaction time of 30 minutes. Crude product afforded flash purification using gradient elution (petrol ether / (hexane / (EtOAc+10% *i*PrOH) 50 – 100%) to yield 49 mg (88 %) of **14d** as off-white foam. ^{1}H NMR (400 MHz, $CDCl_3$) δ 9.75 (s, 1H), 8.93 (s, 1H), 8.11 (d, *J* = 8.3 Hz, 2H), 7.84 (d, *J* = 4.0 Hz, 1H), 7.39 (d, *J* = 3.6 Hz, 1H), 7.27 (d, *J* = 8.3 Hz, 2H), 7.20 (d, *J* = 3.6 Hz, 1H), 6.88 (d, *J* = 4.0 Hz, 1H), 4.96 (dt, *J* = 13.0, 3.7 Hz, 1H), 2.73 – 2.61 (m, 1H), 2.35 (s, 3H), 2.30 – 2.15 (m, 1H), 1.98 – 1.85 (m, 2H), 1.85 – 1.73 (m, 2H), 1.63 – 1.50 (m, 2H), 1.46 – 1.35 (m, 1H), 1.04 (d, *J* = 7.2 Hz, 3H). ^{13}C NMR (100 MHz, $CDCl_3$) δ 177.5, 153.0, 149.8, 145.4, 143.4, 142.8, 139.0, 136.7, 135.5, 135.0, 129.7, 128.4, 125.0, 121.7, 116.1, 107.9, 103.4, 63.0, 34.5, 32.1, 27.1, 25.6, 21.7, 19.5, 13.0 DC-MS (ESI) m/z: 525.3 $[M+Na]^+$ HPLC t_{ret} = 8.188 min

Preparation of detosylated compounds 2 and 15a-d

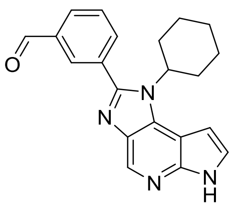
15a

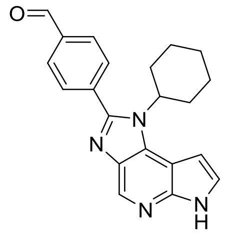
15b

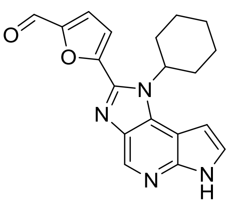
15c

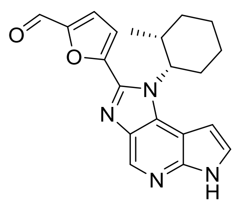
15d

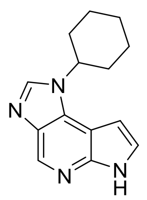
2

5-(1-cyclohexyl-1,6-dihydroimidazo[4,5-d]pyrrolo[2,3-b]pyridin-2-yl)furan-2-carbaldehyde ***(15c)***. To a stirred solution of **14c** (200 mg, 0.41 mmol) in dry THF (9 ml) and dry MeOH (3 ml) was added cesium carbonate (400 mg, 1.23 mmol) at ambient temperature. After four hours at ambient temperature, the reaction was complete according to TLC. The reaction was quenched with sat. NH_4Cl, followed by extraction with EtOAc (5 x 15 ml). The combined organic phases were dried over Na_2SO_4, evaporated and the residue purified by flash chromatography (gradient elution DCM / MeOH 4-8%) to yield 69 mg (50 %) of **15c** as yellow solid. ^{1}H NMR (200 MHz, $CDCl_3$) δ 12.05 (br s, 1H), 9.78 (s, 1H), 8.89 (s, 1H), 7.51 (d, J = 3.4 Hz, 1H), 7.42 (d, J = 3.7 Hz, 1H), 7.30 (d, J = 3.7 Hz, 1H), 6.90 (d, J = 3.4 Hz, 1H), 5.17 – 4.94 (m, 1H), 2.63 – 2.36 (m, 2H), 2.18 – 1.79 (m, 5H), 1.69 – 1.43 (m, 3H). ^{13}C NMR (50 MHz, $CDCl_3$) δ 177.5, 153.1, 150.3, 145.1, 141.4, 136.8, 135.7, 134.4, 123.6, 122.0, 114.7, 105.2, 100.9, 57.8, 30.9, 26.0, 25.0 DC-MS (ESI) m/z: 357.2 $[M+Na]^+$ HPLC t_{ret} = 6.991 min

3-(1-cyclohexyl-1,6-dihydroimidazo[4,5-d]pyrrolo[2,3-b]pyridin-2-yl)benzaldehyde ***(15a)*** was obtained from 220 mg **14a** following the above described procedure for **15c** with a reaction time of three hours at ambient temperature. Crude product afforded flash purification using gradient elution (DCM / MeOH 3 – 10%) to yield 120 mg (79 %) of **15a** as beige solid. ^{1}H NMR (200 MHz, $CDCl_3$) δ 11.85 (br s, 1H), 10.13 (s, 1H), 8.91 (s, 1H), 8.21 (s, 1H), 8.07 (d, J = 7.6 Hz, 1H), 7.96 (d, J = 7.6 Hz, 1H), 7.74 (t, J = 7.6 Hz, 1H), 7.52 (d, J = 3.5 Hz, 1H), 6.90 (d, J = 3.5 Hz, 1H), 4.57 – 4.34 (m, 1H), 2.53 (dd, J = 23.9, 12.3 Hz, 2H), 2.12 – 1.76 (m, 5H), 1.59 – 1.31 (m, 3H). ^{13}C NMR (50 MHz, $CDCl_3$) δ 191.6, 151.4, 144.6, 136.9, 136.2, 135.5, 135.3, 134.0, 132.4, 131.1, 130.7, 129.7, 123.5, 105.5, 101.1, 57.2, 31.1, 25.8, 24.9 FAB-MS m/z: 345.2 $[M+H]^+$ HPLC t_{ret} = 6.152 min

4-(1-cyclohexyl-1,6-dihydroimidazo[4,5-d]pyrrolo[2,3-b]pyridin-2-yl)benzaldehyde ***(15b)*** was obtained from 258 mg **14b** following the above described procedure for **15c** with a reaction time of three hours at ambient temperature. Crude product afforded flash purification

using gradient elution (DCM / MeOH 3 – 10%) to yield 114 mg (64 %) of **15b** as off-white solid. ^{1}H NMR (200 MHz, DMSO) δ 12.03 (br s, 1H), 10.15 (s, 1H), 8.68 (s, 1H), 8.12 (d, *J* = 7.8 Hz, 2H), 7.93 (d, *J* = 7.8 Hz, 2H), 7.61 – 7.50 (m, 1H), 6.84 – 6.76 (m, 1H), 4.55 – 4.24 (m, 1H), 2.46 – 2.23 (m, 2H), 1.88 (dd, *J* = 35.2, 24.7 Hz, 5H), 1.52 – 1.22 (m, 3H). ^{13}C NMR (50 MHz, DMSO) δ 192.5, 150.1, 144.2, 136.2, 136.2, 135.8, 134.7, 132.3, 130.1, 129.4, 123.8, 104.1, 100.0, 56.1, 30.3, 25.1, 24.3 DC-MS (ESI) m/z: 342.9 [M-H]$^-$ HPLC t_{ret} = 6.975 min

5-(1-((1S,2R)-2-methylcyclohexyl)-1,6-dihydroimidazo[4,5-d]pyrrolo[2,3-b]pyridin-2-yl)furan-2-carbaldehyde (15d) To a solution of **14d** (128 mg, 0.26 mmol) in 2.5 ml THF and 2.5 ml MeOH was added 2.5 ml 0.5 M $NaOH_{(aq)}$. The solution was heated to 50 °C water-bath temperature and stirred for 5 hours. TLC indicated complete conversion and the reaction was quenched with sat. NH_4Cl followed by extractive workup with EtOAc (5x10 ml). The combined organic phases were dried over Na_2SO_4 and evaporated. The residue was subjected to flash purification with gradient elution (DCM / MeOH 4 – 10%) yielding 56 mg (63 %) of **15d** as yellow solid. ^{1}H NMR (400 MHz, $CDCl_3$) δ 12.42 (br s, 1H), 9.76 (s, 1H), 8.90 (s, 1H), 7.50 (d, *J* = 3.0 Hz, 1H), 7.39 (d, *J* = 3.6 Hz, 1H), 7.20 (d, *J* = 3.6 Hz, 1H), 6.80 (d, *J* = 3.0 Hz, 1H), 5.07 (dt, *J* = 13.0, 3.6 Hz, 1H), 2.84 – 2.71 (m, 1H), 2.44 – 2.25 (m, 1H), 1.99 – 1.88 (m, 2H), 1.87 – 1.76 (m, 2H), 1.65 – 1.53 (m, 2H), 1.50 – 1.37 (m, 1H), 1.09 (d, *J* = 7.2 Hz, 3H). ^{13}C NMR (100 MHz, $CDCl_3$) δ 177.6, 152.9, 150.5, 145.4, 141.5, 136.8, 135.7, 135.0, 123.6, 121.7, 115.6, 105.3, 99.6, 62.8, 34.4, 32.2, 27.2, 25.6, 19.6, 13.0 DC-MS (ESI) m/z: 371.3 [M+Na]$^+$ HPLC t_{ret} = 6.828 min

1-cyclohexyl-1,6-dihydroimidazo[4,5-d]pyrrolo[2,3-b]pyridine (2) was obtained from 54 mg **13e** following the above described procedure for **15c** with a reaction time of three hours at ambient temperature. Crude product afforded flash purification using gradient elution (DCM / MeOH 3 – 10%) to yield 23 mg (70 %) of **2** as off-white solid. ^{1}H NMR (200 MHz, $CDCl_3$) δ 11.93 (br s, 1H), 8.86 (s, 1H), 8.02 (s, 1H), 7.47 (d, *J* = 3.4 Hz, 1H), 6.69 (d, *J* = 3.4 Hz, 1H), 4.52 (tt, *J* = 11.4, 3.7 Hz, 1H), 2.47 – 2.28 (m, 2H), 2.11 – 1.29 (m, 8H). ^{13}C NMR (50 MHz, $CDCl_3$) δ 144.6, 138.9, 136.1, 135.4, 133.2, 123.7, 105.0, 97.4, 57.0, 33.5, 25.8, 25.5 ESI-HRMS [M+H]$^+$ calculated for $C_{14}H_{16}N_4$: 241.1448, found 241.1450 HPLC t_{ret} = 4.566 min

Preparation of Michael acceptor bearing compounds

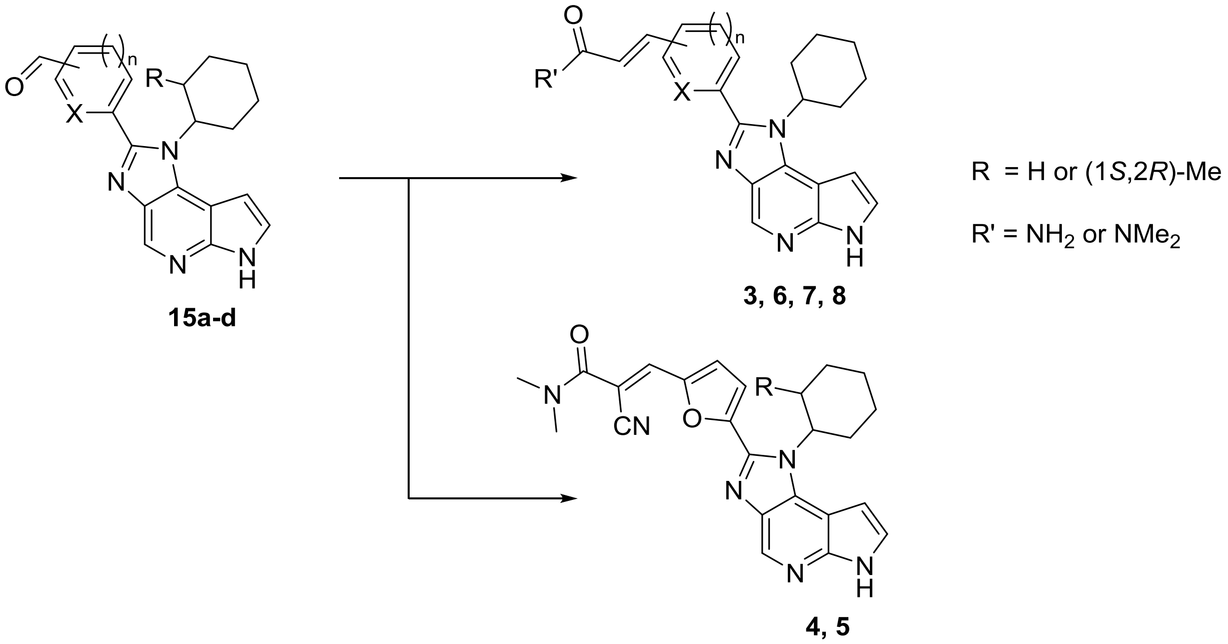

Acrylamides 3 and 6-8 via Horner-Wadsworth-Emmons-reaction

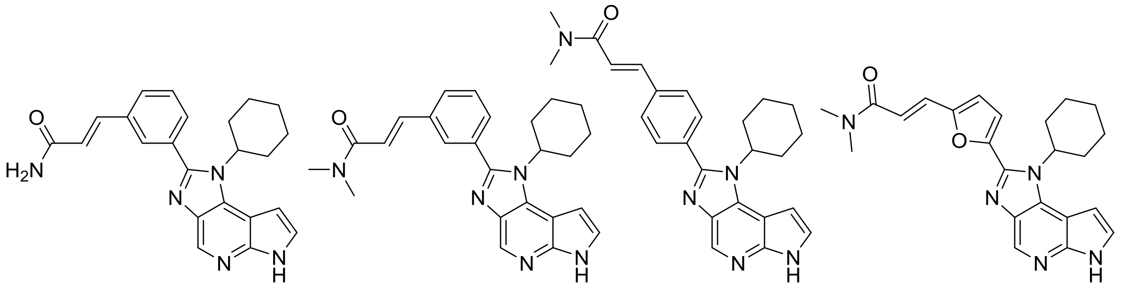

6 7 8 3

(E)-3-(5-(1-cyclohexyl-1,6-dihydroimidazo[4,5-d]pyrrolo[2,3-b]pyridin-2-yl)furan-2-yl)-N,N-dimethylacrylamide (3). In an oven-dried schlenk tube LiCl (8 mg, 180 µmol) was suspended in dry MeCN (2 ml) under argon atmosphere. Subsequently were added diethyl (2-(dimethylamino)-2-oxoethyl)phosphonate (**10b**) (41 mg, 180 µmol) and DBU (27 µl, 180 µmol). After stirring for 10 min at ambient temperature **15c** (40 mg, 120 µmol) was added as suspension in dry chloroform (500 µl). The reaction was complete after another 90 min at ambient temperature. Sat. NH_4Cl was added, followed by extractive workup with DCM (5 x 10 ml). The combined organic extracts were dried over Na_2SO_4, evaporated to dryness and the residue purified by flash chromatography with gradient elution (DCM / MeOH 4-10%) to yield 42 mg (88 %) of **3** as slightly yellowish solid. ^1H NMR (200 MHz, $CDCl_3$) δ 11.92 (br s, 1H), 8.88 (s, 1H), 7.55 (d, J = 15.2 Hz, 1H), 7.49 (d, J = 3.5 Hz, 1H), 7.10 (d, J = 3.6 Hz, 1H), 6.94 (d, J = 15.2 Hz, 1H), 6.86 (d, J = 3.5 Hz, 1H), 6.75 (d, J = 3.6 Hz, 1H), 5.09 – 4.84 (m, 1H), 3.12 (d, J = 17.1 Hz, 6H), 2.67 – 2.35 (m, 2H), 2.18 – 1.78 (m,

5H), 1.64 – 1.37 (m, 3H). ^{13}C NMR (50 MHz, $CDCl_3$) δ 166.8, 153.5, 146.6, 145.4, 143.1, 136.9, 136.0, 134.4, 129.1, 123.7, 116.7, 115.7, 115.4, 105.4, 101.0, 57.2, 37.2, 35.9, 30.6, 25.7, 24.7 ESI-HRMS $[M+H]^+$ calculated for $C_{23}H_{25}N_5O_2$: 404.2081, found: 404.2078 HPLC t_{ret} = 7.148 min (purity 98.4 %)

*(E)-3-(3-(1-cyclohexyl-1,6-dihydroimidazo[4,5-d]pyrrolo[2,3-b]pyridin-2-yl)phenyl)acrylamide (**6**)* was obtained from 55 mg **15a** and 22 mg **10a** following the above described procedure for **3** with a reaction time of two hours at ambient temperature. Crude product afforded flash purification using gradient elution (DCM / MeOH 8 – 16%) to yield 22 mg (78 %) of **6** as off-white solid. ^1H NMR (200 MHz, DMSO) δ 11.97 (bs, 1H), 8.65 (s, 1H), 7.87 (s, 1H), 7.82 – 7.45 (m, 6H), 7.18 (s, 1H), 6.86 – 6.68 (m, 2H), 4.52 – 4.26 (m, 1H), 2.44 – 2.22 (m, 2H), 2.07 – 1.64 (m, 5H), 1.53 – 1.25 (m, 3H). ^{13}C NMR (50 MHz, DMSO) δ 166.5, 151.1, 144.5, 138.4, 135.8, 135.4, 134.8, 132.4, 131.6, 130.2, 129.4, 128.7, 128.5, 124.0, 123.5, 104.3, 100.0, 56.1, 30.3, 25.2, 24.3. ESI-HRMS $[M+H]^+$ calculated for $C_{23}H_{23}N_5O$: 386.1975, found: 386.1973 HPLC t_{ret} = 6.341 min (purity 100 %)

(E)-3-(3-(1-cyclohexyl-1,6-dihydroimidazo[4,5-d]pyrrolo[2,3-b]pyridin-2-yl)phenyl)-N,N-dimethylacrylamide (7) was obtained from 26 mg **15a** and 25 mg **10b** following the above described procedure for **3** with a reaction time of two hours at ambient temperature. Crude product afforded flash purification using gradient elution (DCM / MeOH 3 – 10%) to yield 23 mg (74 %) of **7** as off-white solid. ^1H NMR (200 MHz, $CDCl_3$) δ 11.70 (br s, 1H), 8.89 (s, 1H), 7.89 (s, 1H), 7.81 – 7.43 (m, 5H), 7.00 (d, J = 15.5 Hz, 1H), 6.88 (d, J = 3.3 Hz, 1H), 4.61 – 4.36 (m, 1H), 3.12 (d, J = 19.3 Hz, 6H), 2.62 – 2.35 (m, 2H), 2.08 – 1.71 (m, 5H), 1.62 – 1.32 (m, 3H). ^{13}C NMR (50 MHz, $CDCl_3$) δ 166.5, 152.4, 144.5, 141.4, 136.3, 136.0, 135.4, 133.9, 131.8, 130.3, 129.6, 129.3, 128.6, 123.4, 119.0, 105.5, 101.1, 56.9, 37.6, 36.1, 31.1, 25.8, 24.9 ESI-HRMS $[M+H]^+$ calculated for $C_{25}H_{27}N_5O$: 414.2288, found: 414.2285 HPLC t_{ret} = 6.636 min (purity 98.9 %)

*(E)-3-(4-(1-cyclohexyl-1,6-dihydroimidazo[4,5-d]pyrrolo[2,3-b]pyridin-2-yl)phenyl)-N,N-dimethylacrylamide (**8**)* was obtained from 50 mg **15b** and 49 mg **10b** following the above described procedure for **3** with a reaction time of four hours at ambient temperature. Crude product afforded flash purification using gradient elution (DCM / MeOH 4 – 10%) to yield 42 mg (70 %) of **8** as white solid. ^1H NMR (200 MHz, $CDCl_3$ + MeOD) δ 8.77 (s, 1H), 7.80 – 7.59 (m, 5H), 7.41 (d, J = 3.6 Hz, 1H), 6.99 (d, J = 15.5 Hz, 1H), 6.84 (d, J = 3.6 Hz, 1H), 4.47 (tt, J = 12.3, 4.3 Hz, 1H), 3.13 (d, J = 24.8 Hz, 6H), 2.63 – 2.40 (m, 2H), 2.06 – 1.74 (m, 5H), 1.55 – 1.30 (m, 3H). indole NH was exchanged by MeOD but residual peak was visible

with 0.2 protons. ^{13}C NMR (50 MHz, $CDCl_3$ + MeOD) δ 167.2, 152.7, 144.9, 141.9, 137.2, 136.6, 135.7, 134.1, 132.3, 130.4, 128.5, 123.6, 119.2, 105.5, 101.2, 56.7, 37.4, 35.9, 30.7, 25.4, 24.5 ESI-HRMS $[M+H]^+$ calculated for $C_{25}H_{27}N_5O$: 414.2288, found: 414.2285 HPLC t_{ret} = 6.938 min (purity 100 %)

Cyano acrylamides 4 and 5 via Knoevenagel reaction

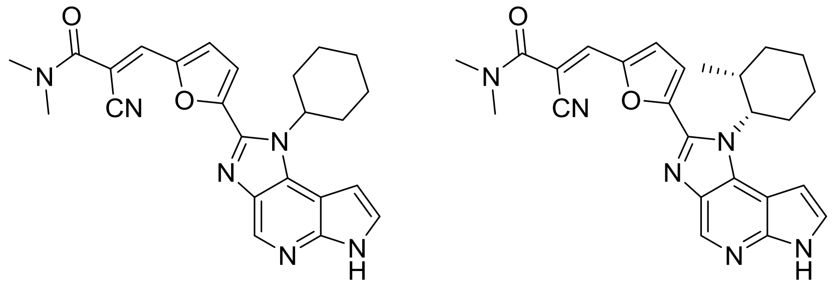

4 **5**

(E/Z)-2-cyano-3-(5-(1-cyclohexyl-1,6-dihydroimidazo[4,5-d]pyrrolo[2,3-b]pyridin-2-yl)furan-2-yl)-N,N-dimethylacrylamide (4). *N,N*-Dimethyl-2-cyanoacetamide (18 mg, 162 µmol) and **15c** (27 mg, 81 µmol) were suspended in EtOH (4 ml) in a screw-cap reaction tube. Piperidine (17 µl, 162 µmol) and acetic acid (10 µl, 162 µmol) were added subsequently. The reaction tube was sealed and the reaction was placed in a heating block at 80 °C. After one hour the reaction was cooled to ambient temperature, diluted with EtOAc and poured on brine. The phases were separated and the aqueous phase was extracted with EtOAc (4 x 10 ml). The combined organic phases were dried over Na_2SO_4, evaporated to dryness and the residue purified by flash chromatography (gradient elution DCM / MeOH 4-16%) to yield 32 mg (93 %) of **4** as yellow solid. ^1H NMR (400 MHz, DMSO) δ 12.05 (br s, 1H), 8.72 – 8.63 (m, 1H), 7.78 (s, 1H), 7.56 (s, 1H), 7.49 (d, J = 3.5 Hz, 1H), 7.39 (d, J = 3.5 Hz, 1H), 6.86 – 6.78 (m, 1H), 4.99 – 4.86 (m, 1H), 3.21 – 2.87 (m, 6H), 2.42 – 2.27 (m, 2H), 2.02 (d, J = 10.7 Hz, 2H), 1.95 – 1.84 (m, 2H), 1.80 – 1.70 (m, 1H), 1.66 – 1.42 (m, 3H). ^{13}C NMR (100 MHz, DMSO) δ 162.8, 149.5, 148.4, 144.6, 140.5, 136.3, 135.4, 135.1, 132.8, 124.1, 121.9, 116.3, 115.9, 104.1, 101.9, 100.3, 56.1, 30.4, 30.0, 24.9, 24.3 ESI-HRMS $[M+H]^+$ calculated for $C_{24}H_{24}N_6O_2$: 429.2034, found: 429.2045 HPLC t_{ret} = 6.172 min and 6.985 min (E/Z mixture, purity 100 %)

(E/Z)-2-cyano-N,N-dimethyl-3-(5-(1-((1S,2R)-2-methylcyclohexyl)-1,6-dihydroimidazo[4,5-d]pyrrolo[2,3-b]pyridin-2-yl)furan-2-yl)acrylamide (5). *N,N*-Dimethyl-2-cyanoacetamide (12 mg, 108 µmol) and **15d** (27 mg, 72 µmol) were suspended in *i*PrOH (2 ml) in a screw-cap reaction tube. Piperidine was added as 1 M solution in *i*PrOH (14 µl, 14 µmol). The reaction

tube was sealed and the reaction was placed in a heating block at 60 °C. After six hours the reaction was cooled to ambient temperature, diluted with EtOAc and poured on sat. NH_4Cl. The phases were separated and the aqueous phase was extracted with EtOAc (5x10 ml). The combined organic phases were dried over Na_2SO_4, evaporated to dryness and the residue purified by flash chromatography with gradient elution (DCM / MeOH 4 - 12%) to yield 17 mg (53 %) of **5** as yellow solid. ^{1}H NMR (400 MHz, DMSO) δ 12.04 (br s, 1H), 8.66 (d, *J* = 5.0 Hz, 1H), 7.76 (s) and 7.63 (s, 1H), 7.56 – 7.50 (m, 1H), 7.48 – 7.42 and 7.29 – 7.22 (m, 1H), 7.14 (s, 1H), 6.80 (s, 1H), 5.09 – 4.87 (m, 1H), 3.18 – 2.82 (m, 6H), 2.49 – 2.37 (m, 1H), 2.17 – 1.77 (m, 4H), 1.68 – 1.39 (m, 4H), 0.87 – 0.76 (m, 3H). ^{13}C NMR (100 MHz, DMSO) δ 162.7, 161.4, 148.9, 148.7, 148.4, 148.3, 144.9, 140.7, 140.5, 136.2, 136.2, 135.6, 134.2, 134.1, 134.1, 134.0, 131.6, 124.0, 120.8, 119.6, 117.0, 116.9, 116.3, 115.8, 104.3, 104.2, 102.3, 98.9, 61.2, 61.1, 37.3, 34.3, 33.7, 33.6, 31.2, 31.1, 26.1, 26.0, 25.0, 24.9, 18.9 ESI-HRMS $[M+H]^+$ calculated for $C_{25}H_{26}N_6O_2$: 443.2190, found: 443.2207 HPLC t_{ret} = 5.885 min and 6.538 min (E/Z mixture, purity 97.1 %)

Supplemental References

Bauer, S.M., Gehringer, M., and Laufer, S.A. (2014). A direct enzyme-linked immunosorbent assay (ELISA) for the quantitative evaluation of Janus Kinase 3 (JAK3) inhibitors. Analytical Methods *6*, 8817-8822.

Beaulieu, P.L., Haché, B., and von Moos, E. (2003). A Practical Oxone®-Mediated, High-Throughput, Solution-Phase Synthesis of Benzimidazoles from 1,2-Phenylenediamines and Aldehydes and its Application to Preparative Scale Synthesis. Synthesis *2003*, 1683-1692.

Chen, V.B., Arendall, W.B., III, Headd, J.J., Keedy, D.A., Immormino, R.M., Kapral, G.J., Murray, L.W., Richardson, J.S., and Richardson, D.C. (2010). MolProbity: all-atom structure validation for macromolecular crystallography. Acta Crystallographica Section D *66*, 12-21.

Davis, M.I., Hunt, J.P., Herrgard, S., Ciceri, P., Wodicka, L.M., Pallares, G., Hocker, M., Treiber, D.K., and Zarrinkar, P.P. (2011). Comprehensive analysis of kinase inhibitor selectivity. Nat Biotech *29*, 1046-1051.

Emsley, P., Lohkamp, B., Scott, W.G., and Cowtan, K. (2010). Features and development of Coot. Acta Crystallographica Section D *66*, 486-501.

Evans, P. (2006). Scaling and assessment of data quality. Acta Crystallographica Section D *62*, 72-82.

Frigerio, M., Santagostino, M., and Sputore, S. (1999). A User-Friendly Entry to 2-Iodoxybenzoic Acid (IBX). The Journal of Organic Chemistry *64*, 4537-4538.

Gehringer, M., Forster, M., Pfaffenrot, E., Bauer, S.M., and Laufer, S.A. (2014). Novel Hinge-Binding Motifs for Janus Kinase 3 Inhibitors: A Comprehensive Structure–Activity Relationship Study on Tofacitinib Bioisosteres. ChemMedChem *9*, 2516-2527.

Goedken, E.R., Argiriadi, M.A., Banach, D.L., Fiamengo, B.A., Foley, S.E., Frank, K.E., George, J.S., Harris, C.M., Hobson, A.D., Ihle, D.C., *et al.* (2015). Tricyclic Covalent Inhibitors Selectively Target Jak3 through an Active Site Thiol. Journal of Biological Chemistry *290*, 4573-4589.

Ireland, R.E., and Liu, L. (1993). An improved procedure for the preparation of the Dess-Martin periodinane. The Journal of Organic Chemistry *58*, 2899-2899.

Kem, K.M., Nguyen, N.V., and Cross, D.J. (1981). Phase-transfer-catalyzed Michaelis-Becker reaction. The Journal of Organic Chemistry *46*, 5188-5192.

Knupp, G., and Frahm, A.W. (1984). Asymmetrische reduktive Aminierung von Cycloalkanonen 2. Synthese und absolute Konfiguration 2-substituierter Cyclohexanamine. Chemische Berichte *117*, 2076-2098.
London, N., Miller, R.M., Krishnan, S., Uchida, K., Irwin, J.J., Eidam, O., Gibold, L., Cimermančič, P., Bonnet, R., Shoichet, B.K.*, et al.* (2014). Covalent docking of large libraries for the discovery of chemical probes. Nat Chem Biol *10*, 1066-1072.
Machleidt, T., Woodroofe, C.C., Schwinn, M.K., Méndez, J., Robers, M.B., Zimmerman, K., Otto, P., Daniels, D.L., Kirkland, T.A., and Wood, K.V. (2015). NanoBRET—A Novel BRET Platform for the Analysis of Protein–Protein Interactions. ACS Chemical Biology *10*, 1797-1804.
McCoy, A.J., Grosse-Kunstleve, R.W., Adams, P.D., Winn, M.D., Storoni, L.C., and Read, R.J. (2007). Phaser crystallographic software. Journal of Applied Crystallography *40*, 658-674.
Murshudov, G.N., Skubak, P., Lebedev, A.A., Pannu, N.S., Steiner, R.A., Nicholls, R.A., Winn, M.D., Long, F., and Vagin, A.A. (2011). REFMAC5 for the refinement of macromolecular crystal structures. Acta Crystallographica Section D *67*, 355-367.
Neumann, L., von Konig, K., and Ullmann, D. (2011). HTS reporter displacement assay for fragment screening and fragment evolution toward leads with optimized binding kinetics, binding selectivity, and thermodynamic signature. Methods Enzymol *493*, 299-320.
Powell, H.R., Johnson, O., and Leslie, A.G.W. (2013). Autoindexing diffraction images with iMosflm. Acta Crystallographica Section D *69*, 1195-1203.
Robers, M.B., Dart, M.L., Woodroofe, C.C., Zimprich, C.A., Kirkland, T.A., Machleidt, T., Kupcho, K.R., Levin, S., Hartnett, J.R., Zimmerman, K.*, et al.* (2015). Target engagement and drug residence time can be observed in living cells with BRET. Nat Commun *6*, 10091.
Smith, G.A., Uchida, K., Weiss, A., and Taunton, J. (2016). Essential biphasic role for JAK3 catalytic activity in IL-2 receptor signaling. Nat Chem Biol *12*, 373-379.
Soth, M., Hermann, J.C., Yee, C., Alam, M., Barnett, J.W., Berry, P., Browner, M.F., Frank, K., Frauchiger, S., Harris, S.*, et al.* (2013). 3-Amido pyrrolopyrazine JAK kinase inhibitors: development of a JAK3 vs JAK1 selective inhibitor and evaluation in cellular and in vivo models. J Med Chem *56*, 345-356.
Tan, L., Akahane, K., McNally, R., Reyskens, K.M.S.E., Ficarro, S.B., Liu, S., Herter-Sprie, G.S., Koyama, S., Pattison, M.J., Labella, K.*, et al.* (2015). Development of Selective Covalent Janus Kinase 3 Inhibitors. Journal of Medicinal Chemistry *58*, 6589-6606.
Thoma, G., Drückes, P., and Zerwes, H.-G. (2014). Selective inhibitors of the Janus kinase Jak3—Are they effective? Bioorganic & medicinal chemistry letters *24*, 4617-4621.
Thoma, G., Nuninger, F., Falchetto, R., Hermes, E., Tavares, G.A., Vangrevelinghe, E., and Zerwes, H.-G. (2011). Identification of a Potent Janus Kinase 3 Inhibitor with High Selectivity within the Janus Kinase Family. Journal of Medicinal Chemistry *54*, 284-288.
Zhang, J., Polishchuk, E.A., Chen, J., and Ciufolini, M.A. (2009). Development of an Oxazole Conjunctive Reagent and Application to the Total Synthesis of Siphonazoles. The Journal of Organic Chemistry *74*, 9140-9151.
