# Supplementary material for: Selective JAK3 Inhibitors with a Covalent Reversible Binding Mode Targeting a New Induced Fit Binding Pocket
Source: Cell Chem Biol. 2016 Nov 17;23(11):1335–40. doi: 10.1016/j.chembiol.2016.10.008 (PMC5119931; doi:10.1016/j.chembiol.2016.10.008)
Supplement: Document S2. Article plus Supplemental Information [file mmc3.pdf]

Brief Communication

Cell Chemical Biology

Selective JAK3 Inhibitors with a Covalent Reversible Binding Mode Targeting a New Induced Fit Binding Pocket

Graphical Abstract

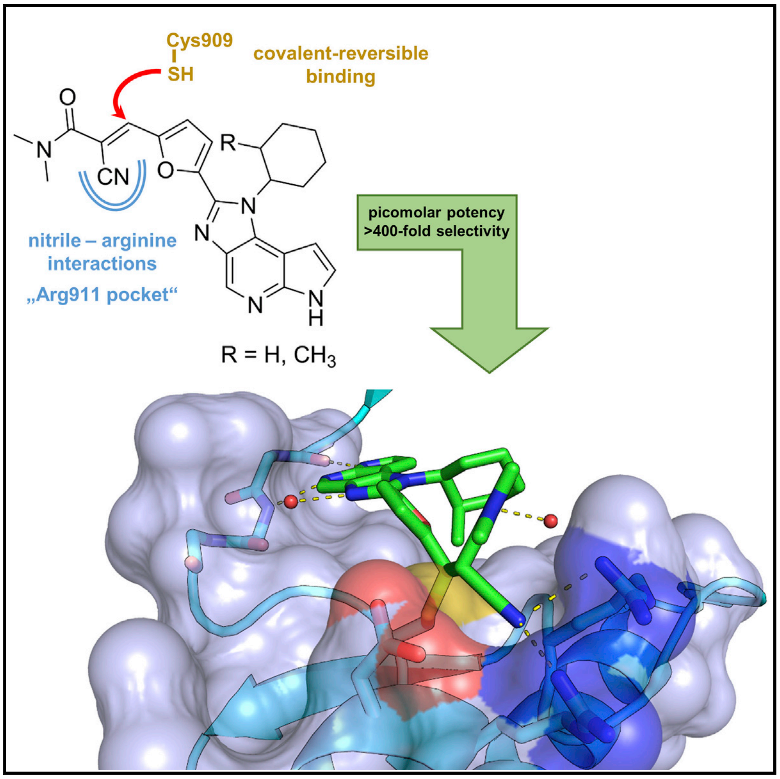

Highlights

- Identification and characterization of novel covalent reversible JAK3 inhibitors
- Picomolar affinities along with both high isoform and kinome selectivity is achieved
- Covalent-reversible interaction and a new induced binding pocket confirmed by X-ray structures
- High potency and selectivity are successfully proven in cellular models

Authors

Michael Forster, Apirat Chaikuad, Silke M. Bauer, ..., Kamran Ghoreschi, Stefan Knapp, Stefan A. Laufer

Correspondence

knapp@pharmchem.uni-frankfurt.de (S.K.), stefan.laufer@uni-tuebingen.de (S.A.L.)

In Brief

JAK1/3 signaling plays a major role in immunological and inflammatory processes, but the reciprocal dependency of this receptor-sharing kinase pair still remains unclear. Forster et al. report highly selective covalent reversible JAK3 inhibitors as promising tools to elucidate this issue.

Accession Numbers

5LWM
5LWN

Forster et al., 2016, Cell Chemical Biology *23*, 1335–1340
November 17, 2016 © 2016 The Authors. Published by Elsevier Ltd.
http://dx.doi.org/10.1016/j.chembiol.2016.10.008

Please cite this article as: Forster et al., Selective JAK3 Inhibitors with a Covalent Reversible Binding Mode Targeting a New Induced Fit Binding Pocket, Cell Chemical Biology (2016), http://dx.doi.org/10.1016/j.chembiol.2016.10.008

Cell Chemical Biology
Brief Communication

CellPress

Selective JAK3 Inhibitors with a Covalent Reversible Binding Mode Targeting a New Induced Fit Binding Pocket

Michael Forster,[1] Apirat Chaikuad,[2] Silke M. Bauer,[1] Julia Holstein,[3] Matthew B. Robers,[4] Cesear R. Corona,[4] Matthias Gehringer,[1,5] Ellen Pfaffenrot,[1] Kamran Ghoreschi,[3] Stefan Knapp,[2,6,*] and Stefan A. Laufer[1,7,*]

[1]Department of Pharmaceutical/Medicinal Chemistry, Eberhard-Karls-University Tuebingen, Auf der Morgenstelle 8, 72076 Tuebingen, Germany

[2]Nuffield Department of Clinical Medicine, Structural Genomics Consortium and Target Discovery Institute, University of Oxford, Old Road Campus Research Building, Roosevelt Drive, Oxford OX3 7DQ, UK

[3]Department of Dermatology, University Medical Center, Eberhard-Karls-University Tuebingen, Liebermeisterstraße 25, 72076 Tuebingen, Germany

[4]Promega Corporation, 2800 Woods Hollow Road, Madison, WI 53711, USA

[5]Present address: Department of Chemistry and Applied Biosciences, Institute of Pharmaceutical Sciences, Swiss Federal Institute of Technology (ETH) Zurich, Vladimir-Prelog-Weg 1–5/10, 8093 Zurich, Switzerland

[6]Present address: Institute for Pharmaceutical Chemistry, Johann Wolfgang Goethe-University and Buchmann Institute for Molecular Life Sciences, Max-von-Laue-Straße 9, 60438 Frankfurt am Main, Germany

[7]Lead Contact

*Correspondence: knapp@pharmchem.uni-frankfurt.de (S.K.), stefan.laufer@uni-tuebingen.de (S.A.L.)

http://dx.doi.org/10.1016/j.chembiol.2016.10.008

SUMMARY

Janus kinases (JAKs) are a family of cytoplasmatic tyrosine kinases that are attractive targets for the development of anti-inflammatory drugs given their roles in cytokine signaling. One question regarding JAKs and their inhibitors that remains under intensive debate is whether JAK inhibitors should be isoform selective. Since JAK3 functions are restricted to immune cells, an isoform-selective inhibitor for JAK3 could be especially valuable to achieve clinically more useful and precise effects. However, the high degree of structural conservation makes isoform-selective targeting a challenging task. Here, we present picomolar inhibitors with unprecedented kinome-wide selectivity for JAK3. Selectivity was achieved by concurrent covalent reversible targeting of a JAK3-specific cysteine residue and a ligand-induced binding pocket. We confirmed that in vitro activity and selectivity translate well into the cellular environment and suggest that our inhibitors are powerful tools to elucidate JAK3-specific functions.

INTRODUCTION

While the other isoforms of the Janus kinase family (JAK1, JAK2, and TYK2) have a broad spectrum of functions in different tissues, JAK3 plays a specific role in the development of immune-competent cells (Ghoreschi et al., 2009). The key function of JAK3 in the immune system is further supported by the fact that loss-of-function mutations of JAK3 cause severe combined immunodeficiency syndrome (SCID). Therefore, JAK3-selective inhibitors are considered as promising candidates for immunosuppressive and anti-inflammatory therapies (Pesu et al., 2005). However, the sufficiency of specific JAK3 inhibition for efficient immunosuppression is heavily debated. The cause of the controversy rests on the invariable co-localization of JAK3 and JAK1 on common γ chain (γ_c) cytokine receptor dimers, suggesting that dual JAK1 and JAK3 inhibition is required for efficient suppression of cytokine signaling (Haan et al., 2011; Thorarensen et al., 2014). To resolve this enigma, highly JAK3-selective chemical probes are required. To date, only a few compounds with appropriate isoform selectivity for JAK3 have been reported. The current gold standard for investigating JAK-dependent signaling is the selective pan-JAK inhibitor tofacitinib (**1**, Figure 1) (Knapp et al., 2013). Although initially claimed as JAK3 specific (Flanagan et al., 2010), further studies demonstrated the poor selectivity of **1** within the JAK family (Thoma et al., 2014). Due to the immense structural conservation among the JAKs, isoform selectivity remains a challenging task. Thoma et al. (2011) developed reversible maleinimide-derived inhibitors with reasonable JAK3 selectivity (>120 fold), which have been utilized with limited success to probe the JAK1/3 dependency issue (Haan et al., 2011). Very recently, Goedken et al. (2015) and Tan et al. (2015) reported irreversible JAK3 inhibitors showing good isoform selectivity but also potent off-target activity in the remaining kinome. Merck patented several irreversible acrylamide-based inhibitors (Ahearn et al., 2013), and recently Smith et al. (2016) utilized an inhibitor from this structural class for comprehensive investigation of the time dependency of JAK1/3 signaling in T cells. Although high JAK3 isoform selectivity was demonstrated, kinome-wide selectivity was not particularly investigated. Furthermore, the collective consequences of JAK3 inhibition in an exclusively irreversible covalent manner are still unclarified and may not be favorable for all purposes. An alternative to classical Michael acceptors are covalent reversible cyano-acrylamide-based inhibitors. This principle

This is an open access article under the CC BY license (http://creativecommons.org/licenses/by/4.0/).

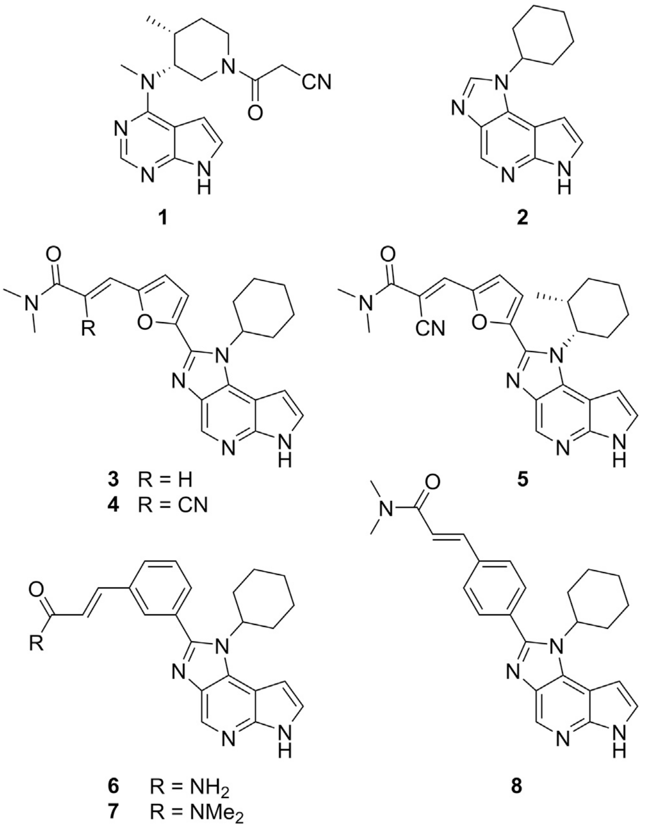

Figure 1. Structures of Tofacitinib 1, Lead Compound 2, and Novel JAK3 Inhibitors 3–8

See also Tables S1, S2, S3, and S6.

was successfully applied by London et al. (2014) to obtain JAK3 inhibitors with reasonable in vitro activity and isoform selectivity, but without a proven cellular activity and kinome-wide selectivity. The present study reports a novel class of covalent reversible JAK3 inhibitors providing both high isoform and kinome selectivity as well as potent cellular activity and selectivity.

RESULTS AND DISCUSSION

Identification of Covalent Reversible JAK3 Inhibitors 4 and 5

In our quest for a highly selective JAK3 probe, we aimed to exploit a non-catalytic cysteine (C909), which is not present in the other JAK family members. C909 is situated in the solvent-exposed front part of the ATP binding site, where the other isoforms possess a serine residue (Chrencik et al., 2010). The nucleophilic nature of the cysteine thiol group can be utilized to covalently trap inhibitors bearing an electrophilic group (Singh et al., 2010). Besides JAK3, only ten human kinases feature a cysteine at an equivalent position (Liu et al., 2013).

Based on modeling studies, we identified compound **2** (Figure 1) with a half maximal inhibitory concentration (IC_{50}) of 63 nM as a reasonable starting point for the development of covalent JAK3 inhibitors targeting C909. We substituted the imidazole C2 atom with suitable linker moieties bearing an electrophilic warhead. Initial efforts focused on *para*- and *meta*-substituted phenyl linkers and simple acrylamides as Michael acceptors. This strategy provided limited success since all compounds (**6–8**, Figure 1) in this series exhibited decreased inhibitory activity compared with **2** (Table S1). Switching the linker from phenyl to a 2,5-disubstituted furyl moiety furnished more promising results. Compound **3** (Figure 1) bearing a classical acrylamide Michael acceptor demonstrated a slightly higher inhibitory activity (IC_{50} = 51 nM) than template **2**. However, the increase in activity was not substantial enough to assume covalent binding. Accordingly, the initial design strategy was revised in two ways. First, the reactivity of the Michael acceptor was tuned by introducing an additional nitrile group at the αC atom. Cyano-acrylamides were postulated as covalent reversible Michael acceptors, since the covalent bond formation can be reversed under physiological conditions (Serafimova et al., 2012). As a second feature, an additional methyl group was introduced at position 2 of the cyclohexyl moiety to mimic the chiral side chain of **1** more appropriately. Both modifications were well tolerated and yielded highly potent compounds **4** and **5** (Figure 1) with IC_{50} values of 9 nM and 17 nM, respectively, which are close to the lower detection limit of our ELISA.

Compounds 4 and 5 Demonstrate High JAK Isoform and Kinome Selectivity

This 4- to 7-fold increase in JAK3 inhibitory activity compared with **2** prompted us to determine the JAK isoform selectivity for compounds **1**, **3**, **4**, and **5** in a commercial assay (Kinase HotSpot, Reaction Biology Corp.). As previously observed (Gehringer et al., 2014), this assay format is more sensitive and therefore provides comparatively lower IC_{50} values than the previously applied ELISA (Table S1). The selectivity profiles of **1** and the classical amide-derived Michael acceptor **3** are significantly different from cyano-acrylamides **4** and **5**. While **3** and **1** were reasonably potent (IC_{50} = 22 nM and 292 pM, respectively) but unselective within the JAK family, both **4** and **5** exhibited JAK3 IC_{50} values in the picomolar range (127 pM and 154 pM, respectively), even lower than the reference compound **1**, and demonstrated 400-, 2,700- and 3,600-fold or 400-, 1,700-, and 5,800-fold selectivity over JAK1, JAK2, and TYK2, respectively (Table S1). We screened **4** and **5** against a panel of 410 kinases (Kinase 410-Profiler, ProQinase) at concentrations of 100 nM and 500 nM. Both compounds had no relevant effect on the activity of any tested kinases except JAK3 at a concentration of 100 nM. At 500 nM, compound **4** moderately inhibited 11 other kinases besides JAK3 with residual activities below 50%, while compound **5** revealed only one off-target (Table S2). The somewhat better selectivity profile of **5** can be attributed to the additional exocyclic methyl group, which is supposed to be also the key driver for the exceptional kinome selectivity of **1** (Chrencik et al., 2010). It is noteworthy that both compounds show no significant activity against the other ten kinases carrying a cysteine at the equivalent position. To verify these results in a cellular setting, we used a bioluminescence resonance energy transfer (BRET) assays in HeLa cells expressing the NanoLuc fused tyrosine kinases BTK, BLK, and TEC, which all harbor a cysteine at the same position. We detected no measurable interaction of **4** with TEC or BTK, and only BLK interacted weakly at micromolar concentrations (Figures S1D–S1F). Thus, **4** and **5**

Please cite this article as: Forster et al., Selective JAK3 Inhibitors with a Covalent Reversible Binding Mode Targeting a New Induced Fit Binding Pocket, Cell Chemical Biology (2016), http://dx.doi.org/10.1016/j.chembiol.2016.10.008

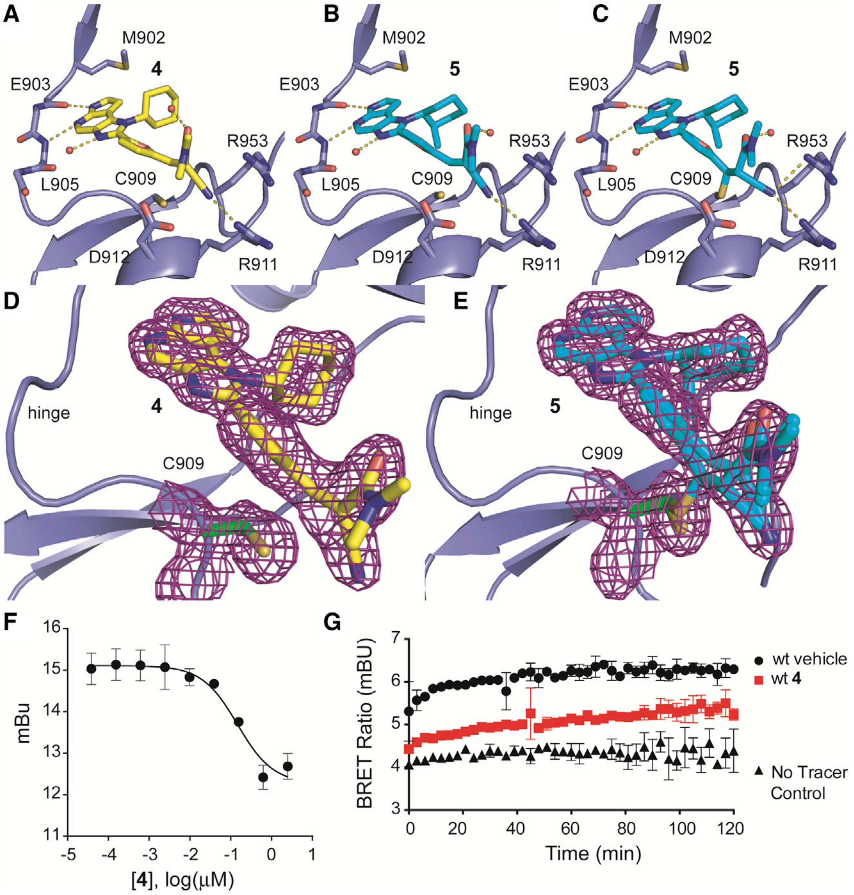

Figure 2. Co-crystal Structures of JAK3 and Compounds 4 and 5

(A) **4** non-covalently bound to JAK3 (PDB: 5LWM).
(B) **5** non-covalently bound to JAK3 (PDB: 5LWN).
(C) **5** covalently bound to JAK3 (PDB: 5LWN).
(D) $2F_o - F_c$ omitted electron density map of **4**-JAK3.
(E) $2F_o - F_c$ omitted electron density map of **5**-JAK3.
(F) Dose-dependent BRET experiment showing displacement of the fluorescent tracer in NanoLuc tagged JAK3 in HeLa cells.
(G) Residence time experiment using BRET. HeLa cells expressing NanoLuc JAK3 were equilibrated with 1 μM of **4**, washed out, and treated with high concentrations of tracer. Displacement of **4** was monitored by BRET. BRET levels of full occupancy control were reached after approx. 1 hr.
Data shown are means ± SD of quadruplicates. See also Figures S1, S2, and Table S4.

represent two excellent tool compounds that compare favorably with other JAK3 inhibitors published in the current peer-reviewed literature (Table S6). We also determined binding kinetics of **4** on all JAK isoforms (Proteros Reporter Displacement Assay, Proteros Biostructures). While **4** rapidly diffuses from JAK1, JAK2, and TYK2 with residence times below 1.4 min (lower detection limit), a prolonged residence time of 50 min on JAK3 was observed (Table S3). We used BRET to reveal the binding characteristics of **4** in living cells. In dose-response experiments, we observed efficient displacement of the fluorescent tracer at around 100 nM (Figure 2F), demonstrating on-target activity and good cellular activity of **4**. In washout experiments we also determined the dissociation behavior of **4** and observed recovery of the BRET ratio after about 1 hr in agreement with the binding kinetic experiments described above (Figure 2G). This combination of inhibitory and kinetic JAK3 selectivity reinforced our assumption of covalent reversible binding of **4** and **5**.

JAK3 Co-crystal Structures of 4 and 5 Confirm Covalent Reversible Binding and Reveal a New Binding Pocket

High resolution crystal structures of **4** and **5** in complex with JAK3 were determined (Figure 2). Both compounds showed the expected orientation of the hinge binding motif featuring the typical bidentate hydrogen bonding pattern. While the complex of JAK3 with **4** only revealed the non-covalent binding mode, the structure in complex with **5** displayed the coexistence of the non-covalently and the covalently bound inhibitor **5** (Figures 2A–2C). The presence of both binding modes underlines the highly reversible character of the covalent interaction since the crystal structure represents an equilibrium state between covalently and non-covalently bound **5**. The coexistence of both binding modes in the crystal structure with **5** was demonstrated by difference electron density maps after refinement (Figure S2), considering only the reversible, the irreversible, or both binding modes, and confirmed by electrospray ionization mass spectrometry (ESI-MS) (Figure S2). Although three JAK3 crystal structures with irreversible inhibitors have recently been published (PDB: 4QPS, 4Z16, 4V0G), the structural model presented here is the first one depicting the interaction of a covalent reversible inhibitor with JAK3 as highlighted by electron density maps that clearly distinguish between covalent and non-covalent binding modes (Figures 2D and 2E). To the best of our knowledge, the simultaneous presence of both binding modes has not been observed for any cyanoacrylamide-derived inhibitor before. Therefore, our structure further validates the concept of covalent reversible enzyme inhibition with Michael acceptors. Furthermore, we observed a yet unprecedented binding pocket formed by R911, D912, and R953. This unique feature is induced by interactions of the nitrile moieties of **4** and **5** with R911, thereby generating a distinct cavity in the protein surface (Figure 3B). Structural comparison with other JAK3 structures reveals that the side chain of R911 is dramatically reoriented and forms a hydrogen bond with the inhibitors nitrile and nitrogen. The guanidine moiety of R953 is flipped approximately 180° toward the nitrile group of **4** and thereby forms the ceiling of this arginine pocket (Figures 3A and 3B). Alignment of the other 16 available JAK3 crystal structures revealed that the orientation of the aforementioned arginine residues is conserved in those structures, confirming the induction of the arginine pocket to be a unique feature of our inhibitor class (Figure 3C). To assess the contribution of this cavity to the outstanding selectivity of **4** and **5**, we aligned the amino acid

Please cite this article as: Forster et al., Selective JAK3 Inhibitors with a Covalent Reversible Binding Mode Targeting a New Induced Fit Binding Pocket, Cell Chemical Biology (2016), http://dx.doi.org/10.1016/j.chembiol.2016.10.008

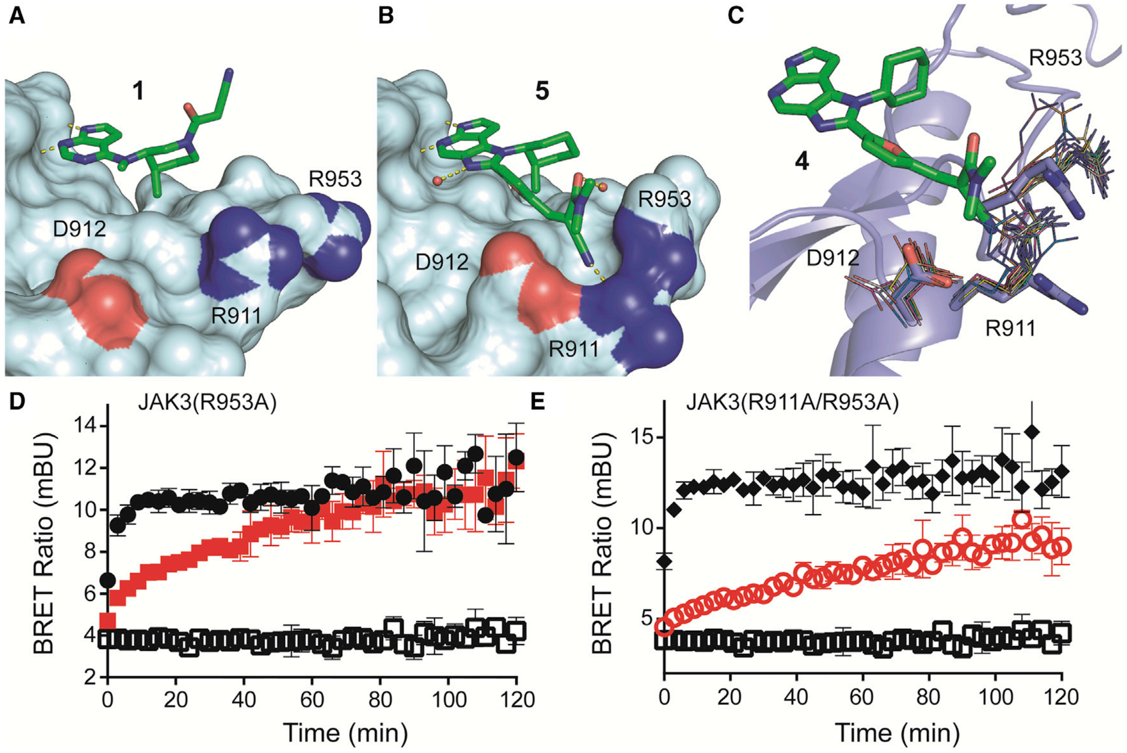

Figure 3. Induced Binding Pocket Around R911

(A) Protein surface of **1** bound to JAK3 (PDB: 3LXK).

(B) Protein surface of **5** bound to JAK3 (PDB: 5LWN). N-terminal lobes are omitted for clarity and heteroatoms of the residues of R911, D912, and R953 are colored. Comparison of (A) and (B) shows the rearrangement of these amino acid side chains upon formation of the arginine pocket.

(C) Alignment of 16 JAK3 crystal structures with **4**-JAK3. Residues of R911, D912, and R953 are shown as sticks (**4**-JAK3, PDB: 5LWM) or as lines (other structures, PDB: 3LXK, 4QT1, 4QPS, 4RIO, 4ZEP, 4I6Q, 3ZC6, 4HVD, 4HVG, 4HVH, 4HVI, 3PJC, 3LXL, 1YVJ, 4V0G, 4Z16). The deviating conformation of these side chains is unique to our structure, while it is relatively conserved among the other JAK3 structures.

(D and E) BRET experiments measuring residence time of **4** in HeLa cells expressing the NanoLuc mutant JAK3 R953A (D) or the NanoLuc double mutant JAK3 R911A/R953A (E). BRET traces of vehicle-treated cells are shown as filled black spheres (D) and diamonds (E), traces of inhibitor-treated cells are shown in red, and no tracer control is shown as empty squares. Washout experiments show that both mutants retain slow binding kinetics of **4**.

Data shown are mean ± SD of quadruplicates. See also Figure S1, Tables S4, and S5.

sequence of the arginine pocket in JAK3 with the corresponding regions of kinases carrying a reactive cysteine at an equivalent position (Table S5). While the R953 (JAK3 numbering) is mainly conserved, JAK3-D912 is replaced by an asparagine, glutamate, or lysine in five of the ten other kinases. Interestingly, an arginine residue at position 911 is unique to JAK3. In most of the other kinases, this position is occupied by a bulkier and less flexible leucine residue incapable of forming polar interactions. In contrast, the amino acids forming the arginine pocket are mainly conserved in the JAK family (Table S5) except for JAK1, possessing a lysine residue instead of R911 and a glutamate instead of D912. Based on these observations, we assume that the interaction of JAK3 R911 with the nitrile substituent of compounds **4** and **5** constitutes a second key feature for inhibitor selectivity and potency. BRET experiments using the JAK3 mutant R953A, as well as the double mutant R911A/R953A, showed similar affinity of **4** (Figures S1A–S1C), suggesting that the induction of the arginine cavity did not significantly affect inhibitor potency. Moreover, the inhibitor dissociating rates of **4** in the two JAK mutants R953A and R911A/R953A were comparable with the ones observed in the wild-type protein (Figures 3D and 3E). However, the induced pocket as well as the hydrogen bonds formed with **4** and **5** in combination with the covalent cysteine targeting represent a unique dual selectivity filter of these inhibitors outside the JAK family (Muller et al., 2015).

Compounds 4 and 5 Selectively Inhibit JAK3 Signaling in Human CD4$^+$ T Cells

To assess the functional selectivity of **4** and **5**, we used functional human CD4$^+$ T cells that were stimulated with cytokines activating the JAK/STAT pathway. First, we stimulated the T cells possessing the γ_c receptor using interleukin (IL)-2. Consecutive activation of JAK3 and JAK1 and subsequent STAT5 phosphorylation allowed comparison of **4** and **5** with the clinically established pan-JAK inhibitor **1**. Compounds **4** and **5** abrogated γ_c cytokine signaling even at 50 nM or 100 nM, respectively, while a higher concentration (300 nM) of **1** was necessary to completely block STAT5 phosphorylation (Figure 4A). A similar result was observed when stimulating T cells with IL-4, where all three compounds completely block the JAK1/JAK3-mediated STAT6 phosphorylation at 300 nM (Figure 4B). As reported earlier (Ghoreschi et al., 2011), STAT3 phosphorylation via

CellPress

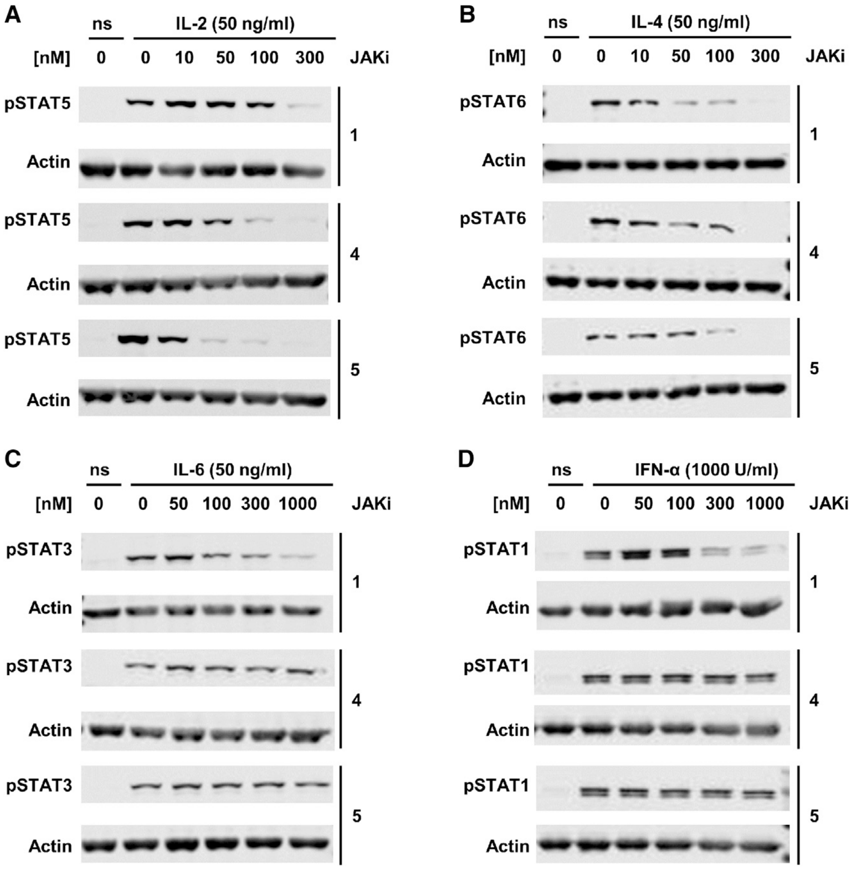

Figure 4. Selective Inhibition of JAK3-Mediated Cytokine Signaling by Compounds 4 and 5

Human CD4+ T cells were pre-incubated for 1 hr with the indicated concentrations of the JAK inhibitors (JAKi) **1**, **4** or **5** and stimulated for 30 min with IL-2 (activates JAK3/JAK1) (A), IL-4 (activates JAK3/JAK1) (B), IL-6 (activates JAK2/JAK1/TYK2) (C) or IFN-α (activates JAK1/TYK2) (D). Phosphorylation of STAT5 (A), STAT6 (B), STAT3 (C), or STAT1 (D) was determined by phospho-specific Abs and immunoblotting (ns, no cytokine stimulation). Levels of actin were determined to show equal loading. See also Table S1.

JAK1, JAK2, and TYK2, triggered by stimulation with IL-6, is inhibited by **1** in a dose-dependent manner at concentrations $\geq$300 nM. In sharp contrast, **4** and **5** did not affect STAT3 activation at doses up to 1,000 nM (Figure 4C), confirming the selectivity toward JAK3 in functional cells. The selectivity difference between our compounds and **1** was even more pronounced when T cells were stimulated with interferon (IFN)-α. While **1** clearly inhibits IFN-α-mediated JAK1/TYK2 signaling at concentrations >100 nM, compounds **4** and **5** did not influence pSTAT1 levels at concentrations up to 1,000 nM (Figure 4D). These results demonstrate that the JAK3 selectivity of **4** and **5** observed in enzyme assays are maintained in a cellular context.

SIGNIFICANCE

In this study, a new class of covalent reversible JAK3 inhibitors was developed. Crystallographic data confirmed a reversible covalent binding mode as well as a previously unseen binding cavity induced by a nitrile-arginine interaction. The combination of both structural features paved the way for a new generation of JAK3-selective inhibitors. With compounds 4 and 5, we provide JAK3 inhibitors with picomolar affinities and outstanding selectivity within the JAK family and against the whole kinome. Moreover, it was demonstrated that activity and selectivity translate well in a cellular environment. These compounds are thus suitable to serve as chemical probes to elucidate the effect of selective JAK3 inhibition. They might be used for studying the JAK1/3 interplay with particular regard to their respective roles in cytokine signaling and the resultant clinically relevant immunosuppressive effects.

EXPERIMENTAL PROCEDURES

Chemical Synthesis

Synthetic schemes and detailed synthetic procedures are described in the Supplemental Experimental Procedures.

JAK3 ELISA

For initial JAK3 IC_{50} determination, different concentrations of inhibitors were incubated in substrate-coated 96-well plates with ATP and recombinant JAK3 kinase domain (amino acids 781–1124). The degree of phosphorylation was determined by detection via monoclonal anti-pTyr-HRP-conjugated antibodies, followed by a color reaction. See the Supplemental Experimental Procedures for details and Bauer et al. (2014).

Protein Expression, Purification, Crystallization, and Structure Determination

Recombinant JAK3 kinase domain with a tobacco etch virus (TEV)-cleavable His tag was expressed in Sf9 cells. The cells were lysed and protein was initially purified by Ni-affinity chromatography. Protein was incubated overnight with the inhibitors and TEV protease. The cleaved protein was further purified by reverse Ni-affinity and size exclusion chromatography. JAK3-inhibitor complexes were crystallized using sitting-drop vapor diffusion. The obtained crystals were cryoprotected and diffraction data were collected at Diamond Light Source. More details on the procedures and crystallographic data refinement are described in the Supplemental Experimental Procedures.

BRET Experiments

Dose-response experiments were conducted in HeLa cells expressing NanoLuc JAK3 or the studied mutants using Promega tracer 5. Binding kinetic experiments in living cells after compound washout were conducted using the same constructs and tracers as described in Robers et al. (2015). A detailed description is provided in the Supplemental Experimental Procedures.

CD4+ T Cell Cytokine Stimulation Assays

T cells were purified from peripheral blood mononuclear cells from human donors. Equal numbers of cells were incubated for 1 hr with JAK inhibitors or DMSO control and stimulated with cytokines for 30 min. The cells were lysed, and the proteins were separated via PAGE and transferred to a polyvinylidene fluoride membrane. The proteins of interest were blotted with specific antibodies and visualized with an infrared imaging system. A detailed description can be found in the Supplemental Experimental Procedures.

Please cite this article as: Forster et al., Selective JAK3 Inhibitors with a Covalent Reversible Binding Mode Targeting a New Induced Fit Binding Pocket, Cell Chemical Biology (2016), http://dx.doi.org/10.1016/j.chembiol.2016.10.008

ACCESSION NUMBERS

The accession number for compound **4** co-crystallized with JAK3 is PDB: 5LWM. The accession number for compound **5** co-crystallized with JAK3 is PDB: 5LWN.

SUPPLEMENTAL INFORMATION

Supplemental Information includes Supplemental Experimental Procedures, two figures, and six tables and can be found with this article online at http://dx.doi.org/10.1016/j.chembiol.2016.10.008.

AUTHOR CONTRIBUTIONS

S.A.L. and S.K. initiated and supervised this study. M.F. and M.G. conceived the chemical experiments and M.F. carried them out. A.C. conceived the protein X-ray experiments and carried them out. S.M.B. conceived the experiments for the initial biological evaluation and carried them out. M.B.R. and C.R.C. conceived and carried out the BRET experiments. J.H. and K.G. conceived the $CD4^+$ T cell experiments and J.H. carried them out. M.F., A.C., M.G., S.M.B., and J.H. carried out the data analysis. M.F., M.G., S.M.B., E.P., A.C., K.G., and S.K. wrote the paper.

ACKNOWLEDGMENTS

K.G. would like to thank the DFG for funding (Transregio SFB TR156). S.K. is grateful for support by the SGC, a registered charity (number 1097737) receiving funds from AbbVie, Bayer Pharma AG, Boehringer Ingelheim, Canada Foundation for Innovation, Eshelman Institute for Innovation, Genome Canada through Ontario Genomics Institute, Innovative Medicines Initiative (EU/EFPIA) (ULTRA-DD grant no. 115766), Janssen, gs9:Merck, Novartis Pharma AG, Ontario Ministry of Economic Development and Innovation, Pfizer, São Paulo Research Foundation-FAPESP, Takeda, and the Wellcome Trust (grant no. 092809/Z/10/Z) as well as the Innovative Medicines Initiative (EU/EFPIA) (grant K4DD).

Received: April 6, 2016
Revised: July 21, 2016
Accepted: October 13, 2016
Published: November 10, 2016

REFERENCES

Ahearn, S.P., Christopher, M., Jung, J., Pu, Q., Rivkin, A., Scott, M.E., Witter, D.J., Woo, H.C., Cash, B., Dinsmore, C., et al. (2013). Pyrrolopyrimidines as Janus Kinase Inhibitors. WO Patent 2013/085802. https://www.google.com/patents/WO2013085802A1?cl=enIt.

Bauer, S.M., Gehringer, M., and Laufer, S.A. (2014). A direct enzyme-linked immunosorbent assay (ELISA) for the quantitative evaluation of Janus Kinase 3 (JAK3) inhibitors. Anal. Methods *6*, 8817–8822.

Chrencik, J.E., Patny, A., Leung, I.K., Korniski, B., Emmons, T.L., Hall, T., Weinberg, R.A., Gormley, J.A., Williams, J.M., Day, J.E., et al. (2010). Structural and thermodynamic characterization of the TYK2 and JAK3 kinase domains in complex with CP-690550 and CMP-6. J. Mol. Biol. *400*, 413–433.

Flanagan, M.E., Blumenkopf, T.A., Brissette, W.H., Brown, M.F., Casavant, J.M., Poa, C.S., Doty, J.L., Elliott, E.A., Fisher, M.B., Hines, M., et al. (2010). Discovery of CP-690'550: a potent and selective Janus Kinase (JAK) inhibitor for the treatment of autoimmune diseases and organ transplant rejection. J. Med. Chem. *53*, 8468–8484.

Gehringer, M., Pfaffenrot, E., Bauer, S., and Laufer, S.A. (2014). Design and synthesis of tricyclic JAK3 inhibitors with picomolar affinities as novel molecular probes. ChemMedChem *9*, 277–281.

Ghoreschi, K., Laurence, A., and O'Shea, J.J. (2009). Selectivity and therapeutic inhibition of kinases: to be or not to be? Nat. Immunol. *10*, 356–360.

Ghoreschi, K., Jesson, M.I., Li, X., Lee, J.L., Ghosh, S., Alsup, J.W., Warner, J.D., Tanaka, M., Steward-Tharp, S.M., Gadina, M., et al. (2011). Modulation of innate and adaptive immune responses by tofacitinib (CP-690,550). J. Immunol. *186*, 4234–4243.

Goedken, E.R., Argiriadi, M.A., Banach, D.L., Fiamengo, B.A., Foley, S.E., Frank, K.E., George, J.S., Harris, C.M., Hobson, A.D., Ihle, D.C., et al. (2015). Tricyclic covalent inhibitors selectively target Jak3 through an active site thiol. J. Biol. Chem. *290*, 4573–4589.

Haan, C., Rolvering, C., Raulf, F., Kapp, M., Drückes, P., Thoma, G., Behrmann, I., and Zerwes, H.-G. (2011). Jak1 has a dominant role over jak3 in signal transduction through gamma-c-containing cytokine receptors. Chem. Biol. *18*, 314–323.

Knapp, S., Arruda, P., Blagg, J., Burley, S., Drewry, D.H., Edwards, A., Fabbro, D., Gillespie, P., Gray, N.S., Kuster, B., et al. (2013). A public-private partnership to unlock the untargeted kinome. Nat. Chem. Biol. *9*, 3–6.

Liu, Q., Sabnis, Y., Zhao, Z., Zhang, T., Buhrlage, S.J., Jones, L.H., and Gray, N.S. (2013). Developing irreversible inhibitors of the protein kinase cysteinome. Chem. Biol. *20*, 146–159.

London, N., Miller, R.M., Krishnan, S., Uchida, K., Irwin, J.J., Eidam, O., Gibold, L., Cimermančič, P., Bonnet, R., Shoichet, B.K., et al. (2014). Covalent docking of large libraries for the discovery of chemical probes. Nat. Chem. Biol. *10*, 1066–1072.

Muller, S., Chaikuad, A., Gray, N.S., and Knapp, S. (2015). The ins and outs of selective kinase inhibitor development. Nat. Chem. Biol. *11*, 818–821.

Pesu, M., Candotti, F., Husa, M., Hofmann, S.R., Notarangelo, L.D., and O'Shea, J.J. (2005). Jak3, severe combined immunodeficiency, and a new class of immunosuppressive drugs. Immunol. Rev. *203*, 127–142.

Robers, M.B., Dart, M.L., Woodroofe, C.C., Zimprich, C.A., Kirkland, T.A., Machleidt, T., Kupcho, K.R., Levin, S., Hartnett, J.R., Zimmerman, K., et al. (2015). Target engagement and drug residence time can be observed in living cells with BRET. Nat. Commun. *6*, 10091.

Serafimova, I.M., Pufall, M.A., Krishnan, S., Duda, K., Cohen, M.S., Maglathlin, R.L., McFarland, J.M., Miller, R.M., Frödin, M., and Taunton, J. (2012). Reversible targeting of noncatalytic cysteines with chemically tuned electrophiles. Nat. Chem. Biol. *8*, 471–476.

Singh, J., Petter, R.C., and Kluge, A.F. (2010). Targeted covalent drugs of the kinase family. Curr. Opin. Chem. Biol. *14*, 475–480.

Smith, G.A., Uchida, K., Weiss, A., and Taunton, J. (2016). Essential biphasic role for JAK3 catalytic activity in IL-2 receptor signaling. Nat. Chem. Biol. *12*, 373–379.

Tan, L., Akahane, K., McNally, R., Reyskens, K.M.S.E., Ficarro, S.B., Liu, S., Herter-Sprie, G.S., Koyama, S., Pattison, M.J., Labella, K., et al. (2015). Development of selective covalent Janus Kinase 3 inhibitors. J. Med. Chem. *58*, 6589–6606.

Thoma, G., Nuninger, F., Falchetto, R., Hermes, E., Tavares, G.A., Vangrevelinghe, E., and Zerwes, H.-G. (2011). Identification of a potent Janus kinase 3 inhibitor with high selectivity within the Janus kinase family. J. Med. Chem. *54*, 284–288.

Thoma, G., Drückes, P., and Zerwes, H.-G. (2014). Selective inhibitors of the Janus kinase Jak3—Are they effective? Bioorg. Med. Chem. Lett. *24*, 4617–4621.

Thorarensen, A., Banker, M.E., Fensome, A., Telliez, J.-B., Juba, B., Vincent, F., Czerwinski, R.M., and Casimiro-Garcia, A. (2014). ATP-mediated kinome selectivity: the missing link in understanding the contribution of individual jak kinase isoforms to cellular signaling. ACS Chem. Biol. *9*, 1552–1558.

Cell Chemical Biology, Volume *23*

Supplemental Information

Selective JAK3 Inhibitors with a Covalent Reversible Binding Mode Targeting a New Induced Fit Binding Pocket

Michael Forster, Apirat Chaikuad, Silke M. Bauer, Julia Holstein, Matthew B. Robers, Cesear R. Corona, Matthias Gehringer, Ellen Pfaffenrot, Kamran Ghoreschi, Stefan Knapp, and Stefan A. Laufer

Supplemental Data Items

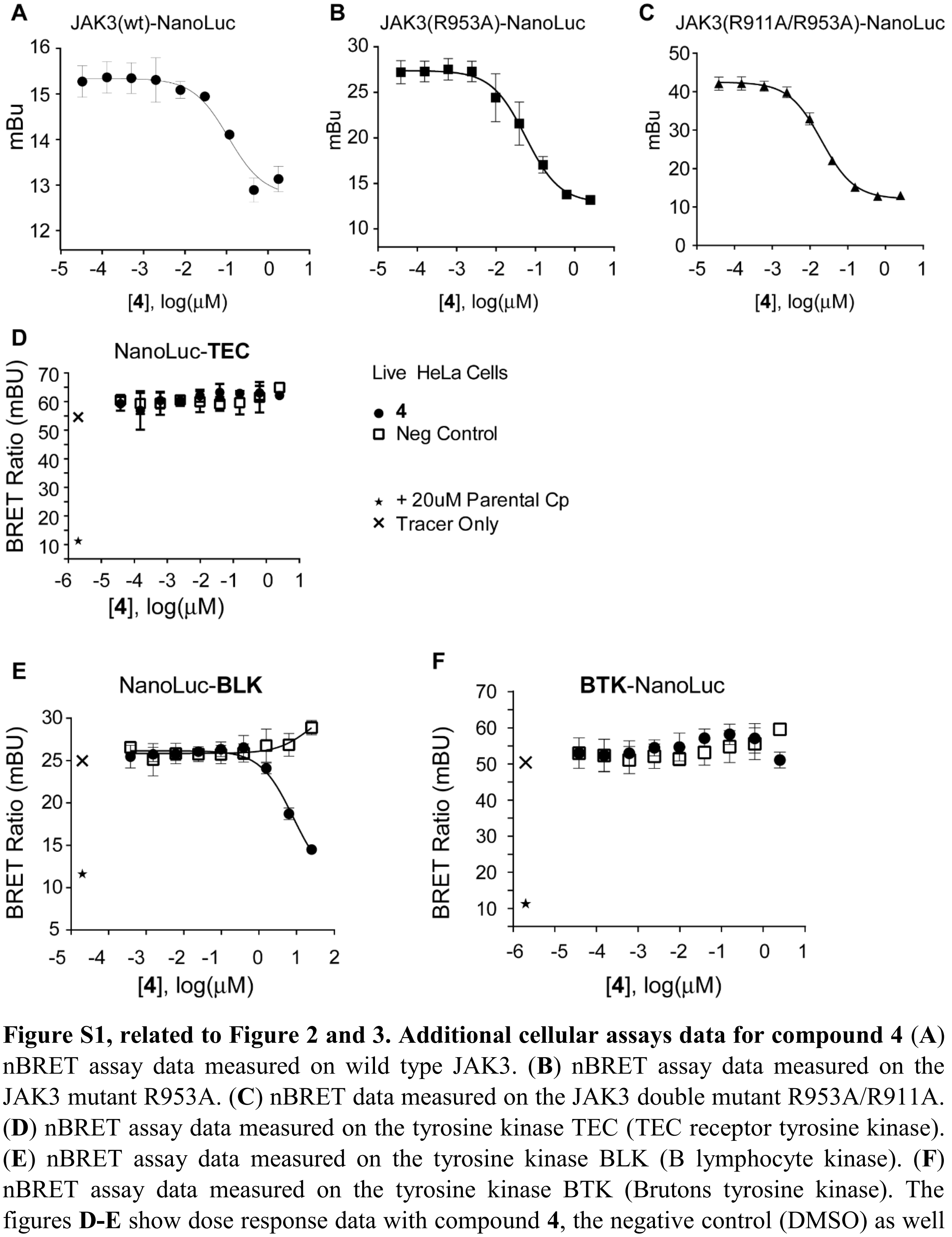

Figure S1, related to Figure 2 and 3. Additional cellular assays data for compound 4 (**A**) nBRET assay data measured on wild type JAK3. (**B**) nBRET assay data measured on the JAK3 mutant R953A. (**C**) nBRET data measured on the JAK3 double mutant R953A/R911A. (**D**) nBRET assay data measured on the tyrosine kinase TEC (TEC receptor tyrosine kinase). (**E**) nBRET assay data measured on the tyrosine kinase BLK (B lymphocyte kinase). (**F**) nBRET assay data measured on the tyrosine kinase BTK (Brutons tyrosine kinase). The figures **D-E** show dose response data with compound **4**, the negative control (DMSO) as well as displacement by the tracer parental compound (*) and the tracer alone (x). All experiments show average data of four experiments as well as the standard deviation for each data point.

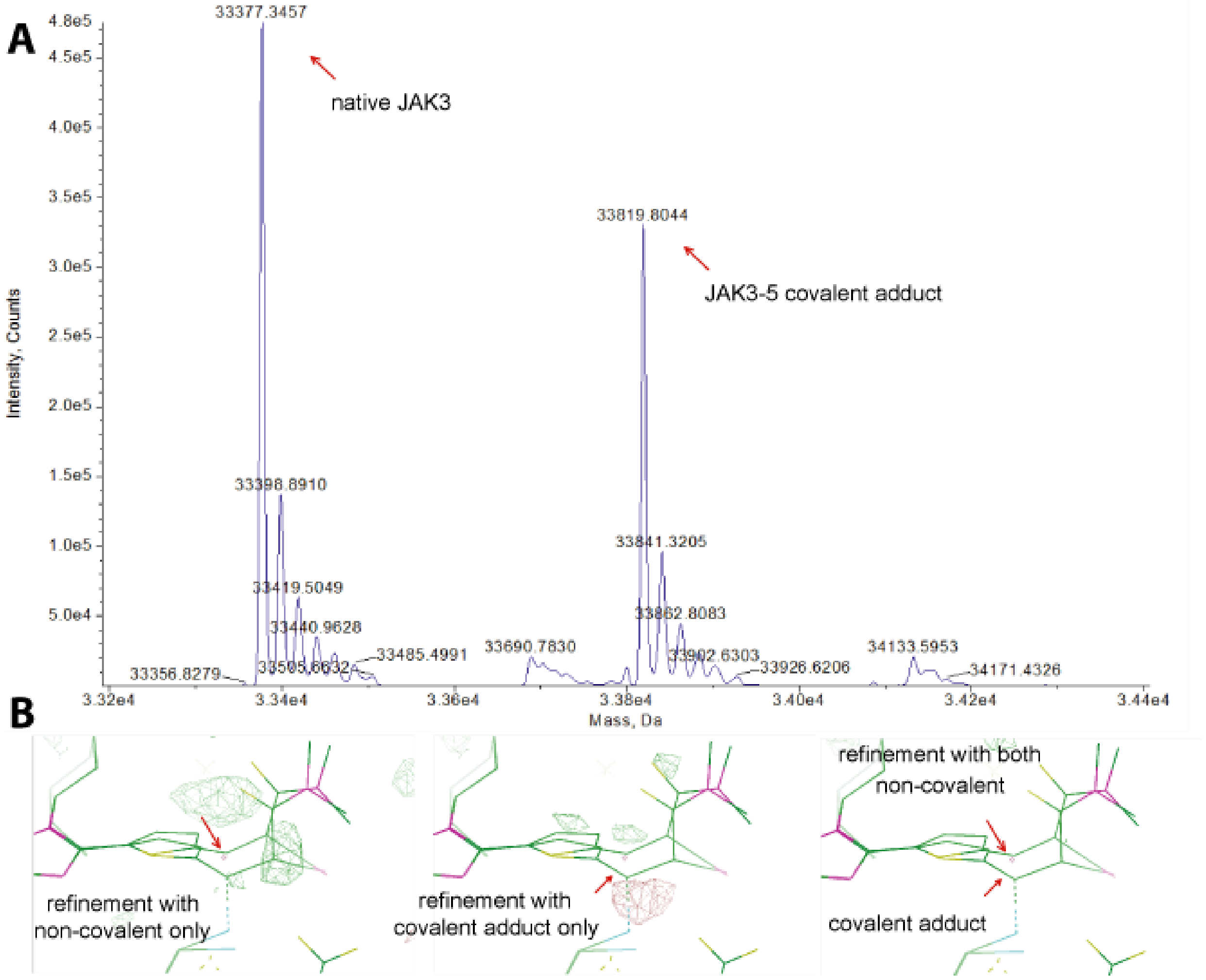

Figure S2, related to Figure 2. Additional data on the binding mode of 5 to JAK3. (**A**) ESI-TOF-MS spectra of JAK3 treated overnight with compound **5** at three-fold excess at 4 ºC. The covalent binding mode of the inhibitor is evident by the mass shift of the protein. (**B**) FoFc difference density maps showing either positive (green) (additional density not accounted for by the model) as well as negative (red) electron density (surplus density created by incorrectly placed atoms of the model) investigating the covalent as well as non-covalent binding modes of compound **5**.

Table S1, related to Figure 1 and 4. Initial JAK3 inhibition ELISAs and JAK isoform selectivity assays at the isolated enzymes.

Compd.	JAK3 IC_{50} [nM][a]	IC_{50} [nM][b] JAK1	JAK2	JAK3	TYK2
**1**	3.5 ± 0.6	0.496	2.2	0.292	8.9
**2**	63 ± 9	-	-	-	-
**3**	51 ± 6	19	113	22	78
**4**	9 ± 1	52	346	0.127	459
**5**	17 ± 1	64	270	0.154	898
**6**	470 ± 36	-	-	-	-
**7**	548 ± 39	-	-	-	-
**8**	226 ± 31	-	-	-	-

[a]IC_{50} values are calculated from the results of a ELISA (Bauer et al., 2014). Data represent the average ± SEM (n = 3) for **2-8** and average ± SD (n = 18) for **1**. [b]IC_{50} values were calculated from the results of a radiometric assay. Data were obtained as 5-dose singlicate IC_{50} value with 10-fold serial dilution starting at 1 μM. [ATP] = 10 μM. Tofacitinib citrate (**1**) was purchased from Shanghai BOC Chemical Co., Ltd.

Table S2, related to Figure 1. Kinome selectivity of 4 and 5, see additional excel file.

Table S3, related to Figure 1. Kinetic profiling of Compound 4

kinase	K_D [μM]	k_{on} [1/s 1/M][a]	k_{off} [1/s][a]	residence time [min][b]
**JAK1**	6.97E-02	n.a.	n.a.	< 1.4
**JAK2**	2.42E-01	n.a.	n.a.	< 1.4
**JAK3**	1.34E-03	2.47E+05	3.30E-04	50
**TYK2**	3.60E-01	n.a.	n.a.	< 1.4

[a]n.a. = below detection limit; [b]< 1.4 = lower detection limit; Data was obtained from a commercial displacement assay. Displacement of a reporter probe was measured time dependent for different concentrations of the tested compound. Detailed methodology are described in the Supplemental Experimental Procedures.

Table S4, related to Figures 2 and 3. Xray data collection and refinement

	**4**-JAK3	**5**-JAK3
**Data collection**		
Space group	$P\ 2_1$	$P\ 2_1$
Cell dimensions		
a, b, c (Å)	42.0, 62.5, 51.3	42.0, 62.3, 51.1
α, β, γ (°)	90.0, 92.9, 90.0	90.0, 92.6, 90.0
Resolution (Å)	39.60-1.55 (1.63-1.55)*	62.57-1.60 (1.69-1.60)
R_{merge}	0.073 (0.722)	0.082 (0.680)
I /σI	11.3 (2.7)	9.9 (2.2)
Completeness (%)	99.7 (99.4)	100.0 (100.0)
Redundancy	5.9 (5.6)	6.0 (6.0)
**Refinement**		
PDB-ID:	5LWM	5LWN
Resolution (Å)	39.60-1.55 (1.63-1.55)*	62.57-1.60 (1.69-1.60)
No. reflections	38,357 (5,553)	35,006 (5,106)
R_{work} / R_{free}	0.163/ 0.204	0.166/ 0.209
No. atoms		
Protein	2,333	2,352
Compounds (4 or 5)	32	66
Water and solvents	247	228
B factors (Å^2)		
Protein	27	30
Compounds (4 or 5)	25	24
Water and solvents	37	38
r.m.s. deviations		
Bond lengths (Å)	1.6	1.6
Bond angles (°)	0.016	0.016

*Values in parentheses are for highest-resolution shell.

Table S5, related to Figure 3. Amino acid setup involved in formation of the arginine-pocket

Kinases with equivalent cysteine to Cys909 in JAK3

Kinase	UNIPROT Entry #	begin ATP binding site	reactive cysteine	Arginine-pocket residues		
**JAK3**	P52333	Leu828	Cys909	Arg911	Asp912	Arg953
**MAP2K7** (MKK7)	O14733	Met126	Cys202	Glu204	Lys205	Pro246
**TEC**[a]	P42680	Leu376	Cys449	Leu451	Asp452	Arg493
**TXK**[a]	P42681	Ile277	Cys350	Leu352	Asn353	Arg494
**ITK**	Q08881	Ile369	Cys402	Ser444	Asp445	Arg486
**BTK**	Q06187	Leu407	Cys481	Leu483	Asn484	Arg925
**BMX**	P51813	Leu423	Cys496	Leu498	Asn499	Arg540
**BLK**[a]	P51451	Leu246	Cys319	Leu321	Asp322	Arg362
**HER2** (ERBB2)	P04626	Leu726	Cys805	Leu807	Asp808	Arg849
**EGFR**	P00533	Leu718	Cys797	Leu799	Asp800	Arg841
**HER4** (ERBB4)	Q15303	Leu724	Cys803	Leu805	Glu806	Arg847

JAK-Family

Kinase	UNIPROT Entry #	begin ATP binding site	reactive cysteine	Arginine-pocket residues		
**JAK1**	P23458	Leu881	Ser963	Lys965	Glu966	Arg1007
**JAK2**	O60674	Leu855	Ser936	Arg938	Asp939	Arg980
**JAK3**	P52333	Leu828	Cys909	Arg911	Asp912	Arg953
**TYK2**	P29597	Leu903	Ser985	Arg987	Asp988	Arg1027

The reactive cysteine is usually located around 70 positions in C-terminal direction from the begin of the ATP-binding site. Arg911 and Asp912 positions follow 2 and 3 amino acids later. The position of Arg953 is usually situated around 40 positions later.

[a]for these kinases no PDB structure was available (february 2016)

Table S6, related to Figure 1. Comparison of 4 and 5 with other literature known JAK inhibitors

entry	Compound	JAK3 IC_{50} [nM]	Selectivity over JAK1/JAK2	Selectivity over TYK2	Kinom Selectivity
**1**	**Compound 4 and 5** (Forster *et al.*)	0.127	410 / 2724 fold<br>412 / 1747 fold<br>respectively	3614 fold<br>5820 fold<br>respectively	**410 kinases tested**<br>**@ 100 nM**<br>**no** off targets<br>**@ 500 nM**<br>11 or 1 off target<br>for **4** or **5**, respectively
**2**	**NIBR3049**<br>Novartis<br>**(Thoma et al., 2011)**	1.5	105 / 94 fold	1400 fold	**40 kinases tested**<br>PKC and GSK3β<br>IC_{50} < 1 µM
**3**	**WYE-151650**<br>Wyeth<br>**(Thoma et al., 2014)**	0.9	36 / 14 fold	35 fold	>100-fold selectivity against a panel of 27 non-JAK kinases (data not shown)
**4**	**CP-690,550**<br>Pfizer<br>**(Davis et al., 2011)**	8	1.5 / 0.8 fold	22 fold	**442 kinases tested**<br>Kd's (< 1 µM)<br>12 nM DCAMKL3<br>460 nM LCK<br>550 nM LTK<br>170 nM PKN1<br>470 nM ROCK1<br>420 nM ROCK2<br>600 nM RSK3<br>549 nM RSK4<br>240 nM SNARK<br>120 nM TNK1
**5**	**Compound 3q**<br>Roche<br>**(Soth et al., 2013)**	0.3	12 /3 fold	n.d.	**48 kinases tested**<br>% inhibition @ 1 µM<br>AMPK 67%<br>AurA 78%<br>FGFR1 63%<br>MARK1 67%
**6**	**Cmpd 2**<br>AbbVie<br>**(Goedken et al., 2015)**	7	1600 / 700 fold	> 7000 fold	**n.d.**
**7**	**Cmpd 3**<br>AbbVie<br>**(Goedken et al., 2015)**	3	2000 / 500 fold	> 7000 fold	**78 kinases tested**<br>**trFRET binding assay**<br>**8 kinases IC_{50} < 10 µM**<br>BTK 1.25 µM<br>FGFR1 3.1 µM<br>GRK5 9.4 µM<br>JAK3 0.002 µM<br>LTK 5.6 µM<br>MAP4K2 7.4 µM<br>RET 7.8 µM<br>STK33 7.1 µM

**8**	**Cmpd 4**<br>**(Goedken et al., 2015)**<br>(patented by Cytopia)	20	> 2500 / 2400 fold	300 fold	**78 kinases tested**<br>**trFRET binding assay**<br>**19 kinases IC_{50} < 1-10 μM**<br>**7 kinases IC_{50} < 1 μM**<br>BTK 0.64 μM<br>FLT1 0.55 μM<br>FYN 0.66 μM<br>JAK3 0.3 μM<br>KDR 0.55 μM<br>SRC 0.98 μM<br>STK16 1.0 μM
**9**	**Cmpd 9**<br>Gray Laboratory<br>**(Tan et al., 2015)**	4.8	190 / 220 fold	> 2000 fold	**456 kinases tested**<br>@ 1μM residual activity against:<br>FLT3 0.6 %<br>TTK 1.4 %<br>BLK 1.6 %<br>TXK 3.4 %<br>FAK 4 %<br>CAMKK2 4.2 %<br>GAK 5.4 %<br>LTK 5.9 %<br>ERBB4 6 %<br>SGK 6.8 %<br>ARK5 9.6 %<br>PDGFRB 9.6 %<br>TNK 9.9 %
**10**	**Cmpd 45**<br>Gray Laboratory<br>**(Tan et al., 2015)**	< 0.5	> 70 / > 100 fold	> 800 fold	**456 kinases tested**<br>@ 100 nM residual activity against:<br>AURKA 2.7 %<br>FLT3 10 %
**11**	**JAK3i**<br>**(Smith et al., 2016)**<br>(patented by Merck)	0.43	3500 / 20 000 fold	> 20 000 fold	**Three Cys909-equivalent-kinases tested**<br>1300 fold over EGFR<br>600 fold over ITK<br>50 fold over BTK
**12**	**Compound 31**<br>**(London et al., 2014)**	49	> 200 fold	> 200 fold	**Nine Cys909-equivalent kinases tested:**<br>BLK 0.022 μM<br>BMX 2.41 μM<br>BTK 1.64 μM<br>EGFR > 10 μM<br>ERB-B2 2.05 μM<br>ERB-B4 0.044 μM<br>ITK 0.221 μM<br>TEC 6.93 μM<br>TXK 1.67 μM

Supplemental Experimental Procedures

JAK3 ELISA

Initial JAK3 IC_{50} determination of the synthesized inhibitors was performed as described previously by (Bauer et al., 2014). In this ELISA-based kinase activity assay, a 96-well assay plate is coated with an artificial polypeptide serving as kinase substrate that contains tyrosine residues being phosphorylated by JAK3 in presence of ATP. For the kinase reaction, the recombinant active human JAK3 protein fragment (aa 781 to 1124) is incubated with different concentrations of the inhibitor candidate and an appropriate amount of ATP (twice the Km value). As a consequence, the polypeptidic kinase substrate is phosphorylated as a function of inhibitory potency of the test compound. Phosphorylated tyrosine residues are detected via a monoclonal phopho-tyrosine HRP- conjugated antibody and quantification of phosphorylation degree is perfomed by comparing the phosphorylation achieved in absence (positive control) and presence of inhibitor candidate.

$CD4^+$ T Cell Experiments

$CD4^+$ T cells were purified from peripheral blood mononuclear cells from human donors by magnetic cell separation technology (Miltenyi Biotec), activated with plate-bound anti-CD3 (clone UCHT1) and anti-CD28 (clone CD28.2) antibodies (each 5 µg/ml, Biolegend) in X-VIVO 15 medium (Lonza) for 3 days and expanded with rhIL-2 for another 6 days. After expansion T cells were washed and rested in fresh medium overnight. Equal numbers of T cells were incubated with the indicated concentrations of the JAK inhibitors **1**, **4** or **5** in DMSO or DMSO alone (control) for 1 hour and then stimulated with either rhIL-2 (50 ng/ml; Proleukin, Novartis), rhIL-6 (50 ng/ml; Peprotech), rhIL-4 (50 ng/ml, Peprotech) or IFN-α (1000 U/ml, Roche) for 30 min. After stimulation, cells were lysed in Triton X-100 lysis buffer containing protease and phosphatase inhibitors. Equal amounts of total protein were separated by PAGE, transferred to PVDF membrane, and blotted with Abs recognizing actin (Merck Millipore), specific phospho-STAT antibodies (anti-phospho-Stat5 Tyr694, anti-phospho-Stat3 Tyr705, both Cell Signaling) and IRDye-labelled secondary antibodies (680RD, 800CW, both LI-COR Biosciences) for detection. Specific bands were visualized using an Odyssey infrared imaging system (LI-COR Biosciences).

Protein production, crystallization and structure determination.

Recombinant triple-mutated JAK3 (aa 812-1103, D949A/C1040S/C1048S) containing an N-terminal TEV-cleavable His-tag was expressed in Sf9 cells. The cells were harvested after 48-

52 hours after postinfection with 10 ml/L virus, and were resuspended in 50 mM Tris, pH 8.0, 500 mM NaCl, 5% glycerol, 1 mM TCEP and 5 mM imidazole. Cells were lysed by sonication, and the protein was initially purified by Ni-affinity chromatography. The eluted protein was incubated overnight at 4 ˚C with the compounds at 3-fold molar excess in a presence of TEV protease and an addition of 1 mM TCEP. The cleaved protein was subjected to a reverse Ni-affinity purification, and was purified further by size exclusion chromatography in a buffer containing 20 mM Tris, pH 8.0, 250 mM NaCl, 10 mM DTT and 10% glycerol. The protein was concentrated to 15 mg/ml, and N-phenylurea was added to 0.26%. Mass spectrometry analyses showed that there was a mixture of unmodified JAK3 and covalent adduct of JAK3-inhibitor complex, and this could likely be due to high concentration of reducing agent throughout the purification. JAK3-inhibitor complex at 11-13 mg/ml was crystallized using sitting drop vapour diffusion at 4 ˚C and the condition containing 18-25% PEG 3350, 0.1-0.2 M $MgCl_2$ and 0.1 M MES, pH 5.5-6.1. Crystals were cryoprotected in the reservoir solution supplemented with 22% ethylene glycol. Diffraction data collected at Diamond Light Source were processed with MOSFLM (Powell et al., 2013) and subsequently scaled with Scala (Evans, 2006). Structures were determined using Phaser (McCoy et al., 2007) and the published coordinates of JAK3 (Goedken et al., 2015). Model building alternated with structure refinement was performed in COOT (Emsley et al., 2010) and Refmac (Murshudov et al., 2011), respectively. The final models were verified for their geometric correctness with MOLPROBITY (Chen et al., 2010). The data collection and refinement statistics are summarized in Supplementary Table S4.

Proteros Reporter Displacement Assay

The assay is based on the displacement of a reporter probe by a test compound. Close proximity of the probe and the targeted kinase result in emission of an optical signal. Binding of a competitive test compound to the kinase (displacement of the reporter probe) results in a diminished assay signal. The displacement of the reporter probe is measured over time for eighteen different compound concentrations in two-fold dilution steps starting from 9.8 µM. The measured signal for the lowest compound concentration represents full probe binding while the signal of highest compound concentration represents full probe displacement. For K_d determination the calculated percentages of probe displacement are plotted against compound concentration for the last time point, at which the system reached equilibrium. An IC_{50}-like value, corresponding to 50% probe displacement, is determined by standard fitting

algorithms. According to the Cheng-Prusoff-equation the K_d value is calculated as $K_d = 0.5 \times IC50$.

To determine the kinetic constants (k_{on}, k_{off} and residence time τ) reporter displacement was plotted against time for each concentration and fitted to a mono-exponential decay equation (probe binding = B + A x exp(-k_{obs} x t)). The exponential coefficients represent the apparent association rate k_{obs}. These association rates were plotted against the compound concentration in a secondary plot. Following the equation $k_{obs} = k_{off} + k_{on} \times [cmpd]$ the kon values were determined as the slope of the curve after linear fitting. Off rates are then calculated as $k_{off} = K_d \times k_{on}$ and residence time as $\tau = 1/k_{off}$. For these secondary plots only data was used from compound concentrations at which the apparent association rate k_{obs} could clearly be determined. Examplpes and more details on this method is provided in the reference by (Neumann et al., 2011).

Cell transfection, treatments, and BRET measurements in living cells

The BRET methodology relies on the emission of an optical signal dependent of the spatial proximity of the luciferase-conjugated target protein and a fluorescent-labelled tracer molecule. The displacement of the tracer by a competitive inhibitor therefore diminishes the apparent BRET signal (Machleidt et al., 2015).

Full-length JAK3 and BTK ORFs were subloned into pFC-32K Nluc-CMV Neo (Promega) to generate a C-terminal placement of NanoLuc and BLK and TEC kinase ORFs were subcloned into pFN-31K Nluc-CMV Neo (Promega) to generate an N-terminal placement of NanoLuc. To lower intracellular expression levels of the reporter fusion, each NanoLuc/kinase fusion construct was diluted into Transfection Carrier DNA (Promega) at a mass ratio of 1:10 (mass/mass), prior to forming FuGENE HD complexes according to the manufacturer's protocol (Promega). DNA:FuGENE complexes were formed at a ratio of 1:3 (µg DNA/µL FuGENE). 1 part of the transfection complexes was then mixed with 20 parts (v/v) of HeLa cells (ATCC) suspended at a density of 2×10^5 /mL in DMEM (Gibco) + 10% FBS (GE Healthcare), seeded into T75 flasks and allowed to express for 20 h. Cells were then trypsinized, resuspended in Opti-MEM without phenol red (Life Technologies) and reseeded into white 96-well plates (Corning 4600) at a density of 2×10^4 cells per well. For target engagement, NanoBRET Kinase Tracer-05 (Promega) was added to the cells at 1 µM prior to test compound addition. Serially-diluted test compounds were then added to the cells and allowed to equilibrate for 2 hr at 37°C / 5% CO2 prior to BRET measurements. For real-time analysis of compound dissociation, the transfected cells were first treated in 15 mL

conical tubes (Corning) with a near-saturating dose (1 □M) of compound for 2 hr in Opti-MEM (Life Technologies). Cells were centrifuged and washed to remove unbound compound prior to immediate addition of NanoBRET Kinase Tracer-05 at 1 □M. To measure BRET in live cells, Intracellular TE Nano-Glo Substrate/Inhibitor (Promega) was added according to the manufacturer's protocol, and filtered luminescence was measured at a single timepoint or in real-time via repeat measurements on a GloMax Discover luminometer equipped with 450 nm BP filter (donor) and 610 nm LP filter (acceptor), using 0.5 s integration time. Milli-BRET units (mBU) are the BRET values x 1000. Competitive displacement data were then graphed with GraphPad Prism software using a 3-parameter curve fit with the following equation:

$$Y = Bottom + (Top - Bottom)/(1 + 10^{\wedge}((X - LogIC_{50})))$$

Further details on tracer based BRET assays included examples of tracer structures are provided in (Robers et al., 2015).

Chemical Synthesis

General: Reagents, starting materials and solvents were of commercial quality and were used without further purification unless otherwise stated. TLC analysis was carried out on Merck 60 F_{254} silica gel plates and visualized under UV light at 254 nm and 365 nm. Preparative column chromatography was carried out on Grace Davison Davisil LC60A 20-45 micron or Merck Geduran Si60 63-200 micron silica using a Interchim PuriFlash 430 automated flash chromatography system. The purity of final compounds was determined via RP-HPLC on a Hewlett Packard 1090 Series II LC with a Phenomenex Luna C8 column (150 x 4.6 mm, 5 µm) and detection was performed by a UV DAD at 254 nm and 230 nm wavelength. Elution was carried out with the following gradient: 0.01 M KH_2PO_4, pH 2.30 (solvent A), MeOH (solvent B), 40 % B to 85 % B in 8 min, 85 % B for 5 min, 85 % to 40 % B in 1 min, 40 % B for 2 min, stop time 16 min, flow 1.5 ml/min. NMR spectra were recorded on a Bruker Avance 200 or Bruker Avance 400 NMR spectrometer. Chemical shifts are reported in ppm relative to TMS and the spectra were calibrated against the residual proton peak of the used deuterated solvent. Standard mass spectra were obtained either as ESI-MS (pos. and/or neg. mode) from a Advion DCMS interface, (settings as follows: ESI voltage 3,50 kV, capillary voltage 187 V, source voltage 44 V, capillary temperature 250 °C, desolvation gas temperature 250 °C, gas flow 5 l/min) or as FAB-MS (pos. and/or neg. mode) measured by the mass spectrometry department, Institute of Organic Chemistry, Eberhard-Karls-University Tuebingen. HRMS for final compounds was performed on an Sciex TripleTof 5600+ mass spectrometer with an Duospray source, coupled to a 1290 UHPLC from Agilent equipped with an PAL-HTS Autosampler from CTC. Preliminary chromatographic separation was performed on a Phenomenex Kinetex C18 2.8µ 100x3mm 100Å coreshell technology column using the following gradient: H_2O with 0.1 % formic acid (solvent A), Acetonitrile with 0.1% formic acid (solvent B) 0-1min 5 % B, 1-10min 5 to 100 % B, 5-15 100 % B. Flow 0.5 ml/min, column temperature 20°C. Mass analysis was run in ESI+ Mode, settings were as follows: curtain gas 30 psi, nebulizer gas 40 psi, drying gas 50 psi, source temperature 400 °C, ion source floating voltage +5500 V. Analysis was run as information dependent acquisition, i.e. every cycle consisted of a TOF survey scan from 100 to 2000 m/z and subsequent fragmentation scans were triggered automatically by acquisition software. To ensure MSMS spectra generation of synthesized compounds, IDA was supplied with a inclusion list containing the respective $[M+H]^+$ m/z ratios. Collision energy was set to 27 V.

Synthetic Procedures

Preparation of Starting Materials and Reagents

Hydroxymethyl benzaldehydes

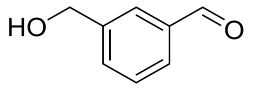

9a

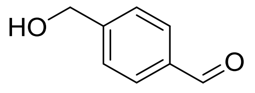

9b

*3-(hydroxymethyl)benzaldehyde (**9a**).* Isophthalic aldehyde (1.0 g, 7.46 mmol) was suspendend in 5 ml THF and 10 ml EtOH and the suspension was cooled with an ice/water bath. $NaBH_4$ granules (100 mg, 2.64 mmol) were added in several portions (dissolves slowly) and the reaction was monitored by TLC (PE/EA 1:1). About 15 min after complete addition starting material was consumed and the reaction was quenched by addition of 5 ml HCl_{aq} (10 %). The mixture was diluted with water and extracted with EA (3x20 ml). The combined extracts were washed two times with brine, dried over Na_2SO_4 and evaporated to yield 925 mg (91 %) of **9a** as yellowish oil. ^{1}H NMR (200 MHz, $CDCl_3$) δ 9.90 (s, 1H), 7.79 (s, 1H), 7.71 (d, *J* = 7.5 Hz, 1H), 7.57 (d, *J* = 7.5 Hz, 1H), 7.44 (t, J = 7.5 Hz, 1H), 4.69 (s, 2H), 3.30 (s, 1H). ^{13}C NMR (50 MHz, $CDCl_3$) δ 192.7, 142.2, 136.4, 133.0, 129.2, 129.0, 127.8, 64.1

*4-(hydroxymethyl)benzaldehyde (**9b**)* was prepared from terephthalic aldehyde following the same procedure as for **9a**, but afforded flash purification (PE/EA 20-80 %). Yield 653 mg (64 %) of **9b** as white solid. ^{1}H NMR (200 MHz, $CDCl_3$) δ 9.85 (s, 1H), 7.80 (d, *J* = 7.9 Hz, 2H), 7.47 (d, *J* = 7.8 Hz, 2H), 4.83 (s, 2H), 2.39 (s, 1H). ^{13}C NMR (50 MHz, $CDCl_3$) δ 191.9, 147.8, 135.6, 130.0, 127.0, 64.7

Horner-Wadsworth-Emmons-Reagents

HWE-reagents were obtained from the alkyl chlorides under Arbuzov conditions following a protocol of *(Zhang et al., 2009).*

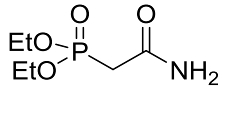

10a

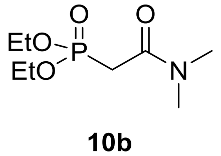

10b

*diethyl (2-amino-2-oxoethyl)phosphonate (**10a**).* Chloroacetamide (2.81 g, 30 mmol) and 15 ml triethyl phosphite were combined in a 25 ml round bottomed flask und heated to 150 °C oil bath temperature overnight. The excess triethyl phosphite was removed under reduced pressure and the residue was poured in 100 ml cold Et_2O. The suspension was stirred for

5 min, then the precipitate was collected by filtration, washed with Et_2O and dried in vacuo. 3.67 g (63 %) of the product **10a** was obtained as pale yellowish crystalline solid. ^{1}H NMR (200 MHz, DMSO) δ 7.38 (br s, 1H), 7.03 (br s, 1H), 4.02 (dq, *J* = 14.1, 7.1 Hz, 4H), 2.80 (d, *J* = 21.4 Hz, 2H), 1.22 (t, *J* = 7.1 Hz, 6H) ^{13}C NMR (50 MHz, DMSO) δ 166.0 (d, J = 5.5 Hz), 61.6 (d, *J* = 6.1 Hz), 34.5 (d, *J* = 131.3 Hz), 16.2 (d, *J* = 6.1 Hz)

2-chloro-N,N-dimethylacetamide was prepared via Schotten-Baumann procedure reported from Kem K *et al.(Kem et al., 1981)* A biphasic mixture of 50 ml DCM and 50 ml $NaOH_{aq}$ (20 %wt) was cooled to -10 °C internal temperature. Dimethylamine-HCl (4.89 g, 60 mmol) was added in one portion, followed by the dropwise addition of chloroacetyl chloride (5.65 g, 50 mmol) to the vigorously stirred mixture in a rate that the temperature kept below 0 °C. After complete addition, stirring was continued for 20 min. Then the phases were separated and the aqueous phase was extracted with DCM (3x20 ml). The combined organic phases were washed with HCl (10 %) and brine. After drying over Na_2SO_4 and evaporation of the solvent 4,52 g (72 %) of the crude dimethylacetamide was obtained as brownish oil. The product was used in the next step without further purification. ^{1}H NMR (200 MHz, $CDCl_3$) δ 4.05 (s, 2H), 3.06 (s, 3H), 2.94 (s, 3H). ^{13}C NMR (50 MHz, CDCl3) δ 166.5, 41.2, 37.6, 36.0

*diethyl (2-(dimethylamino)-2-oxoethyl)phosphonate **(10b)*** The crude 2-chloro-*N,N*-dimethylacetamide (4.39 g, 36 mmol) from the previous step and 7.2 ml triethyl phosphite were combined in a 25 ml round bottomed flask und heated to 150 °C oil bath temperature overnight. The mixture was subjected to a short-path vacuum distillation and the first 0.5-1.0 ml were discarded to remove excess triethyl phosphite. The main fraction was collected from 125-135 °C at 0.25 mbar yielding 7.38 g (92 %) of **10b** as colorless oil. ^{1}H NMR (200 MHz, $CDCl_3$) δ 4.12 (dq, *J* = 14.4, 7.0 Hz, 4H), 3.12 – 2.88 (m, 8H), 1.28 (td, *J* = 7.0, 0.5 Hz, 6H). ^{13}C NMR (50 MHz, $CDCl_3$) δ 164.9 (d, *J* = 5.4 Hz), 62.6 (d, *J* = 6.5 Hz), 38.6, 35.8, 33.4 (d, *J* = 133.5 Hz), 16.4 (d, *J* = 6.4 Hz).

Preparation of 1,6-dihydroimidazo[4,5-d]pyrrolo[2,3-b]pyridine intermediates and 2

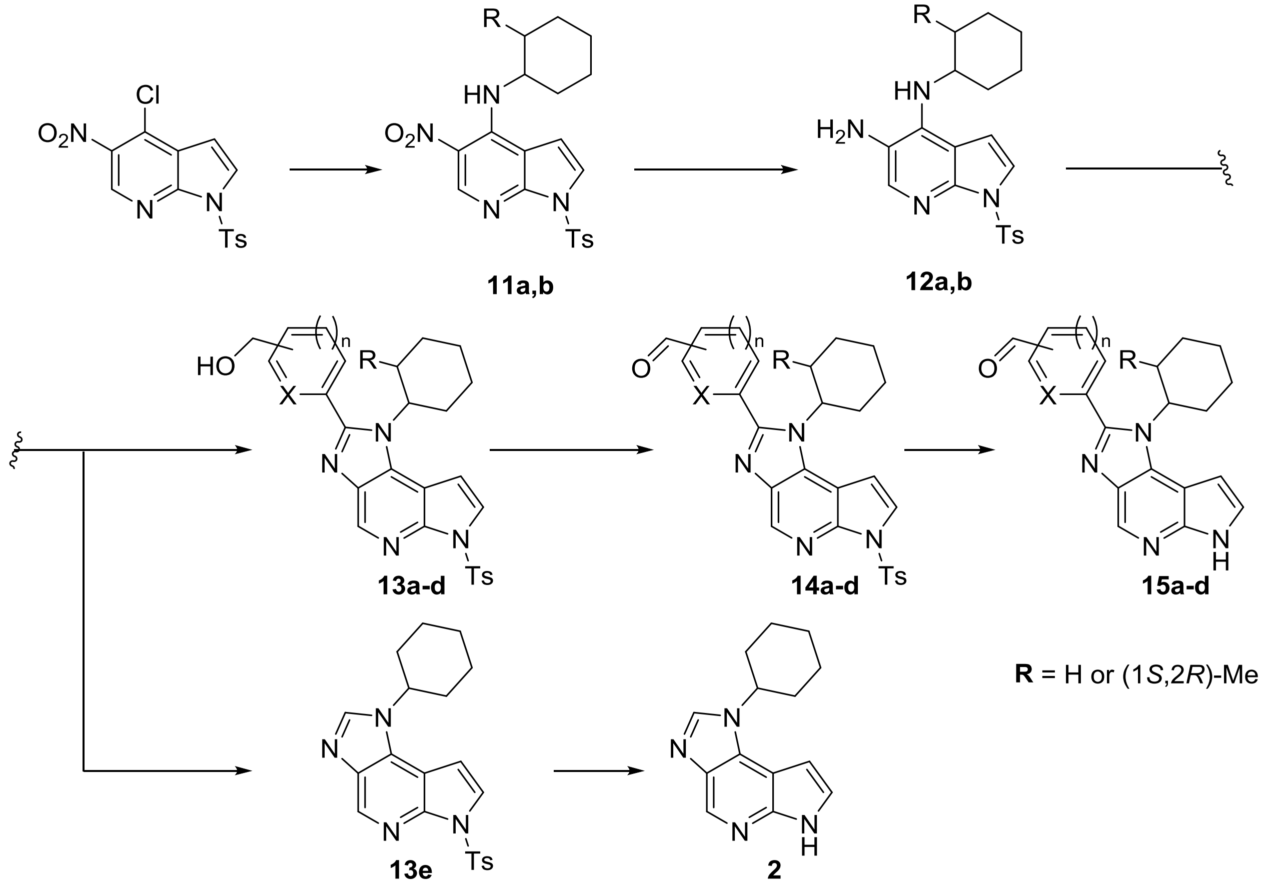

4-chloro-5-nitro-1-tosyl-1*H*-pyrrolo[2,3-*b*]pyridine and (1*S*,2*R*)-2-methylcyclohexan-1-amine hydrochloride were synthesized according to known procedures (Gehringer et al., 2014; Knupp and Frahm, 1984).

Nucleophilic aromatic substitution of cyclohexyl-side chains

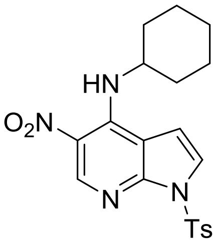

11a

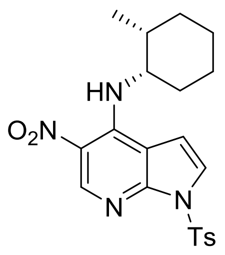

11b

*N-cyclohexyl-5-nitro-1-tosyl-1H-pyrrolo[2,3-b]pyridin-4-amine (**11a**).* To a stirred suspension of 4-chloro-5-nitro-1-tosyl-1*H*-pyrrolo[2,3-*b*]pyridine(Gehringer et al., 2014) (1407 mg, 4 mmol) in *i*PrOH (16 ml) was added cyclohexylamine (1190 mg, 12 mmol). The mixture was heated to reflux for 90 min and was then poured on sat. NH_4Cl / ice. The precipitate was filtered off, washed with water and dried in vacou, yielding 1537 mg (93 %) of **11a** as yellow solid which was carried on to the next step without further purification. ^1H NMR (200 MHz, $CDCl_3$) δ 9.20 – 8.95 (m, 2H), 8.05 (d, J = 8.4 Hz, 2H), 7.57 (d, J = 4.1 Hz, 1H), 7.29 (d, J =

8.4 Hz, 2H), 6.69 (d, J = 4.1 Hz, 1H), 3.94 (m, 1H), 2.38 (s, 3H), 2.19 – 1.99 (m, 2H), 1.93 – 1.58 (m, 3H), 1.57 – 1.31 (m, 5H) ^{13}C NMR (50 MHz, $CDCl_3$) δ 146.41, 145.9, 144.9, 134.9, 129.8, 128.6, 126.6, 123.6, 107.6, 106.7, 52.9, 33.5, 25.4, 24.4, 21.8 DC-MS (ESI) m/z: 415.4 $[M+H]^+$ HPLC t_{ret} = 9.439 min

*N-((1S,2R)-2-methylcyclohexyl)-5-nitro-1-tosyl-1H-pyrrolo[2,3-b]pyridin-4-amine (**11b**).* Following the same procedure as with **11a** but using (1*S*,2*R*)-2-methylcyclohexan-1-amine hydrochloride(Knupp and Frahm, 1984) (1.1 equiv) instead of cyclohexylamine and DIPEA as additional base (2.3 equiv). Same precipitation workup afforded **11b** as yellow solid. Yield: 83 % at 3 mmol scale. ^{1}H NMR (200 MHz, $CDCl_3$) δ 9.31 (br d, J = 8.3 Hz, 1H), 9.10 (s, 1H), 8.06 (d, J = 8.1 Hz, 2H), 7.57 (d, J = 4.1 Hz, 1H), 7.30 (d, J = 8.1 Hz, 2H), 6.72 (d, J = 4.1 Hz, 1H), 4.26 – 4.08 (m, 1H), 2.39 (s, 3H), 2.09 – 1.83 (m, 2H), 1.78 – 1.59 (m, 3H), 1.57 – 1.32 (m, 4H), 0.96 (d, J = 6.9 Hz, 3H). ^{13}C NMR (50 MHz, $CDCl_3$) δ 148.9, 146.5, 145.9, 145.6, 134.9, 129.8, 128.6, 126.8, 123.5, 107.7, 106.8, 54.9, 34.4, 30.2, 29.5, 23.7, 21.8, 21.6, 17.1 DC-MS (ESI) m/z: 451.2 $[M+Na]^+$ HPLC t_{ret} = 9.442 min

Reduction to vicinal aryl diamines

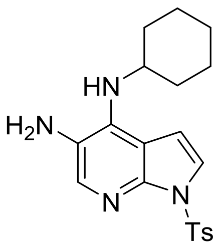

12a

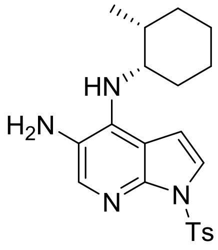

12b

*N^4-cyclohexyl-1-tosyl-1H-pyrrolo[2,3-b]pyridine-4,5-diamine (**12a**).* The crude **11a** (1500 mg, 3.6 mmol) was dissolved in MeOH / EtOAc (1:4, ca. 80 ml) and the solution was transferred to a glass-lined hydrogenation reactor. Raney Nickel (ca. 0,3 g as suspension in water, washed with MeOH) was added and hydrogen was bubbled through the magnetically stirred mixture. The reactor was purged with hydrogen, sealed and the pressure was adjusted to 0,5 MPa. The reactor was placed in a water bath at 45 °C and stirring was continued overnight. At this point TLC indicated full conversion. The catalyst was filtered off over celite and was washed several times with MeOH and EtOAc. The filtrate was evaporated to yield 1380 mg (99 %) of **12a** as greyish to purple foam. ^{1}H NMR (200 MHz, $CDCl_3$) δ 7.99 (d, J = 8.1 Hz, 2H), 7.82 (s, 1H), 7.44 (d, J = 4.1 Hz, 1H), 7.21 (d, J = 8.1 Hz, 2H), 6.53 (d, J = 4.1 Hz, 1H), 4.75 (br s, 1H), 3.77 – 3.57 (m, 1H), 2.96 (br s, 2H), 2.33 (s, 3H), 2.11 – 1.95 (m, 2H), 1.86 – 1.57 (m, 3H), 1.45 – 1.10 (m, 5H) ^{13}C NMR (50 MHz, $CDCl_3$) δ 145.8, 144.8,

141.0, 137.1, 135.7, 129.6, 128.0, 123.1, 122.3, 108.0, 105.0, 52.5, 34.4, 25.7, 2.9, 21.7 DC-MS (ESI) m/z: 385.4 $[M+H]^+$ HPLC t_{ret} = 5.895 min

N^4-((1S,2R)-2-methylcyclohexyl)-1-tosyl-1H-pyrrolo[2,3-b]pyridine-4,5-diamine ***(12b)*** was obtained from crude **11b** following the same procedure as with **12a**. Yield: 99 % at 3 mmol scale, obtained as purple foam. ^{1}H NMR (400 MHz, $CDCl_3$) δ 8.00 (d, J = 8.2 Hz, 2H), 7.83 (s, 1H), 7.43 (d, J = 4.1 Hz, 1H), 7.21 (d, J = 8.2 Hz, 2H), 6.55 (d, J = 4.2 Hz, 1H), 5.05 (bs, 1H), 3.99 – 3.86 (m, 1H), 2.92 (bs, 2H), 2.33 (s, 3H), 2.03 – 1.94 (m, 1H), 1.76 – 1.67 (m, 1H), 1.64 – 1.49 (m, 4H), 1.47 – 1.31 (m, 3H), 0.91 (d, J = 7.0 Hz, 3H). ^{13}C NMR (100 MHz, $CDCl_3$) δ 146.2, 144.7, 141.8, 137.9, 135.9, 129.5, 128.1, 123.0, 122.3, 108.2, 105.0, 54.0, 34.0, 30.1, 30.0, 23.0, 22.6, 21.7, 15.8 DC-MS (ESI) m/z: 399.2 $[M+H]^+$ HPLC t_{ret} = 5.986 min

These aryl diamines seem to be sensitive to air oxygen, since the product solution darkens rapidly upon air contact during filtration and the yields of subsequent reactions decrease if the diamine is stored over longer time periods at ambient temperature.

Imidazole ring closure reactions

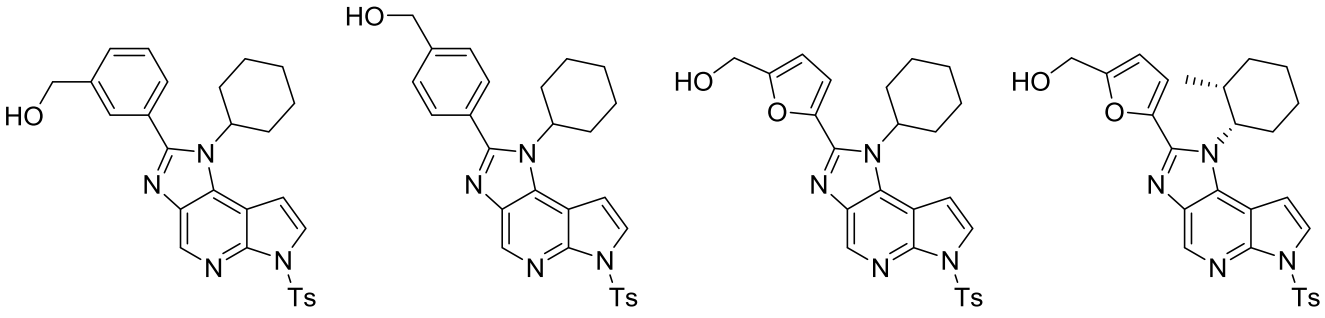

13a **13b** **13c** **13d**

Aryl substituted imidazole closure reactions were conducted according to a procedure reported by *(Beaulieu et al., 2003).*

(5-(1-cyclohexyl-6-tosyl-1,6-dihydroimidazo[4,5-d]pyrrolo[2,3-b]pyridin-2-yl)furan-2-yl)methanol (13c). A solution of 5-hydroxymethyl-2-furaldehyde (151 mg, 1.2 mmol) and **12a** (384 mg, 1.0 mmol) in DMF (10 ml) and water (350 µl) was stirred for about 10 min at ambient temperature before $KHSO_5$ (triple salt) (430 mg, 0.7 mmol) was added as solid in one portion. After stirring one hour at ambient temperature, TLC indicated total consumption of starting material. The mixture was poured on half-saturated $NaHCO_3$ solution, the precipitate was filtered off and was taken up in DCM. The organic phase was washed with brine once, dried over Na_2SO_4 and evaporated under reduced pressure. The residue was purified by flash

chromatography using gradient elution (petrol ether / (EtOAc+5% MeOH) 50 – 100%) to yield 253 mg (52 %) of **13c** as brownish foam. ^{1}H NMR (200 MHz, $CDCl_3$) δ 8.89 (s, 1H), 8.10 (d, *J* = 8.4 Hz, 2H), 7.83 (d, *J* = 4.1 Hz, 1H), 7.25 (d, *J* = 8.4 Hz, 2H), 6.92 (d, *J* = 4.1 Hz, 1H), 6.89 (d, *J* = 3.4 Hz, 1H), 6.44 (d, *J* = 3.4 Hz, 1H), 4.85 – 4.61 (m, 3H), 2.33 (s, 3H), 2.28 – 2.08 (m, 2H), 1.95 – 1.72 (m, 5H), 1.45 – 1.27 (m, 3H) ^{13}C NMR (50 MHz, $CDCl_3$) δ 156.9, 145.3, 144.4, 143.6, 142.9, 138.3, 136.9, 135.4, 133.2, 129.7, 128.4, 124.8, 114.5, 109.8, 107.8, 104.7, 57.4, 57.3, 30.8, 25.8, 24.9, 21.7 DC-MS (ESI) m/z: 491.3 $[M+H]^+$ HPLC t_{ret} = 8.112 min

*(3-(1-cyclohexyl-6-tosyl-1,6-dihydroimidazo[4,5-d]pyrrolo[2,3-b]pyridin-2-yl)phenyl)methanol (**13a**)* was obtained from 264 mg **12a** and 112 mg **9a** following the same procedure as described for **13c** with a reaction time of one hour at ambient temperature. Crude product afforded flash purification using gradient elution (petrol ether / (EtOAc+5% MeOH) 30 – 100%) to yield 247 mg (72 %) of **13a** as brownish foam. ^{1}H NMR (200 MHz, $CDCl_3$) δ 8.93 (s, 1H), 8.11 (d, *J* = 8.3 Hz, 2H), 7.85 (d, *J* = 4.1 Hz, 1H), 7.62 (s, 1H), 7.53 – 7.39 (m, 3H), 7.26 (d, *J* = 8.3 Hz, 3H), 6.97 (d, *J* = 4.1 Hz, 1H), 4.75 (s, 2H), 4.56 – 4.30 (m, 1H), 2.95 (bs, 1H), 2.40 – 2.12 (m, 5H), 2.04 – 1.72 (m, 5H), 1.46 – 1.29 (m, 3H). ^{13}C NMR (50 MHz, $CDCl_3$) δ 153.9, 145.3, 142.9, 142.5, 138.2, 136.6, 135.4, 132.9, 130.3, 129.7, 128.9, 128.7, 128.4, 128.3, 128.1, 124.9, 107.8, 105.0, 64.5, 56.9, 31.1, 25.6, 24.9, 21.8 FAB-MS m/z: 501.2 $[M+H]^+$ HPLC t_{ret} = 8.333 min

*(4-(1-cyclohexyl-6-tosyl-1,6-dihydroimidazo[4,5-d]pyrrolo[2,3-b]pyridin-2-yl)phenyl)methanol (**13b**)* was obtained from 384 mg **12a** and 164 mg **9b** following the above described procedure for **13c** with a reaction time of two hours at ambient temperature. Crude product afforded flash purification using gradient elution (petrol ether / (EtOAc+5% MeOH) 50 – 100%) to yield 343 mg (69 %) of **13b** as brownish foam. ^{1}H NMR (200 MHz, $CDCl_3$) δ 8.94 (s, 1H), 8.10 (d, *J* = 8.4 Hz, 2H), 7.83 (d, *J* = 4.1 Hz, 1H), 7.44 (q, *J* = 8.5 Hz, 4H), 7.25 (d, *J* = 8.4 Hz, 2H), 6.97 (d, *J* = 4.1 Hz, 1H), 4.77 (s, 2H), 4.40 (tt, *J* = 12.1, 3.8 Hz, 1H), 3.76 (br s, 1H), 2.40 – 2.14 (m, 5H), 2.00 – 1.71 (m, 5H), 1.47 – 1.28 (m, 3H). ^{13}C NMR (50 MHz, $CDCl_3$) δ 154.0, 145.3, 144.0, 142.9, 138.3, 137.0, 135.4, 133.0, 129.7, 129.5, 129.1, 128.4, 126.9, 124.7, 107.8, 105.1, 64.2, 56.8, 31.1, 25.6, 24.9, 21.7 DC-MS (ESI) m/z: 522.9 $[M+Na]^+$ HPLC t_{ret} = 8.077 min

*(5-(1-((1S,2R)-2-methylcyclohexyl)-6-tosyl-1,6-dihydroimidazo[4,5-d]pyrrolo[2,3-b]pyridin-2-yl)furan-2-yl)methanol (**13d**)* was obtained from 400 mg **12b** and 152 mg 5-hydroxymethyl-2-furaldehyde following the above described procedure for **13c**, but reaction was run

overnight at ambient temperature. Crude product afforded flash purification using gradient elution (hexane / (EtOAc+10% *i*PrOH) 50 – 100%) to yield 162 mg (32 %) of **13d** as brownish foam. ^{1}H NMR (400 MHz, $CDCl_3$) δ 8.88 (s, 1H), 8.10 (d, *J* = 8.1 Hz, 2H), 7.79 (d, *J* = 3.8 Hz, 1H), 7.25 (d, *J* = 8.1 Hz, 2H), 6.87 – 6.75 (m, 2H), 6.40 (d, *J* = 2.9 Hz, 1H), 4.81 – 4.72 (m, 1H), 4.68 (s, 2H), 2.53 – 2.42 (m, 1H), 2.33 (s, 3H), 2.14 (s, 1H), 1.86 (d, *J* = 12.3 Hz, 1H), 1.70 (t, *J* = 18.6 Hz, 3H), 1.58 – 1.27 (m, 4H), 0.87 (d, *J* = 7.0 Hz, 3H). ^{13}C NMR (100 MHz, $CDCl_3$) δ 156.5, 145.3, 144.7, 143.7, 143.2, 138.4, 136.2, 135.5, 134.5, 129.7, 128.4, 124.8, 115.1, 109.3, 107.8, 103.6, 62.5, 57.4, 34.3, 32.0, 27.0, 25.4, 21.7, 19.5, 13.0 DC-MS (ESI) m/z: 505.3 $[M+H]^+$ HPLC t_{ret} = 7.955 min

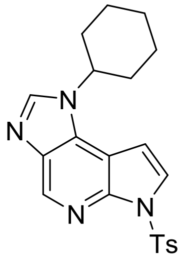

13e

1-cyclohexyl-6-tosyl-1,6-dihydroimidazo[4,5-d]pyrrolo[2,3-b]pyridine (13e). **12a** (38 mg, 0.1 mmol), triethyl orthoformate (45 mg, 0.3 mmol) and TsOH x H_2O (2 mg, 0.01 mmol) were dissolved in 1 ml toluene and heated to 70 °C heating block temperature for two hours. TLC indicated full consumption of starting material and the mixture was poured on sat. $NaHCO_{3(aq)}$. The biphasic mixture was extracted with EtOAc (5x10 ml) and the combined extracts were dried over Na_2SO_4 and evaporated to dryness. The residue was subjected to flash purification using gradient elution (petrol ether / (EtOAc+5% MeOH) 50 – 100%) to yield 29 mg (75 %) of **13e** as brownish solid. ^{1}H NMR (400 MHz, $CDCl_3$) δ 8.89 (s, 1H), 8.07 (d, *J* = 8.2 Hz, 2H), 8.02 (s, 1H), 7.77 (d, *J* = 3.9 Hz, 1H), 7.22 (d, *J* = 8.2 Hz, 2H), 6.74 (d, *J* = 3.9 Hz, 1H), 4.44 – 4.30 (m, 1H), 2.31 (s, 3H), 2.29 – 2.18 (m, 2H), 2.06 – 1.93 (m, 2H), 1.87 – 1.67 (m, 3H), 1.61 – 1.45 (m, 2H), 1.39 – 1.22 (m, 1H). ^{13}C NMR (100 MHz, $CDCl_3$) δ 145.2, 142.9, 139.9, 138.5, 137.2, 135.5, 132.5, 129.6, 128.3, 125.2, 107.5, 101.7, 57.1, 33.5, 25.7, 25.3, 21.7 DC-MS (ESI) m/z: 417.2 $[M+Na]^+$ HPLC t_{ret} = 7.427 min

Preparation of tosylated aryl aldehydes (14a-d)

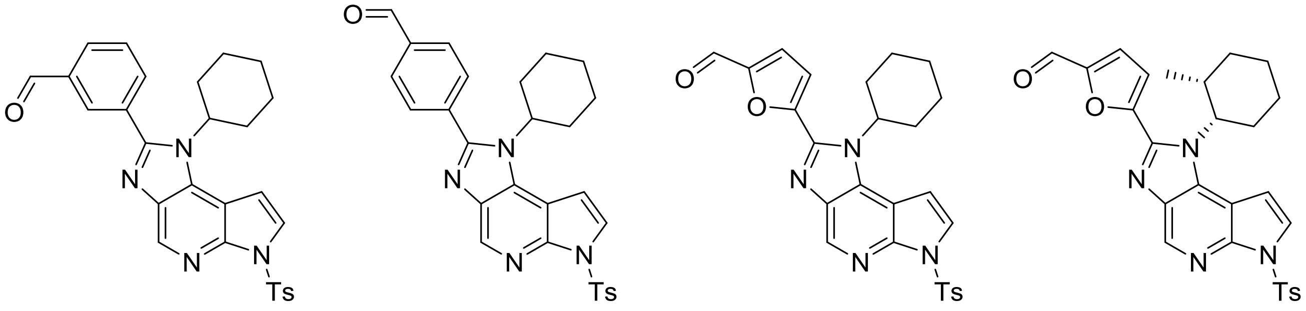

14a **14b** **14c** **14d**

Oxidation of benzylic alcohols **13a-d** was performed using Dess-Martin-Periodinane, which was prepared from 2-iodo-benzoic acid following the procedures of (Frigerio et al., 1999) and (Ireland and Liu, 1993).

*5-(1-cyclohexyl-6-tosyl-1,6-dihydroimidazo[4,5-d]pyrrolo[2,3-b]pyridin-2-yl)furan-2-carbaldehyde (**14c**).* To an ice-cooled solution of **13c** (250 mg, 0.51 mmol) in dry DCM (11 ml) was added Dess-Martin-Periodinane (259 mg, 0.61 mmol) as solid in one portion. The stirred reaction mixture was allowed to reach ambient temperature slowly and after one hour TLC indicated full conversion. The reaction was quenched with sat. $NaHCO_3$ and the biphasic mixture was extracted with DCM (5 x 20 ml). The combined organic extracts were dried over Na_2SO_4, evaporated and the residue purified by flash chromatography (gradient elution DCM / MeOH 1-5%) to yield 200 mg (80 %) of **14c** as brownish foam. ^{1}H NMR (200 MHz, $CDCl_3$) δ 9.77 (s, 1H), 8.93 (s, 1H), 8.12 (d, J = 8.5 Hz, 2H), 7.86 (d, J = 4.1 Hz, 1H), 7.41 (d, J = 3.7 Hz, 1H), 7.32 – 7.22 (m, 3H), 6.98 (d, J = 4.1 Hz, 1H), 5.10 – 4.88 (m, 1H), 2.43 – 2.20 (m, 5H), 2.11 – 1.84 (m, 5H), 1.63 – 1.43 (m, 3H) ^{13}C NMR (50 MHz, $CDCl_3$) δ 177.4, 153.2, 149.7, 145.4, 143.2, 142.6, 139.0, 137.4, 135.4, 133.8, 129.8, 128.4, 125.0, 122.0, 115.4, 107.8, 104.6, 57.9, 31.0, 26.0, 25.0, 21.8 DC-MS (ESI) m/z: 511.3 $[M+Na]^+$ HPLC t_{ret} = 8.520 min

*3-(1-cyclohexyl-6-tosyl-1,6-dihydroimidazo[4,5-d]pyrrolo[2,3-b]pyridin-2-yl)benzaldehyde (**14a**)* was obtained from 240 mg **13a** following the above described procedure for **14c** with a reaction time of one hour. Crude product afforded flash purification using gradient elution (petrol ether / (EtOAc+5% MeOH) 30 – 80%) to yield 220 mg (92 %) of **14a** as brownish foam. ^{1}H NMR (200 MHz, $CDCl_3$) δ 8.93 (s, 1H), 8.11 (d, J = 8.3 Hz, 2H), 7.85 (d, J = 4.1 Hz, 1H), 7.62 (s, 1H), 7.53 – 7.39 (m, 3H), 7.26 (d, J = 8.3 Hz, 3H), 6.97 (d, J = 4.1 Hz, 1H), 4.75 (s, 2H), 4.56 – 4.30 (m, 1H), 2.95 (bs, 1H), 2.40 – 2.12 (m, 5H), 2.04 – 1.72 (m, 5H), 1.46 – 1.29 (m, 3H). ^{13}C NMR (50 MHz, $CDCl_3$) δ 153.9, 145.3, 142.9, 142.5, 138.2, 136.6,

135.4, 132.9, 130.3, 129.7, 128.9, 128.7, 128.4, 128.3, 128.1, 124.9, 107.8, 105.0, 64.5, 56.9, 31.1, 25.6, 24.9, 21.8 FAB-MS m/z: 499.2 $[M+H]^+$ HPLC t_{ret} = 8.751 min

4-(1-cyclohexyl-6-tosyl-1,6-dihydroimidazo[4,5-d]pyrrolo[2,3-b]pyridin-2-yl)benzaldehyde ***(14b)*** was obtained from 148 mg **13b** following the above described procedure for **14c** with a reaction time of three hours. Crude product afforded flash purification using gradient elution (petrol ether / (EtOAc+5% MeOH) 30 – 80%) to yield 118 mg (80 %) of **14b** as brownish foam. ^{1}H NMR (200 MHz, $CDCl_3$) δ 10.10 (s, 1H), 8.94 (s, 1H), 8.17 – 7.95 (m, 4H), 7.84 (d, *J* = 4.0 Hz, 1H), 7.79 (d, *J* = 7.8 Hz, 2H), 7.24 (d, *J* = 7.8 Hz, 2H), 6.97 (d, *J* = 4.0 Hz, 1H), 4.53 – 4.28 (m, 1H), 2.41 – 2.17 (m, 5H), 2.05 – 1.75 (m, 5H), 1.55 – 1.28 (m, 3H). ^{13}C NMR (50 MHz, $CDCl_3$) δ 191.6, 152.5, 145.3, 142.9, 138.8, 137.3, 137.2 136.4, 135.3, 133.2, 130.3, 130.0, 129.7, 128.3, 124.9, 107.8, 104.9, 57.1, 31.1, 25.6, 24.8, 21.7 DC-MS (ESI) m/z: 520.9 $[M+H]^+$ HPLC t_{ret} = 8.347 min

5-(1-((1S,2R)-2-methylcyclohexyl)-6-tosyl-1,6-dihydroimidazo[4,5-d]pyrrolo[2,3-b]pyridin-2-yl)furan-2-carbaldehyde ***(14d)*** was obtained from 56 mg **13d** following the above described procedure for **14c** with a reaction time of 30 minutes. Crude product afforded flash purification using gradient elution (petrol ether / (hexane / (EtOAc+10% *i*PrOH) 50 – 100%) to yield 49 mg (88 %) of **14d** as off-white foam. ^{1}H NMR (400 MHz, $CDCl_3$) δ 9.75 (s, 1H), 8.93 (s, 1H), 8.11 (d, *J* = 8.3 Hz, 2H), 7.84 (d, *J* = 4.0 Hz, 1H), 7.39 (d, *J* = 3.6 Hz, 1H), 7.27 (d, *J* = 8.3 Hz, 2H), 7.20 (d, *J* = 3.6 Hz, 1H), 6.88 (d, *J* = 4.0 Hz, 1H), 4.96 (dt, *J* = 13.0, 3.7 Hz, 1H), 2.73 – 2.61 (m, 1H), 2.35 (s, 3H), 2.30 – 2.15 (m, 1H), 1.98 – 1.85 (m, 2H), 1.85 – 1.73 (m, 2H), 1.63 – 1.50 (m, 2H), 1.46 – 1.35 (m, 1H), 1.04 (d, *J* = 7.2 Hz, 3H). ^{13}C NMR (100 MHz, $CDCl_3$) δ 177.5, 153.0, 149.8, 145.4, 143.4, 142.8, 139.0, 136.7, 135.5, 135.0, 129.7, 128.4, 125.0, 121.7, 116.1, 107.9, 103.4, 63.0, 34.5, 32.1, 27.1, 25.6, 21.7, 19.5, 13.0 DC-MS (ESI) m/z: 525.3 $[M+Na]^+$ HPLC t_{ret} = 8.188 min

Preparation of detosylated compounds 2 and 15a-d

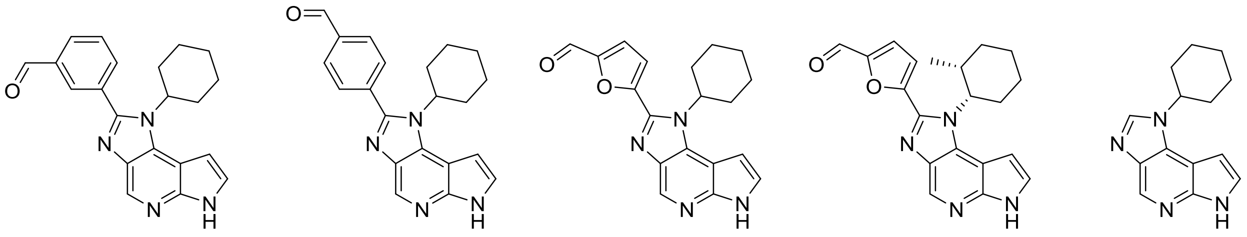

15a 15b 15c 15d 2

*5-(1-cyclohexyl-1,6-dihydroimidazo[4,5-d]pyrrolo[2,3-b]pyridin-2-yl)furan-2-carbaldehyde (**15c**)*. To a stirred solution of **14c** (200 mg, 0.41 mmol) in dry THF (9 ml) and dry MeOH (3 ml) was added cesium carbonate (400 mg, 1.23 mmol) at ambient temperature. After four hours at ambient temperature, the reaction was complete according to TLC. The reaction was quenched with sat. NH_4Cl, followed by extraction with EtOAc (5 x 15 ml). The combined organic phases were dried over Na_2SO_4, evaporated and the residue purified by flash chromatography (gradient elution DCM / MeOH 4-8%) to yield 69 mg (50 %) of **15c** as yellow solid. ^{1}H NMR (200 MHz, $CDCl_3$) δ 12.05 (br s, 1H), 9.78 (s, 1H), 8.89 (s, 1H), 7.51 (d, J = 3.4 Hz, 1H), 7.42 (d, J = 3.7 Hz, 1H), 7.30 (d, J = 3.7 Hz, 1H), 6.90 (d, J = 3.4 Hz, 1H), 5.17 – 4.94 (m, 1H), 2.63 – 2.36 (m, 2H), 2.18 – 1.79 (m, 5H), 1.69 – 1.43 (m, 3H). ^{13}C NMR (50 MHz, $CDCl_3$) δ 177.5, 153.1, 150.3, 145.1, 141.4, 136.8, 135.7, 134.4, 123.6, 122.0, 114.7, 105.2, 100.9, 57.8, 30.9, 26.0, 25.0 DC-MS (ESI) m/z: 357.2 $[M+Na]^+$ HPLC t_{ret} = 6.991 min

*3-(1-cyclohexyl-1,6-dihydroimidazo[4,5-d]pyrrolo[2,3-b]pyridin-2-yl)benzaldehyde (**15a**)* was obtained from 220 mg **14a** following the above described procedure for **15c** with a reaction time of three hours at ambient temperature. Crude product afforded flash purification using gradient elution (DCM / MeOH 3 – 10%) to yield 120 mg (79 %) of **15a** as beige solid. ^{1}H NMR (200 MHz, $CDCl_3$) δ 11.85 (br s, 1H), 10.13 (s, 1H), 8.91 (s, 1H), 8.21 (s, 1H), 8.07 (d, J = 7.6 Hz, 1H), 7.96 (d, J = 7.6 Hz, 1H), 7.74 (t, J = 7.6 Hz, 1H), 7.52 (d, J = 3.5 Hz, 1H), 6.90 (d, J = 3.5 Hz, 1H), 4.57 – 4.34 (m, 1H), 2.53 (dd, J = 23.9, 12.3 Hz, 2H), 2.12 – 1.76 (m, 5H), 1.59 – 1.31 (m, 3H). ^{13}C NMR (50 MHz, $CDCl_3$) δ 191.6, 151.4, 144.6, 136.9, 136.2, 135.5, 135.3, 134.0, 132.4, 131.1, 130.7, 129.7, 123.5, 105.5, 101.1, 57.2, 31.1, 25.8, 24.9 FAB-MS m/z: 345.2 $[M+H]^+$ HPLC t_{ret} = 6.152 min

*4-(1-cyclohexyl-1,6-dihydroimidazo[4,5-d]pyrrolo[2,3-b]pyridin-2-yl)benzaldehyde (**15b**)* was obtained from 258 mg **14b** following the above described procedure for **15c** with a reaction time of three hours at ambient temperature. Crude product afforded flash purification

using gradient elution (DCM / MeOH 3 – 10%) to yield 114 mg (64 %) of **15b** as off-white solid. ^{1}H NMR (200 MHz, DMSO) δ 12.03 (br s, 1H), 10.15 (s, 1H), 8.68 (s, 1H), 8.12 (d, *J* = 7.8 Hz, 2H), 7.93 (d, *J* = 7.8 Hz, 2H), 7.61 – 7.50 (m, 1H), 6.84 – 6.76 (m, 1H), 4.55 – 4.24 (m, 1H), 2.46 – 2.23 (m, 2H), 1.88 (dd, *J* = 35.2, 24.7 Hz, 5H), 1.52 – 1.22 (m, 3H). ^{13}C NMR (50 MHz, DMSO) δ 192.5, 150.1, 144.2, 136.2, 136.2, 135.8, 134.7, 132.3, 130.1, 129.4, 123.8, 104.1, 100.0, 56.1, 30.3, 25.1, 24.3 DC-MS (ESI) m/z: 342.9 [M-H]$^-$ HPLC t_{ret} = 6.975 min

5-(1-((1S,2R)-2-methylcyclohexyl)-1,6-dihydroimidazo[4,5-d]pyrrolo[2,3-b]pyridin-2-yl)furan-2-carbaldehyde (15d) To a solution of **14d** (128 mg, 0.26 mmol) in 2.5 ml THF and 2.5 ml MeOH was added 2.5 ml 0.5 M $NaOH_{(aq)}$. The solution was heated to 50 °C water-bath temperature and stirred for 5 hours. TLC indicated complete conversion and the reaction was quenched with sat. NH_4Cl followed by extractive workup with EtOAc (5x10 ml). The combined organic phases were dried over Na_2SO_4 and evaporated. The residue was subjected to flash purification with gradient elution (DCM / MeOH 4 – 10%) yielding 56 mg (63 %) of **15d** as yellow solid. ^{1}H NMR (400 MHz, $CDCl_3$) δ 12.42 (br s, 1H), 9.76 (s, 1H), 8.90 (s, 1H), 7.50 (d, *J* = 3.0 Hz, 1H), 7.39 (d, *J* = 3.6 Hz, 1H), 7.20 (d, *J* = 3.6 Hz, 1H), 6.80 (d, *J* = 3.0 Hz, 1H), 5.07 (dt, *J* = 13.0, 3.6 Hz, 1H), 2.84 – 2.71 (m, 1H), 2.44 – 2.25 (m, 1H), 1.99 – 1.88 (m, 2H), 1.87 – 1.76 (m, 2H), 1.65 – 1.53 (m, 2H), 1.50 – 1.37 (m, 1H), 1.09 (d, *J* = 7.2 Hz, 3H). ^{13}C NMR (100 MHz, $CDCl_3$) δ 177.6, 152.9, 150.5, 145.4, 141.5, 136.8, 135.7, 135.0, 123.6, 121.7, 115.6, 105.3, 99.6, 62.8, 34.4, 32.2, 27.2, 25.6, 19.6, 13.0 DC-MS (ESI) m/z: 371.3 [M+Na]$^+$ HPLC t_{ret} = 6.828 min

1-cyclohexyl-1,6-dihydroimidazo[4,5-d]pyrrolo[2,3-b]pyridine (2) was obtained from 54 mg **13e** following the above described procedure for **15c** with a reaction time of three hours at ambient temperature. Crude product afforded flash purification using gradient elution (DCM / MeOH 3 – 10%) to yield 23 mg (70 %) of **2** as off-white solid. ^{1}H NMR (200 MHz, $CDCl_3$) δ 11.93 (br s, 1H), 8.86 (s, 1H), 8.02 (s, 1H), 7.47 (d, *J* = 3.4 Hz, 1H), 6.69 (d, *J* = 3.4 Hz, 1H), 4.52 (tt, *J* = 11.4, 3.7 Hz, 1H), 2.47 – 2.28 (m, 2H), 2.11 – 1.29 (m, 8H). ^{13}C NMR (50 MHz, $CDCl_3$) δ 144.6, 138.9, 136.1, 135.4, 133.2, 123.7, 105.0, 97.4, 57.0, 33.5, 25.8, 25.5 ESI-HRMS [M+H]$^+$ calculated for $C_{14}H_{16}N_4$: 241.1448, found 241.1450 HPLC t_{ret} = 4.566 min

Preparation of Michael acceptor bearing compounds

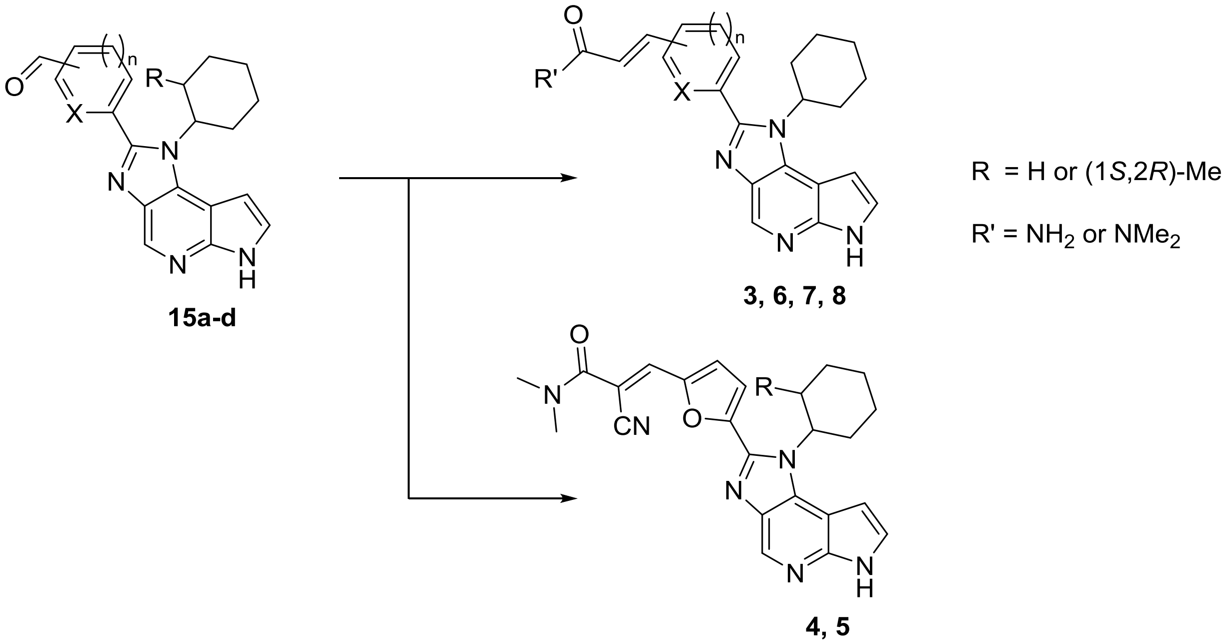

Acrylamides 3 and 6-8 via Horner-Wadsworth-Emmons-reaction

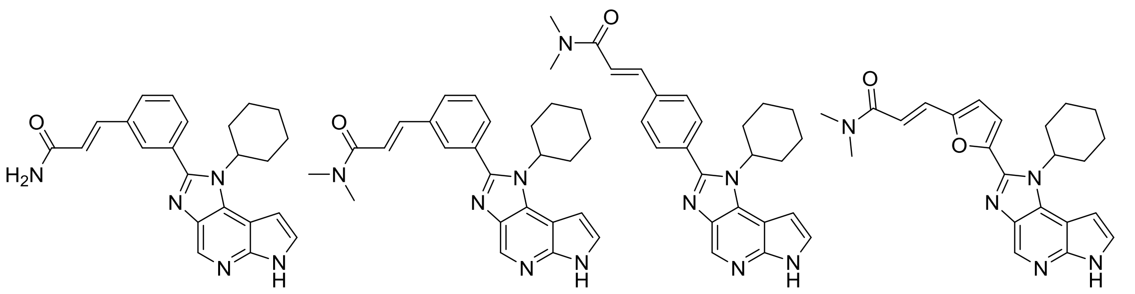

6 7 8 3

*(E)-3-(5-(1-cyclohexyl-1,6-dihydroimidazo[4,5-d]pyrrolo[2,3-b]pyridin-2-yl)furan-2-yl)-N,N-dimethylacrylamide (**3**).* In an oven-dried schlenk tube LiCl (8 mg, 180 µmol) was suspended in dry MeCN (2 ml) under argon atmosphere. Subsequently were added diethyl (2-(dimethylamino)-2-oxoethyl)phosphonate (**10b**) (41 mg, 180 µmol) and DBU (27 µl, 180 µmol). After stirring for 10 min at ambient temperature **15c** (40 mg, 120 µmol) was added as suspension in dry chloroform (500 µl). The reaction was complete after another 90 min at ambient temperature. Sat. NH_4Cl was added, followed by extractive workup with DCM (5 x 10 ml). The combined organic extracts were dried over Na_2SO_4, evaporated to dryness and the residue purified by flash chromatography with gradient elution (DCM / MeOH 4-10%) to yield 42 mg (88 %) of **3** as slightly yellowish solid. ^{1}H NMR (200 MHz, $CDCl_3$) δ 11.92 (br s, 1H), 8.88 (s, 1H), 7.55 (d, J = 15.2 Hz, 1H), 7.49 (d, J = 3.5 Hz, 1H), 7.10 (d, J = 3.6 Hz, 1H), 6.94 (d, J = 15.2 Hz, 1H), 6.86 (d, J = 3.5 Hz, 1H), 6.75 (d, J = 3.6 Hz, 1H), 5.09 – 4.84 (m, 1H), 3.12 (d, J = 17.1 Hz, 6H), 2.67 – 2.35 (m, 2H), 2.18 – 1.78 (m,

5H), 1.64 – 1.37 (m, 3H). ^{13}C NMR (50 MHz, $CDCl_3$) δ 166.8, 153.5, 146.6, 145.4, 143.1, 136.9, 136.0, 134.4, 129.1, 123.7, 116.7, 115.7, 115.4, 105.4, 101.0, 57.2, 37.2, 35.9, 30.6, 25.7, 24.7 ESI-HRMS [M+H]$^+$ calculated for $C_{23}H_{25}N_5O_2$: 404.2081, found: 404.2078 HPLC t_{ret} = 7.148 min (purity 98.4 %)

(E)-3-(3-(1-cyclohexyl-1,6-dihydroimidazo[4,5-d]pyrrolo[2,3-b]pyridin-2-yl)phenyl)acrylamide (6) was obtained from 55 mg **15a** and 22 mg **10a** following the above described procedure for **3** with a reaction time of two hours at ambient temperature. Crude product afforded flash purification using gradient elution (DCM / MeOH 8 – 16%) to yield 22 mg (78 %) of **6** as off-white solid. ^{1}H NMR (200 MHz, DMSO) δ 11.97 (bs, 1H), 8.65 (s, 1H), 7.87 (s, 1H), 7.82 – 7.45 (m, 6H), 7.18 (s, 1H), 6.86 – 6.68 (m, 2H), 4.52 – 4.26 (m, 1H), 2.44 – 2.22 (m, 2H), 2.07 – 1.64 (m, 5H), 1.53 – 1.25 (m, 3H). ^{13}C NMR (50 MHz, DMSO) δ 166.5, 151.1, 144.5, 138.4, 135.8, 135.4, 134.8, 132.4, 131.6, 130.2, 129.4, 128.7, 128.5, 124.0, 123.5, 104.3, 100.0, 56.1, 30.3, 25.2, 24.3. ESI-HRMS [M+H]$^+$ calculated for $C_{23}H_{23}N_5O$: 386.1975, found: 386.1973 HPLC t_{ret} = 6.341 min (purity 100 %)

(E)-3-(3-(1-cyclohexyl-1,6-dihydroimidazo[4,5-d]pyrrolo[2,3-b]pyridin-2-yl)phenyl)-N,N-dimethylacrylamide (7) was obtained from 26 mg **15a** and 25 mg **10b** following the above described procedure for **3** with a reaction time of two hours at ambient temperature. Crude product afforded flash purification using gradient elution (DCM / MeOH 3 – 10%) to yield 23 mg (74 %) of **7** as off-white solid. ^{1}H NMR (200 MHz, $CDCl_3$) δ 11.70 (br s, 1H), 8.89 (s, 1H), 7.89 (s, 1H), 7.81 – 7.43 (m, 5H), 7.00 (d, J = 15.5 Hz, 1H), 6.88 (d, J = 3.3 Hz, 1H), 4.61 – 4.36 (m, 1H), 3.12 (d, J = 19.3 Hz, 6H), 2.62 – 2.35 (m, 2H), 2.08 – 1.71 (m, 5H), 1.62 – 1.32 (m, 3H). ^{13}C NMR (50 MHz, $CDCl_3$) δ 166.5, 152.4, 144.5, 141.4, 136.3, 136.0, 135.4, 133.9, 131.8, 130.3, 129.6, 129.3, 128.6, 123.4, 119.0, 105.5, 101.1, 56.9, 37.6, 36.1, 31.1, 25.8, 24.9 ESI-HRMS [M+H]$^+$ calculated for $C_{25}H_{27}N_5O$: 414.2288, found: 414.2285 HPLC t_{ret} = 6.636 min (purity 98.9 %)

(E)-3-(4-(1-cyclohexyl-1,6-dihydroimidazo[4,5-d]pyrrolo[2,3-b]pyridin-2-yl)phenyl)-N,N-dimethylacrylamide (8) was obtained from 50 mg **15b** and 49 mg **10b** following the above described procedure for **3** with a reaction time of four hours at ambient temperature. Crude product afforded flash purification using gradient elution (DCM / MeOH 4 – 10%) to yield 42 mg (70 %) of **8** as white solid. ^{1}H NMR (200 MHz, $CDCl_3$ + MeOD) δ 8.77 (s, 1H), 7.80 – 7.59 (m, 5H), 7.41 (d, J = 3.6 Hz, 1H), 6.99 (d, J = 15.5 Hz, 1H), 6.84 (d, J = 3.6 Hz, 1H), 4.47 (tt, J = 12.3, 4.3 Hz, 1H), 3.13 (d, J = 24.8 Hz, 6H), 2.63 – 2.40 (m, 2H), 2.06 – 1.74 (m, 5H), 1.55 – 1.30 (m, 3H). indole NH was exchanged by MeOD but residual peak was visible

with 0.2 protons. ^{13}C NMR (50 MHz, $CDCl_3$ + MeOD) δ 167.2, 152.7, 144.9, 141.9, 137.2, 136.6, 135.7, 134.1, 132.3, 130.4, 128.5, 123.6, 119.2, 105.5, 101.2, 56.7, 37.4, 35.9, 30.7, 25.4, 24.5 ESI-HRMS $[M+H]^+$ calculated for $C_{25}H_{27}N_5O$: 414.2288, found: 414.2285 HPLC t_{ret} = 6.938 min (purity 100 %)

Cyano acrylamides 4 and 5 via Knoevenagel reaction

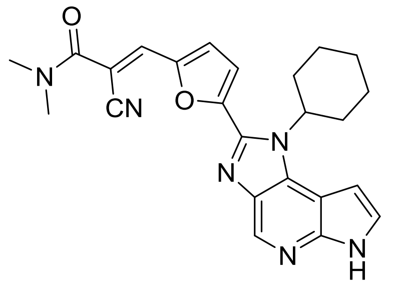

4

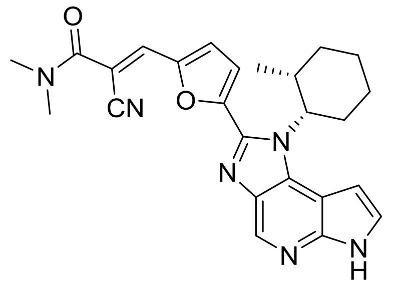

5

(E/Z)-2-cyano-3-(5-(1-cyclohexyl-1,6-dihydroimidazo[4,5-d]pyrrolo[2,3-b]pyridin-2-yl)furan-2-yl)-N,N-dimethylacrylamide (4). *N,N*-Dimethyl-2-cyanoacetamide (18 mg, 162 µmol) and **15c** (27 mg, 81 µmol) were suspended in EtOH (4 ml) in a screw-cap reaction tube. Piperidine (17 µl, 162 µmol) and acetic acid (10 µl, 162 µmol) were added subsequently. The reaction tube was sealed and the reaction was placed in a heating block at 80 °C. After one hour the reaction was cooled to ambient temperature, diluted with EtOAc and poured on brine. The phases were separated and the aqueous phase was extracted with EtOAc (4 x 10 ml). The combined organic phases were dried over Na_2SO_4, evaporated to dryness and the residue purified by flash chromatography (gradient elution DCM / MeOH 4-16%) to yield 32 mg (93 %) of **4** as yellow solid. ^1H NMR (400 MHz, DMSO) δ 12.05 (br s, 1H), 8.72 – 8.63 (m, 1H), 7.78 (s, 1H), 7.56 (s, 1H), 7.49 (d, J = 3.5 Hz, 1H), 7.39 (d, J = 3.5 Hz, 1H), 6.86 – 6.78 (m, 1H), 4.99 – 4.86 (m, 1H), 3.21 – 2.87 (m, 6H), 2.42 – 2.27 (m, 2H), 2.02 (d, J = 10.7 Hz, 2H), 1.95 – 1.84 (m, 2H), 1.80 – 1.70 (m, 1H), 1.66 – 1.42 (m, 3H). ^{13}C NMR (100 MHz, DMSO) δ 162.8, 149.5, 148.4, 144.6, 140.5, 136.3, 135.4, 135.1, 132.8, 124.1, 121.9, 116.3, 115.9, 104.1, 101.9, 100.3, 56.1, 30.4, 30.0, 24.9, 24.3 ESI-HRMS $[M+H]^+$ calculated for $C_{24}H_{24}N_6O_2$: 429.2034, found: 429.2045 HPLC t_{ret} = 6.172 min and 6.985 min (E/Z mixture, purity 100 %)

(E/Z)-2-cyano-N,N-dimethyl-3-(5-(1-((1S,2R)-2-methylcyclohexyl)-1,6-dihydroimidazo[4,5-d]pyrrolo[2,3-b]pyridin-2-yl)furan-2-yl)acrylamide (5). *N,N*-Dimethyl-2-cyanoacetamide (12 mg, 108 µmol) and **15d** (27 mg, 72 µmol) were suspended in *i*PrOH (2 ml) in a screw-cap reaction tube. Piperidine was added as 1 M solution in *i*PrOH (14 µl, 14 µmol). The reaction

tube was sealed and the reaction was placed in a heating block at 60 °C. After six hours the reaction was cooled to ambient temperature, diluted with EtOAc and poured on sat. NH_4Cl. The phases were separated and the aqueous phase was extracted with EtOAc (5x10 ml). The combined organic phases were dried over Na_2SO_4, evaporated to dryness and the residue purified by flash chromatography with gradient elution (DCM / MeOH 4 - 12%) to yield 17 mg (53 %) of **5** as yellow solid. ^{1}H NMR (400 MHz, DMSO) δ 12.04 (br s, 1H), 8.66 (d, J = 5.0 Hz, 1H), 7.76 (s) and 7.63 (s, 1H), 7.56 – 7.50 (m, 1H), 7.48 – 7.42 and 7.29 – 7.22 (m, 1H), 7.14 (s, 1H), 6.80 (s, 1H), 5.09 – 4.87 (m, 1H), 3.18 – 2.82 (m, 6H), 2.49 – 2.37 (m, 1H), 2.17 – 1.77 (m, 4H), 1.68 – 1.39 (m, 4H), 0.87 – 0.76 (m, 3H). ^{13}C NMR (100 MHz, DMSO) δ 162.7, 161.4, 148.9, 148.7, 148.4, 148.3, 144.9, 140.7, 140.5, 136.2, 136.2, 135.6, 134.2, 134.1, 134.1, 134.0, 131.6, 124.0, 120.8, 119.6, 117.0, 116.9, 116.3, 115.8, 104.3, 104.2, 102.3, 98.9, 61.2, 61.1, 37.3, 34.3, 33.7, 33.6, 31.2, 31.1, 26.1, 26.0, 25.0, 24.9, 18.9 ESI-HRMS $[M+H]^+$ calculated for $C_{25}H_{26}N_6O_2$: 443.2190, found: 443.2207 HPLC t_{ret} = 5.885 min and 6.538 min (E/Z mixture, purity 97.1 %)

Supplemental References

Bauer, S.M., Gehringer, M., and Laufer, S.A. (2014). A direct enzyme-linked immunosorbent assay (ELISA) for the quantitative evaluation of Janus Kinase 3 (JAK3) inhibitors. Analytical Methods *6*, 8817-8822.

Beaulieu, P.L., Haché, B., and von Moos, E. (2003). A Practical Oxone®-Mediated, High-Throughput, Solution-Phase Synthesis of Benzimidazoles from 1,2-Phenylenediamines and Aldehydes and its Application to Preparative Scale Synthesis. Synthesis *2003*, 1683-1692.

Chen, V.B., Arendall, W.B., III, Headd, J.J., Keedy, D.A., Immormino, R.M., Kapral, G.J., Murray, L.W., Richardson, J.S., and Richardson, D.C. (2010). MolProbity: all-atom structure validation for macromolecular crystallography. Acta Crystallographica Section D *66*, 12-21.

Davis, M.I., Hunt, J.P., Herrgard, S., Ciceri, P., Wodicka, L.M., Pallares, G., Hocker, M., Treiber, D.K., and Zarrinkar, P.P. (2011). Comprehensive analysis of kinase inhibitor selectivity. Nat Biotech *29*, 1046-1051.

Emsley, P., Lohkamp, B., Scott, W.G., and Cowtan, K. (2010). Features and development of Coot. Acta Crystallographica Section D *66*, 486-501.

Evans, P. (2006). Scaling and assessment of data quality. Acta Crystallographica Section D *62*, 72-82.

Frigerio, M., Santagostino, M., and Sputore, S. (1999). A User-Friendly Entry to 2-Iodoxybenzoic Acid (IBX). The Journal of Organic Chemistry *64*, 4537-4538.

Gehringer, M., Forster, M., Pfaffenrot, E., Bauer, S.M., and Laufer, S.A. (2014). Novel Hinge-Binding Motifs for Janus Kinase 3 Inhibitors: A Comprehensive Structure–Activity Relationship Study on Tofacitinib Bioisosteres. ChemMedChem *9*, 2516-2527.

Goedken, E.R., Argiriadi, M.A., Banach, D.L., Fiamengo, B.A., Foley, S.E., Frank, K.E., George, J.S., Harris, C.M., Hobson, A.D., Ihle, D.C.*, et al.* (2015). Tricyclic Covalent Inhibitors Selectively Target Jak3 through an Active Site Thiol. Journal of Biological Chemistry *290*, 4573-4589.

Ireland, R.E., and Liu, L. (1993). An improved procedure for the preparation of the Dess-Martin periodinane. The Journal of Organic Chemistry *58*, 2899-2899.

Kem, K.M., Nguyen, N.V., and Cross, D.J. (1981). Phase-transfer-catalyzed Michaelis-Becker reaction. The Journal of Organic Chemistry *46*, 5188-5192.

Knupp, G., and Frahm, A.W. (1984). Asymmetrische reduktive Aminierung von Cycloalkanonen 2. Synthese und absolute Konfiguration 2-substituierter Cyclohexanamine. Chemische Berichte *117*, 2076-2098.
London, N., Miller, R.M., Krishnan, S., Uchida, K., Irwin, J.J., Eidam, O., Gibold, L., Cimermančič, P., Bonnet, R., Shoichet, B.K., *et al.* (2014). Covalent docking of large libraries for the discovery of chemical probes. Nat Chem Biol *10*, 1066-1072.
Machleidt, T., Woodroofe, C.C., Schwinn, M.K., Méndez, J., Robers, M.B., Zimmerman, K., Otto, P., Daniels, D.L., Kirkland, T.A., and Wood, K.V. (2015). NanoBRET—A Novel BRET Platform for the Analysis of Protein–Protein Interactions. ACS Chemical Biology *10*, 1797-1804.
McCoy, A.J., Grosse-Kunstleve, R.W., Adams, P.D., Winn, M.D., Storoni, L.C., and Read, R.J. (2007). Phaser crystallographic software. Journal of Applied Crystallography *40*, 658-674.
Murshudov, G.N., Skubak, P., Lebedev, A.A., Pannu, N.S., Steiner, R.A., Nicholls, R.A., Winn, M.D., Long, F., and Vagin, A.A. (2011). REFMAC5 for the refinement of macromolecular crystal structures. Acta Crystallographica Section D *67*, 355-367.
Neumann, L., von Konig, K., and Ullmann, D. (2011). HTS reporter displacement assay for fragment screening and fragment evolution toward leads with optimized binding kinetics, binding selectivity, and thermodynamic signature. Methods Enzymol *493*, 299-320.
Powell, H.R., Johnson, O., and Leslie, A.G.W. (2013). Autoindexing diffraction images with iMosflm. Acta Crystallographica Section D *69*, 1195-1203.
Robers, M.B., Dart, M.L., Woodroofe, C.C., Zimprich, C.A., Kirkland, T.A., Machleidt, T., Kupcho, K.R., Levin, S., Hartnett, J.R., Zimmerman, K., *et al.* (2015). Target engagement and drug residence time can be observed in living cells with BRET. Nat Commun *6*, 10091.
Smith, G.A., Uchida, K., Weiss, A., and Taunton, J. (2016). Essential biphasic role for JAK3 catalytic activity in IL-2 receptor signaling. Nat Chem Biol *12*, 373-379.
Soth, M., Hermann, J.C., Yee, C., Alam, M., Barnett, J.W., Berry, P., Browner, M.F., Frank, K., Frauchiger, S., Harris, S., *et al.* (2013). 3-Amido pyrrolopyrazine JAK kinase inhibitors: development of a JAK3 vs JAK1 selective inhibitor and evaluation in cellular and in vivo models. J Med Chem *56*, 345-356.
Tan, L., Akahane, K., McNally, R., Reyskens, K.M.S.E., Ficarro, S.B., Liu, S., Herter-Sprie, G.S., Koyama, S., Pattison, M.J., Labella, K., *et al.* (2015). Development of Selective Covalent Janus Kinase 3 Inhibitors. Journal of Medicinal Chemistry *58*, 6589-6606.
Thoma, G., Drückes, P., and Zerwes, H.-G. (2014). Selective inhibitors of the Janus kinase Jak3—Are they effective? Bioorganic & medicinal chemistry letters *24*, 4617-4621.
Thoma, G., Nuninger, F., Falchetto, R., Hermes, E., Tavares, G.A., Vangrevelinghe, E., and Zerwes, H.-G. (2011). Identification of a Potent Janus Kinase 3 Inhibitor with High Selectivity within the Janus Kinase Family. Journal of Medicinal Chemistry *54*, 284-288.
Zhang, J., Polishchuk, E.A., Chen, J., and Ciufolini, M.A. (2009). Development of an Oxazole Conjunctive Reagent and Application to the Total Synthesis of Siphonazoles. The Journal of Organic Chemistry *74*, 9140-9151.
